# Multielectron Bond Cleavage Processes Enabled by Redox-Responsive Phosphinimide Ligands

**DOI:** 10.1021/acs.inorgchem.3c02307

**Published:** 2023-10-17

**Authors:** Charles C. Winslow, Paul Rathke, Jonathan Rittle

**Affiliations:** Department of Chemistry, University of California, Berkeley, California 94720, United States

## Abstract

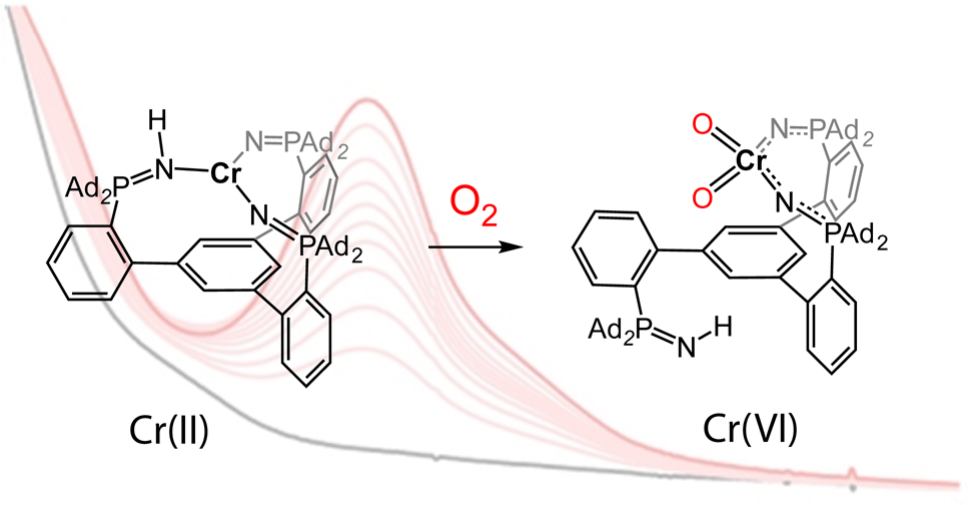

The activation of small molecules via multielectron redox processes offers promise in mediating difficult transformations related to energy conversion processes. While molecular systems that engage in one- and two-electron redox processes are widespread, those that participate in the direct transfer of four or more electrons to small molecules are very rare. To that end, we report a mononuclear Cr^II^ complex competent for the 4-electron reduction of dioxygen (O_2_) and nitrosoarenes. These systems additionally engage in facile two-electron group transfer reactivity, including O atom excision and nitrene transfer. Structural, spectroscopic, and computational studies support bond activation processes that intimately occur at a mononuclear chromium(phosphinimide) center and highlight the unusual structural responsiveness of the phosphinimides in stabilizing a range of metal redox states.

## Abstract 2

While molecular systems that engage in one- and two-electron redox processes are widespread, those that participate in the direct transfer of four or more electrons to small molecules are very rare. We report a mononuclear Cr^II^ complex that mediates the 4-electron reduction of dioxygen (O_2_) and nitrosoarenes. Structural and mechanistic investigations intimate that the electron-donating phosphinimide coligands play active roles in these multielectron bond activation processes. These studies expand the known chemistry of molecular Cr^II^.

## Introduction

Sustainable energy utilization processes will ultimately require systems that can mediate the efficient interconversion of renewable fuel sources and their chemical byproducts. Exemplar fuels—such as CH_4_, CO, NH_3_, and H_2_—require multielectron redox processes for their generation and produce the highest energetic output upon their multielectron combustion.^1−3^ To understand fundamental aspects of these transformations and discover means of alternative fuel and oxidant interconversion processes, synthetic chemists have developed a number of molecular systems that engage in discrete multielectron redox processes.^4−11^ In particular, complexes that feature group 6 transition metals harness the rich redox chemistry intrinsic to these metal ions to activate the strong multiple bonds of kinetically inert gas molecules such as O_2_,^9^ N_2_,^5^ CO,^7^ CO_2_,^11^ and ethylene.^10^ The collective promise of these systems lies in their ability to operate efficiently at ambient temperatures, the potential for generating value-added products from cheap chemical precursors,^7^ and their tunability to optimize new catalytic processes.^6^

**Scheme 1:**
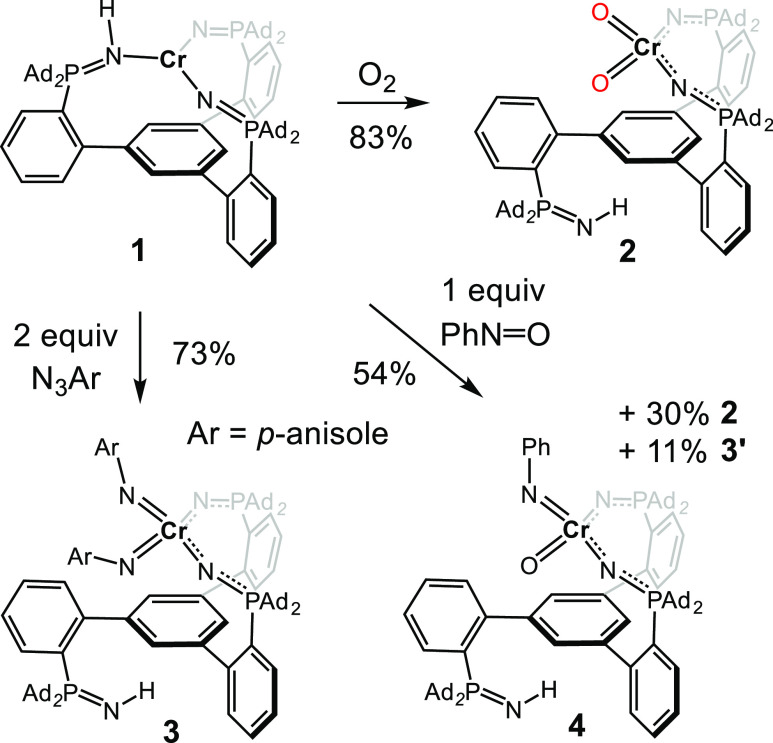
Synthesis of the Featured Cr Complexes

Phosphinimides (PNs) are π-basic ligands that are increasingly recognized to support a wide range of inorganic and organometallic processes. These ligands benefit from pronounced electronic and steric tunability via the selection of appropriate phosphorus substituents. Since the bonding interactions between the P and N atoms are influenced by negative hyperconjugation with the adjacent σ*(C–P) orbitals, the identity of these substituents can markedly influence the electronic properties of N-coordinated metal ions.^12−14^ In conjunction with the usage of sterically encumbering phosphorus substituents, these features have enabled the development of mononuclear Ti-based olefin polymerization catalysts^15,16^ and electron-deficient lanthanide and actinide complexes.^17−19^ We are specifically interested in the utility of PNs to stabilize redox-active, first-row transition-metal complexes and have found that iron and cobalt complexes of tripodal, multidentate PN frameworks engage in a range of single-electron-transfer reactivity.^20−22^ For example, PN-ligated iron(II) species readily mediate the inner sphere one-electron reduction of O_2_,^21^ and stoichiometric oxidation of (PN)-ligated iron(III) species enables hydrogen atom abstraction processes to proceed at the PN nitrogen atom.^22^ In both cases, structural and computational studies evidence perturbations to the metal–PN bonding interactions upon one-electron reduction or oxidation of the complexes, suggesting that these ligands markedly influence the redox properties of their cognate-bound metal ion. Few reports exist on PN-ligated complexes mediating multielectron transformations but include Stryker’s hydrogenation catalysts^23^ and recent work by La Pierre on chalcogen atom transfer to di(iron)–PN^24^ and uranium–PN^19^ complexes. Herein, we report the synthesis and characterization of a divalent chromium–PN compound which exhibits a proclivity toward the four-electron reduction (Scheme 1) of small molecules and readily participates in two-electron group transfer processes. The disclosed reactivity—more commonly observed with Cr(O) and Cr(I) species—places an emphasis on the utility of PN ligation in bolstering the reactivity of bound midvalent metal ions.

## Results and Discussion

**Figure 1:**
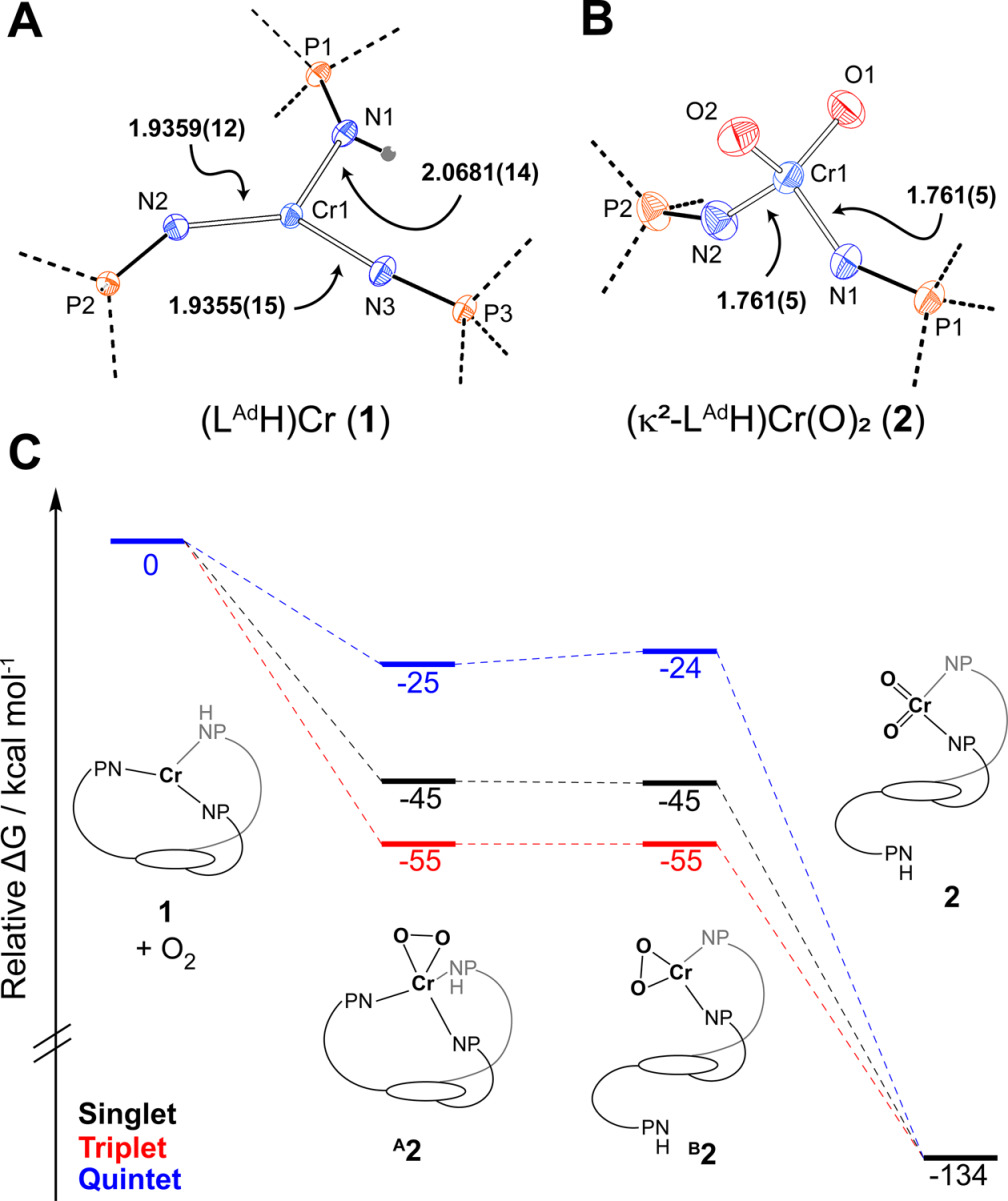
Truncated crystal structures of **1** (A) and **2** (B) highlighting the Cr coordination spheres. Ellipsoids are drawn at 30% probability. (C) BP86/6-31g(d) calculated energy surfaces corresponding to the conversion of **1** to **2**.

Access to a suitable Cr^II^ synthon was accomplished via the reaction of [Cr(HMDS)_2_(THF)_2_]^25^ with the previously reported tris(phosphinimine) proligand decorated with adamantyl substituents on the phosphorus atoms, L^Ad^H_3_,^21^ to furnish [(L^Ad^H)Cr] **1** in an 89% yield. Similar to that found for [(L^Ad^H)Fe], Fourier-transform infrared (FTIR) analysis of solid **1** indicates that one phosphinimine of the ligand remains protonated following divalent metal incorporation, as evidenced by a sharp ν(N–H) band centered at 3403 cm^–1^ observed in KBr pellets of **1**. Solid-state X-ray diffraction (XRD) studies reveal a three-coordinate planar Cr center ligated by two terminally bonded PNs [*d*(Cr–N) = 1.9359(12) and 1.9355(15) Å] and one phosphinimine [*d*(Cr–N) = 2.0681(14) Å] (Figure 1A, see Figure S1 for the complete structure). The Cr center does not appreciably interact with the central arene of the supporting ligand [*d*(Cr–Arene_Centroid_) = 2.924 Å]. In solution, compound **1** adopts an *S* = 2 spin state (μ_eff_ = 4.8 μ_B_, Evans’ method) at room temperature and exhibits broad ^1^H nuclear magnetic resonance (NMR) resonances at 16.60, 13.25, and 5.72 ppm (Figure S2). Akin to other high-spin, three-coordinate Cr^II^ complexes,^26^ the Cr center in compound **1** exhibits a coordination geometry intermediate between that of trigonal planar and T-shaped as evidenced by an obtuse <[N(2)–Cr–N(3)]: 136.0°. Its parallel mode X-band electron paramagnetic resonance (EPR) spectrum collected at 5 K contains an intense feature at *g* = 7.83, which can be modeled as a strongly axial *S* = 2 state (Figure S3). A cyclic voltammogram of **1** in the THF electrolyte exhibits a quasi-reversible oxidation event at −1.24 V vs Fc/Fc^+^ (Figure S4) that we tentatively assign to the Cr^II^/Cr^III^ redox couple. This potential is more reducing than that found for the limited number of other low-coordinate Cr^II^ complexes with available electrochemical parameters.^27,28^ We hypothesize that the low oxidation potential for **1** is a consequence of the electron-releasing nature of the terminal PN ligands.

These molecular features collectively engender compound **1** with facile reactivity toward oxidants. Exposure of a toluene solution of **1** to dry O_2_ at −60 °C leads to the emergence of an intense absorbance band at 458 nm (Scheme 1 and Figure S5) and the disappearance of all paramagnetically shifted features in its ^1^H NMR spectrum. Single-crystal XRD of this red material (Figure 1B, see Figure S6 for the complete structure) indicated that O_2_ is reductively cleaved by **1**, concomitant with phosphinimine dechelation to furnish the pseudo tetrahedral (τ′_4_ = 0.99)^29^ dioxo species, [(κ^2^-L^Ad^H)Cr(O)_2_] **2**. The chromium–oxo groups exhibit bond distances in the expected range for Cr^VI^(O) species [*d*(Cr–O) = 1.591 and 1.599 Å].^30^ Isotopic labeling of the O-atoms in **2** was accomplished via the reaction of **1** with dry ^18^O_2_ and resulted in the shift of two isotopically sensitive bands in the FTIR from 927 and 904 cm^–1^ to 893 and 863 cm^–1^ (Δν = 34 and 41 cm^–1^), respectively (Figure S7). These are in close accordance with the Hooke’s law-predicted shifts of 40 and 39 cm^–1^, supporting the assignment of these modes as the respective asymmetric and symmetric chromium–oxo vibrations.

The formation of [(κ^2^-L^Ad^H)Cr(O)_2_] from **1** represents a rare, formal 4-electron reduction of O_2_ by a *d*^4^ Cr center. O_2_ activation has been investigated with numerous chromium complexes, and the reaction outcome is dependent on the coligands employed: usage of electron-rich amido and/or anilido groups facilitates complete O=O bond rupture,^9,31−35^ whereas the exposure of O_2_ to Cr centers bearing less-donating coligands (e.g., siloxides, amines) generally leads to the formation of partially reduced chromium–superoxide or -peroxide complexes.^36,37^ A handful of Cr^VI^(O)_2_ species have been prepared via the addition of O_2_ to Cr^II^ and Cr^III^ precursors, and these O_2_ activation processes occur via disparate mechanisms. Generation of Cr^VI^(O)_2_ species upon exposure of electron-rich Cr^III^–anilide complexes to O_2_ has been hypothesized to occur via loss of neutral aniline radicals following O_2_ coordination.^31,32^ Dinuclear Cr^II^–bis-amide complexes react with O_2_ via detectable bimetallic intermediates that sequentially split and furnish mononuclear Cr^VI^(O)_2_ species.^32,38^ Theopold has suggested that O_2_ cleavage at mononuclear Cr^I^ sites is spin-forbidden in the case of (NacNac)Cr^I^ and (tris(pyrazolyl)borate)Cr^I^ synthons.^34,35^

In our case, the conversion of **1** to **2** proceeds to completion in ∼10 min at −60 °C in the absence of detectable intermediates observable by UV–visible spectroscopy (Figure S5). Owing to the pronounced steric protection afforded by the four admantyl substituents in the (κ^2^-L^Ad^H)Cr fragment (Figure S8), it is difficult to envision the formation of binuclear [(L^Ad^H)Cr–(O_2_)–Cr(L^Ad^H)] intermediates, and thus we tentatively speculate that the formation of **2** proceeds via sequential O_2_ reduction and O–O bond cleavage at a single metal site. This is reasonable since the filled d orbitals of a *C*_2*v*_-symmetric (PN)_2_Cr^II^ fragment are of the appropriate symmetry to reduce a coordinated O_2_ ligand by four electrons.^4^ Density functional theory (DFT) calculations were performed to gain insight into the multistep conversion of **1** to **2** (Figure 1C). Overall, the conversion of **1** to **2** is highly exergonic (Figure 1C), and this may explain our inability to observe intermediates. Complexation of O_2_ and **1** was found to be exergonic by 55 kcal/mol, and the lowest energy form of the resulting adduct (^**A**^**2**) adopts an *S* = 1 spin state and features a five-coordinate Cr center bound to a side-on bonded (η^2^-O_2_) peroxo ligand akin to that commonly found in other Cr(O_2_) complexes.^37,39,40^ Subsequent dechelation of the neutral phosphinimine ligand was found to proceed with a negligible change in the ground-state energy to afford intermediate ^**B**^**2**. Both ^**A**^**2** and ^**B**^**2** intermediates exhibit long O–O bond distances (1.466–1.490 Å) in their lowest energy spin states that reflect Cr^IV^-peroxo formulations (Figure S9). While the triplet states of ^**A**^**2** and ^**B**^**2** were found to lie lowest in energy, they exhibit small singlet–triplet gaps (gas-phase: Δ*G*_ST_ = ∼ +10 kcal/mol; THF solvated: Δ*G*_ST_ = +7.5 kcal/mol). Owing to these marginal triplet–singlet gaps, we surmise that spin–orbit coupling enables a facile spin state change concomitant with O–O bond rupture to afford **2**.^41^ Nonetheless, while we favor this mononuclear mechanism for O_2_ activation by **1**, we cannot unequivocally rule out alternative mechanisms for O–O bond scission that implicate two or more Cr centers.

**Scheme 2:**
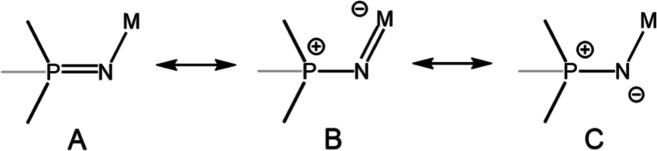
Canonical Resonance Forms of Metallo-Phosphinimide Complexes

The formal 4-electron oxidation of the Cr center from **1** to **2** is accompanied by a noticeable change in the bonding interactions of the PNs. Specifically, the average *d*(Cr–N) distances remarkably contract from 1.936 Å in **1** to 1.761 Å in **2**. The average corresponding *d*(P–N) distances concomitantly increase from 1.535 Å in **1** to 1.570 Å in **2**. In addition, the P–C_Aryl_ bonds found in **2** are ∼0.05 Å shorter than those in **1** (Table S1). These latter differences suggest that the degree of covalency between chromium and nitrogen modulates the hyperconjugation within the σ*(C–P) orbitals.^12^ Collectively, the bonding interactions between the PN ligands and the metal center can be interpreted as a rebalancing of the contributions of resonance structures classically ascribed to metal complexes of these ligands (Scheme 2).^13^ In compound **1**, resonance forms **A** and **C** predominate to account for the P–N multiple bond and Cr–N single bond characters. The contribution of resonance form **B** is increased in compound **2**, serving to simultaneously reduce the P–N bond order and support covalent, multiple bonding within each Cr–N(phosphinimide) interaction.

**Figure 2:**
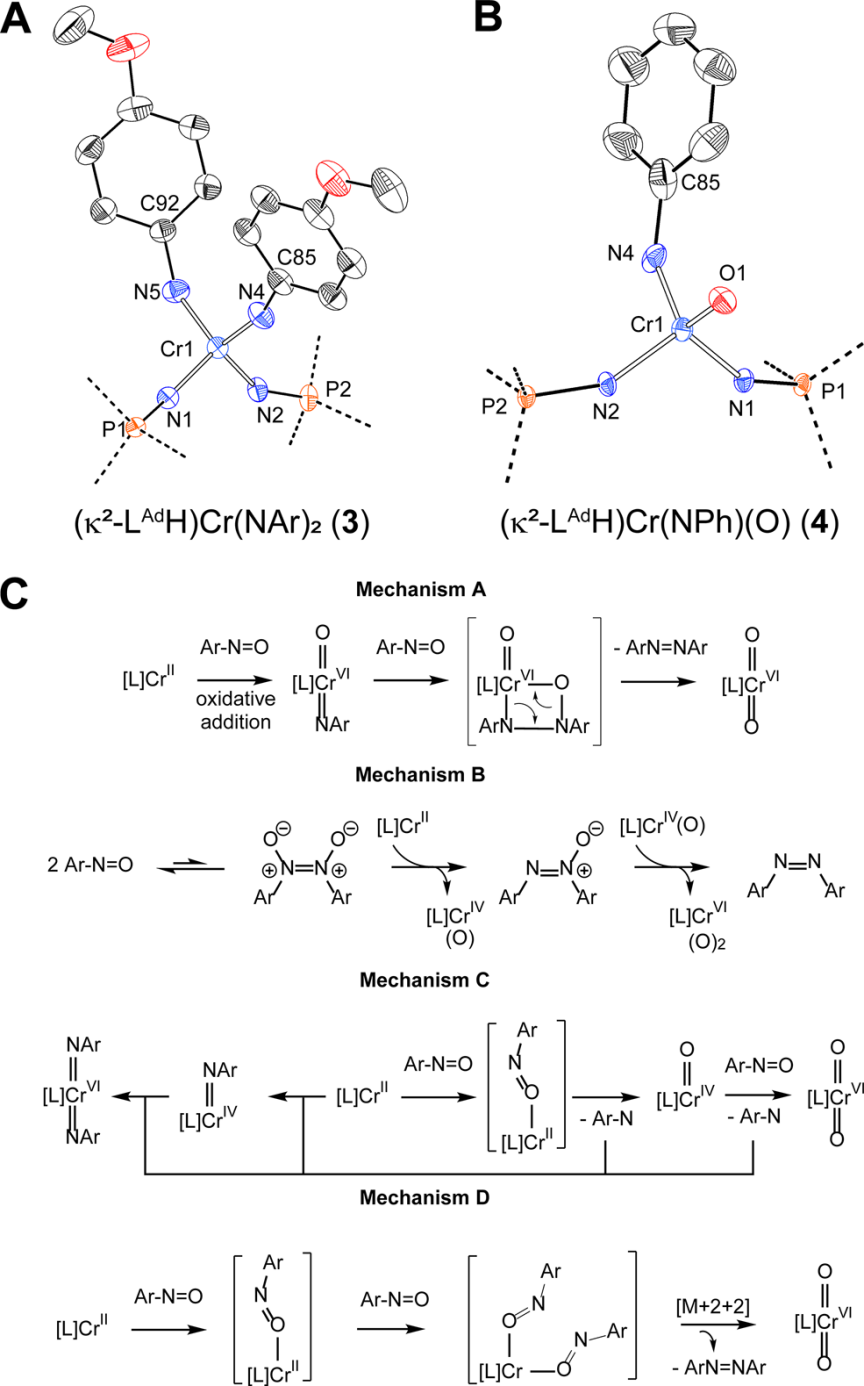
Truncated crystal structures of **3** (A) and **4** (B) highlight the Cr coordination spheres. Ellipsoids are drawn at 30% probability. (C) Possible mechanisms for the formation of **4**, **2** and azoarenes by the reaction of **1** with nitrosoarenes.

Complex **1** was also found to engage in sequential 2-electron group transfer reactivity. The reaction of **1** with two equivalents of aryl azide (aryl = 4-methoxyphenyl) at −78 °C resulted in rapid effervescence (Scheme 1). The ^31^P NMR spectrum (Figure S10) of the reaction product revealed a *C*_*s*_ symmetric diamagnetic product. Single-crystal XRD of this material confirmed its formulation as the bis(imido) complex [(κ^2^-L^Ad^H)Cr(NAr)_2_] **3** (Figure 2A, see Figure S11 for the complete structure), which is isoelectronic and isostructural to compound **2**. Two chemically distinct imido fragments are observed in the solid state with one imido appreciably more bent [<(Cr–N(4)–C(92)): 142.5°] than the other [<(Cr–N(3)–C(85)) = 162.2°]. These distinct imido fragments do not appear to exchange at room temperature, as the ^1^H NMR spectrum of **3** exhibits two inequivalent methoxy groups in a 1:1 ratio (Figure S12). Similar to **2**, the Cr–N(phosphinimide) bonds significantly contract upon oxidation [average *d*(Cr–N) = 1.794(2) Å]. Efforts to prepare Cr^IV^–monoimido species resulted in mixtures of unreacted starting material and **3**, and this observation likely reflects an enhanced stability of the Cr^VI^ oxidation state upon PN and imide ligation.

Attempts to prepare heteroleptic chromium(oxo)(imido) species via nitrosobenzene (PhNO) addition to **1** entailed complex metal speciation, consistent with competing N=O bond cleavage and N=N bond coupling reactions. The combination of **1** with 1 equiv of PhNO at −135 °C (Scheme 1) resulted in an instantaneous reaction that furnished a mixture of three organometallic products as judged by ^31^P NMR spectroscopy of the worked-up solutions. On the basis of the known chemical shifts for **2** and the related diimido **3**, these species appreciably accumulate (30%) **2** and 12% [(κ^2^-L^Ad^H)Cr(NPh)_2_] alongside a major new product (54%) that we ascribe to the heteroleptic [(κ^2^-L^Ad^H)Cr(NPh)(O)] **4** (Figure S13). This latter proposal is supported by an XRD structure obtained from similarly prepared materials (Figure 2B, see Figure S14 for the complete structure), although attempts to purify **4** from contaminating **2** and **3** have not yet been successful. The crystal structure of **4** reveals a terminal oxo ligand and a bent [<(Cr–N–C_Aryl_) = 138.9(4)°] imido group. Short Cr–N(phosphinimide) bonds (Table S1) are observed alongside Cr–O and Cr–N bond distances of 1.605(3) and 1.672(3) Å for the oxo and imido ligands, respectively. The formation of **4** from these reaction mixtures likely arises via the 4-electron reductive cleavage of nitrosobenzene via a route analogous to that for the formation of **2**, which in the case of chromium, has otherwise only been mediated by formal Cr^I^ derivatives.^33^

The curious formation of Cr(O)_2_ complex **2** from the reaction of **1** and PhNO requires the formation of either phenylnitrene or azobenzene in order to balance the reaction stoichiometry. To establish the nature of the organic byproducts, we performed a modified reaction of **1** with (*p*-F–C_6_H_4_)NO and monitored its progression by ^19^F NMR spectroscopy (Figure S15). At room temperature, a solution of two equiv of (*p*-F–C_6_H_4_)NO was slowly added dropwise to **1**, and subsequent NMR analysis indicates the formation of 82% (*p*-F–C_6_H_4_)–N=N–(*p*-F–C_6_H_4_) **5** relative to the (*p*-F–C_6_H_4_)NO employed in the reaction. Under these modified reaction conditions, the ^31^P NMR spectrum revealed **2** as the only observable diamagnetic Cr product (Figure S16). Apparently, when nitrosoarenes are slowly incorporated into the reaction vessel, the formation of the heteroleptic Cr(oxo)(imido) species is minimized and favors N–N bond coupling to form azobenzene **5**.

We consider that this N–N bond formation reaction could proceed via one of several candidate mechanisms (Figure 2C). In an organometallic mechanism (mechanism A), compound **1** first reacts with PhNO to form heteroleptic **4**, and this intermediate subsequently engages a second equivalent of PhNO to furnish compound **2** and azobenzene via heterometathesis of the Cr–N_Imido_ and the O–N_Aryl_ bonds. Similar so-called heterometathesis mechanisms have been observed with molecular group IV imido species^42−46^ and presumably proceed via cyclic intermediates related to those invoked in classical metathesis reactions.^47,48^ Alternatively, N–N bond formation could initially proceed in the absence of Cr via the equilibrium dimerization of nitrosoarenes to form diarylazodioxides ((PhNO)_2_) (mechanism B).^49,50^ In this case, compound **1** could serve to sequentially excise the O atoms of (PhNO)_2_ via group transfer, furnishing the stable **2** and respective azoarenes. A third mechanism can be envisioned (mechanism C) wherein O atoms are directly excised from ArNO by **1** to form free aryl nitrene species and respective Cr(O) species. These free nitrenes could then be captured by available Cr^II^ and/or Cr^IV^ species in solution or alternatively combined bimolecularly to furnish azobenzenes. Finally, we consider a fourth mechanism (mechanism D) that involves sequential coordination of nitrosoarenes to **1** followed by a [M+2 +2] retrocyclization reaction to simultaneously afford **2** and the corresponding azoarene.

The heterometathesis mechanism A does not appear to be operative: exposure of in situ-generated **4** to additional PhNO does not lead to its detectable consumption or generation of additional **2** as judged by ^31^P NMR spectroscopy (Figures S17–S20). Evidence disfavoring mechanism B stems from the reaction of **1** with an electron-rich nitrosoarene that strongly disfavors dimerization and thus is unlikely to generate relevant concentrations of diarylazodioxides.^51^ In this case, the addition of stoichiometric *p*-NMe_2_–C_6_H_4_NO to **1** results in the formation of 82% **2** and an 18% combined yield of species assignable to the *p*-NMe_2_ derivatives of **3** and **4** as determined by ^31^P NMR spectroscopy. In contrast, heteroleptic **4** is the major product upon stoichiometric addition of PhNO to **1**. To probe the transient generation of nitrene intermediates invoked in mechanism C, we explored the reaction of **1** with 2-phenyl-nitrosobenzene. Conversion of this reagent to its singlet nitrene derivative (biphenylnitrene) is expected to initiate a competitive C–H insertion process known to furnish carbazole.^52^ However, carbazole is not detectably generated upon the stoichiometric combination of 2-phenyl-nitrosobenzene and **1** (Figure S21), and this observation sheds doubt on the viability of mechanism C. Owing to the substrate dependence on both the proposed intermediates and the distribution of products observed, it is possible that there are competing mechanisms for azobenzene formation in this system. Otherwise, we cannot account for the variations in product distributions and simultaneously provide an explanation for the consistent generation of small amounts of chromium diimido species in all reaction mixtures. While complete mechanistic elucidation is presently complicated by the difficulty in isolating proposed Cr^IV^ intermediates, the rapid nature of these reactions provides additional support for the pronounced reactivity of compound **1**.

## Conclusions

In summary, a low-coordinate chromium complex supported by sterically encumbered PN ligands was found to engage in the multielectron activation of a series of small molecules. These reactions include highly unusual examples of Cr^II^-mediated O=O and N=O bond cleavage processes that more commonly require formal Cr^I^ synthons. The reactivity and the structural analysis of the inorganic products highlight the ability of PNs to enhance the reducing power of bound low-valent metal ions and stabilize high-valent metal complexes through a modular bonding character. These features collectively enable ready access to tractable systems for O atom excision and formal nitrene transfer processes. Further studies of the ability of these complexes to abstract oxygens from other (in)organic substrates and their subsequent transformations are currently underway.

## Experimental Section

### General Considerations

Unless otherwise noted, all manipulations were carried out using standard Schlenk or glovebox techniques under a N_2_ atmosphere. Acetonitrile (MeCN), benzene, diethyl ether (Et_2_O), pentane, tetrahydrofuran (THF), and toluene were deoxygenated by thoroughly sparging with N_2_ gas followed by passing through an activated alumina column in a solvent purification system from Pure Process Technology and were further dried over 4 Å molecular sieves for 48 h prior to use. Solvents were routinely tested with a THF solution of sodium benzophenone ketyl. Deuterated solvents were purchased from Cambridge Isotope Laboratories, Inc., were distilled under N_2_, degassed via freeze-pump–thaw cycles, and stored over 4 Å molecular sieves prior to use. Oxygen was purchased in ultrahigh purity from Praxair and was further dried by passing through two traps immersed in a dry ice/isopropanol bath. All reagents were purchased from commercial vendors and used without further purification unless otherwise stated. L^Ad^H_3_,^20^ [Cr(HMDS)_2_(THF)_2_],^53^ 4-fluoronitrosobenzene,^54^ and 4,4′-difluoroazobenzene^55^ were prepared according to the literature procedures. Elemental analyses were performed by the Microanalytical Laboratory in the College of Chemistry at the University of California, Berkeley, using a PerkinElmer 2400 Series II combustion analyzer. 97% enriched ^18^O_2_ was obtained in 99.8% chemical purity from Cambridge Isotope Laboratories and was used without further purification.

### Nuclear Magnetic Resonance Spectroscopy

NMR spectra were measured with Bruker AV-300, AVQ-400, NEO-500, or AV-600 spectrometers. ^1^H and ^13^C chemical shifts are reported in parts per million relative to tetramethylsilane (TMS) at 0.00 ppm using residual solvent residues as internal standards. ^31^P{^1^H} chemical shifts are reported in ppm relative to 85% aqueous H_3_PO_4_ at 0 ppm. Solution phase magnetic measurements were performed using the method of Evans.^56^ Coupling constants reported in the ^13^C{^1^H} NMR spectra arise from ^31^P–^13^C interactions.

### Infrared Spectroscopy

Solid IR measurements were obtained on a Nicolet iS20 Spectrometer as KBr pellets.

### X-ray Crystallography

XRD studies were performed at the Small Molecule X-ray Crystallography Facility (CheXray) or at beamline 12.2.1 at the Advanced Light Source at Lawrence Berkeley National Laboratory.

For studies performed at ChexRay: crystals were mounted on a Kapton loop under Paratone oil. Data were collected on a Rigaku XtalLAB P200 (Mo Kα or Cu Kα radiation) equipped with a MicroMax-007 HF microfocus rotating anode and a Pilatus 200 K hybrid pixel array detector at 100 K under a stream of N_2_. Data collection, integration, and scaling were carried out using the CrysAlis^Pro^ software.^57^

For studies performed at the Advanced Light Source: crystals were mounted on a MiTeGen loop under Paratone oil. Data were collected on a Bruker D85 three-circle diffractometer with a PHOTON II CCD area detector using silicon monochromated synchrotron radiation (λ = 0.7288 Å). Bruker APEX2 software was used for data collection. Bruker SAINT and SADABS software was utilized for data reduction and absorption correction, respectively.^58,59^

Structures were solved using SHELXS and refined against F^2^ on all data by full matrix least-squared with SHELXL using OLEX2 crystallographic software.^60^ All non-hydrogen atoms were refined using anisotropic displacement parameters. Hydrogen atoms were placed in idealized positions and refined by using a riding model.

### Electronic Paramagnetic Resonance Spectroscopy

X-band EPR spectra were obtained on a Bruker EMX spectrometer on 5 mM solutions as frozen glasses in toluene. Samples were collected at 2 mW power and a temperature of 5 K with modulation amplitudes of 8 G. Spectra were simulated using the EasySpin^61^ suite of programs in Matlab 2021.

### Optical Spectroscopy

Measurements were taken on a Hewlett-Packard 8453 UV–vis spectrophotometer using a 1 cm quartz cell sealed with a Teflon stopcock. Variable temperature measurements were performed by using a UNISOKU Unispec Cryostat mounted within the spectrophotometer.

### Electrochemistry

Electrochemical measurements were carried out in 0.2 M THF solution of electrolyte ([^*n*^Bu_4_N][PF_6_]). Data collection was performed on a BioLogic SP-50 potentiostat using a freshly polished glassy carbon electron as the working electrode and a platinum wire as the auxiliary electrode. All reported potentials are referenced to the ferrocene–ferrocenium couple (Cp_2_Fe/Cp_2_Fe^+^).

### Density Functional Theory Calculations

All calculations were carried out using Gaussian 09 rev. D.01.^62^ Coordinates for all heavy (non-H) atoms of **1** and **2** were taken from the structures determined by X-ray crystallography. To improve the efficiency of the calculations, the adamantyl substituents were truncated to *tert*-butyl substituents. Gas-phase geometry optimizations and single-point and frequency calculations employed the BP86 functional with a 6-31g(d) basis set employed for all atoms. Successful optimization to an energetic minimum was confirmed by the absence of imaginary frequencies in a subsequent frequency calculation. For solvent-corrected calculations, the SMD protocol was employed using tetrahydrofuran as the solvent.

### Synthetic Procedures

[(L^Ad^H)Cr] (**1**): in the glovebox, a 250 mL round-bottomed flask was charged with a magnetic stir bar, L^Ad^H_3_ (1.50 g, 1.2 mmol), and Et_2_O (50 mL). [Cr(HMDS)_2_(THF)_2_] (0.69 g, 1.2 mmol, 1.0 equiv) was added dropwise to the flask as a solution in Et_2_O (20 mL). The mixture was stirred for 48 h, resulting in a dark brown heterogeneous mixture. The brown solid was collected on a medium frit and washed with Et_2_O (2 × 20 mL) until the washings were colorless. The brown solid residue was extracted into benzene (3 × 15 mL), and the volatiles were removed to give [(L^Ad^H)Cr] (**1**) as a brown powder (1.33 g, 1.0 mmol, 83%). The filtrate was concentrated and layered with pentane to obtain a second crop of **1** (0.05 g, 0.03 mmol, 3%) to furnish a combined yield of 86%. Single crystals of **1** suitable for XRD were grown by layering pentane onto a concentrated Et_2_O solution of **1** at room temperature to produce pale brown rods. ^1^H NMR (400 MHz, C_6_D_6_, 293 K, ppm): 16.60, 13.25, and 5.72. Anal. calcd for C_84_H_106_N_3_P_3_Cr·Et_2_O: C, 76.77; H, 8.49; N 3.05. Found: C, 76.52; H, 8.50; N, 3.09. μ_eff_ (C_6_D_6_, 298 K, 400 MHz): 4.8 μ_B_. IR (KBr, 298 K, cm^–1^): 3403 ν(N–H). UV–visible (toluene, 213 K, nm {cm^–1^ M^–1^}): 385 {1700}.

[(κ^2^-L^Ad^H)CrO_2_] (**2**): in the glovebox, a 50 mL Schlenk tube was charged with a toluene (25 mL) solution of **1** (300 mg, 0.23 mmol, 1 equiv) and a magnetic stir bar. The tube was sealed, removed from the glovebox, and cooled to −78 °C by immersion of the flask in a dry ice/isopropanol bath. The cold solution was stirred, and the tube was evacuated and subsequently exposed to 1 atm of dry O_2_, resulting in the immediate generation of a red solution. The flask was slowly warmed to ambient temperature and stirred for 3 h. The volatiles were removed, and the Schlenk tube was transferred into the glovebox. The tube was extracted with benzene (3 × 10 mL) and subsequently concentrated in vacuo. The residue was washed with Et_2_O (4 mL) and dried furnishing [(κ^2^-L^Ad^H)CrO_2_] (**2**) as a blood red solid (254 mg, 0.19 mmol, 83%). The Et_2_O washings were layered with pentane (4 mL) to produce crystals suitable for XRD (22 mg, 0.02 mmol, 7%) to furnish a combined yield of 90%. ^1^H NMR (400 MHz, C_6_D_6_, 293 K, ppm): 8.46 (d, *J* = 8.0 Hz, 1H), 7.76 (d, *J* = 7.9 Hz, 2H), 7.68 (s, 1H), 7.60 (t, *J* = 8.2 Hz, 1H), 7.48 (t, *J* = 9.0 Hz, 2H), 7.36–7.23 (m, 3H), 7.06 (s, 5H), 2.61–1.40 (m, 90H), and −0.51 (s, 1H; PN*H*). ^13^C{^1^H} NMR (151 MHz, C_6_D_6_): δ 151.1, 150.6 (d, *J* = 7.5 Hz), 141.0, 138.9, 137.5, 135.5 (d, *J* = 8.4 Hz), 134.1 (d, *J* = 9.0 Hz), 131.3 (d, *J* = 11.8 Hz), 131.1 (d, *J* = 6.6 Hz), 129.5, 129.3, 129.0, 125.3, 124.8 (d, *J* = 11.3 Hz), 124.5 (d, *J* = 8.7 Hz), 123.0, 47.6 (d, *J* = 50.1 Hz), 44.5 (d, *J* = 45.9 Hz), 41.5 (d, *J* = 61.2 Hz), 38.5, 38.1, 37.7, 36.8, 36.6, 36.4, 28.7 (d, *J* = 9.3 Hz), and 28.5 (d, *J* = 9.0 Hz). ^31^P{^1^H} NMR (243 MHz, C_6_D_6_, 293 K, ppm): 35.1, 27.4. Anal. calcd for C_84_H_106_N_3_P_3_CrO_2_: C, 75.59; H, 8.01; N, 3.15. Found: C, 75.23; H, 7.99; N, 3.10. IR (KBr, 298 K, cm^–1^): 904 sym ν(O=Cr=O), 927 asym ν(O=Cr=O), 3402 ν(N–H). UV–visible (toluene, 213 K, nm {cm^–1^ M^–1^}): 458 {5200}.

[(κ^2^-L^Ad^H)Cr^18^O_2_]: this isotopologue was prepared analogously to **2** except with 30 mg of **1** (0.023 mmol), 2 mL of toluene, and within a J-Young tube as a reaction vessel to furnish [(κ^2^-L^Ad^H)Cr^18^O_2_] as a blood red solid (24 mg, 0.18 mmol, 78%). IR (KBr, 298 K, cm^–1^): 863 sym ν(O=Cr=O), 893 asym ν(O=Cr=O), 3402 ν(N–H).

[(κ^2^-L^Ad^H)Cr(NAr)_2_] (**3**): in the glovebox, a 20 mL scintillation vial was charged with **1** (100 mg, 0.076 mmol), toluene (7 mL), and a magnetic stir bar. The solution was cooled to −78 °C with a dry ice/isopropanol chilled cold well. A toluene (2 mL) solution of 4-methoxyphenylazide (23 mg, 0.152 mmol, 2.0 equiv) was added dropwise to the stirred solution, resulting in a color change to dark red-brown. The reaction mixture was allowed to warm to room temperature and stirred for 3 h. The solution was concentrated in vacuo, and the residue was triturated with pentane (3 mL) and subsequently dried. The solid was washed with pentane (3 × 2 mL) and then extracted into benzene (3 × 3 mL). The combined benzene fractions were concentrated to ∼5 mL and layered with pentane (12 mL) at room temperature to furnish **2** as a red-brown microcrystalline solid (86 mg, 0.056 mmol, 73%). Single crystals suitable for XRD were grown from slow evaporation of the pentane washes. ^1^H NMR (600 MHz, C_6_D_6_, 293 K, ppm): 8.01–7.96 (m, 2H), 7.81 (m, 1H), 7.69–7.62 (m, 3H), 7.49–7.44 (m, 1H), 7.38 (d, *J* = m, 2H), 7.30 (m, 2H), 7.28–7.22 (m, 4H), 7.14–7.10 (m, 4H), 6.81–6.77 (m, 2H), 6.71–6.66 (m, 2H), 3.37 (s, 3H), 3.19 (s, 3H), 2.73–2.64 (m, 6H), 2.41 (d, *J* = 12.7 Hz, 6H), 2.34 (t, *J* = 12.4 Hz, 12H), 2.25 (t, *J* = 12.4 Hz, 12H), 2.07–2.01 (m, 6H), 1.85–1.79 (m, 18H), 1.72 (d, *J* = 12.2 Hz, 6H), 1.59–1.45 (m, 24H), and −0.46 (s, 1H; PN*H*). ^13^C{^1^H} NMR (151 MHz, C_6_D_6_): δ 158.4, 157.9, 156.2, 155.7, 141.9, 138.0, 135.3 (t, *J* = 9.3 Hz), 132.0 (d, *J* = 7.2 Hz), 131.7 (d, *J* = 11.5 Hz), 129.9, 129.3 (d, *J* = 7.0 Hz), 125.8, 125.1, 124.9 (d, *J* = 11.8 Hz), 124.8, 124.6 (d, *J* = 8.2 Hz), 113.6, 113.1, 55.0, 54.9, 46.4 (d, *J* = 47.8 Hz), 43.6 (d, *J* = 46.8 Hz), 42.0 (d, *J* = 61.4 Hz), 39.3, 38.5, 38.0, 37.3, 37.2, 37.0, 29.5 (d, *J* = 9.2 Hz), and 29.1–28.8 (m). ^31^P{^1^H} NMR (243 MHz, C_6_D_6_, 293 K, ppm): 35.0, 25.4. Anal. calcd for C_98_H_120_N_5_P_3_CrO_2_: C, 76.19; H, 7.83; N, 4.53. Found: C, 75.87; H, 7.59; N, 4.72. IR (KBr, 298 K, cm^–1^): 3413 ν(N–H). UV–visible (toluene, 298 K, nm {cm^–1^ M^–1^}): 382 {3300}.

[(κ^2^-L^Ad^H)Cr(NPh)(O)] (**4**): in the glovebox, a 20 mL scintillation vial was charged with **1** (20 mg, 0.0154 mmol), 2-methyltetrahydrofuran (7 mL), and a magnetic stir bar. The solution was frozen by using a liquid N_2_ cold well. The solution was allowed to thaw and 0.15 mL of a 0.1 M solution of nitrosobenzene in 2-methyltetrahydrofuran was added dropwise immediately after thawing was complete, resulting in a color change from brown to blood red. The reaction was allowed to slowly warm to room temperature for 15 min, and the solvent was subsequently removed in vacuo. The resulting red residue was triturated with pentane (3 mL) and concentrated. The resulting solid was extracted into benzene-*d*_6_ (0.6 mL). NMR analysis of this sample revealed a mixture of 54% title compound contaminated with 30% **2** and 12% of a species assigned as [(κ^2^-L^Ad^H)Cr(NPh)]. Slow evaporation of the pentane washes simultaneously afforded single crystals of **2** and the title compound **4** suitable for XRD analysis. ^31^P{^1^H} NMR (243 MHz, C_6_D_6_, 293 K, ppm): δ 35.2, 26.7.

N–N coupling of 4-fluoronitrosobenzene mediated by **1**: a mixture of 1-fluoro-4-nitrosobenzene (10.4 mg, 0.0831 mmol) and 4-fluorotoluene (30.2 mg, 0.274 mmol) was dissolved in 3 mL of toluene, affording a stock solution of the following concentrations, respectively, 91.4 and 27.7 mM. Compound **1** (15 mg, 0.012 mmol) was dissolved in 0.83 mL of this stirred solution at room temperature, resulting in an instant color change to dark red. This solution was stirred for an additional 5 min and transferred to an NMR tube. The NMR sample was shimmed using gradient shimming on the ^1^H methyl toluene peak, and the ^19^F spectrum was collected with a prescan delay of 30 s to account for the *T*1 relaxation time of the NMR standard. An integration ratio of 0.24/1.00 between 4,4′-diflouoroazobenzene and the 4-fluorotoluene standard was found, which corresponds to a spectroscopic yield of 79% relative to the nitrosobenzene starting material.

## Supporting Information Available

NMR, FT-IR and UV/visible spectra; X-ray diffraction table; complete structures of **1-4**; EPR spectrum of **1**; Tabulated bond distances and angles; additional synthetic and computional details (PDF)
