# Supplementary material for: Multielectron Bond Cleavage Processes Enabled by Redox-Responsive Phosphinimide Ligands
Source: Inorg Chem. 2023 Oct 17;62(43):17697–704. doi: 10.1021/acs.inorgchem.3c02307 (PMC10618924; doi:10.1021/acs.inorgchem.3c02307)
Supplement: Supplementary file 1 — ic3c02307_si_001.pdf [file ic3c02307_si_001.pdf]

SUPPORTING INFORMATION FOR:

Multielectron Bond Cleavage Processes Enabled by Redox-Responsive Phosphinimide Ligands

Charles C. Winslow, Paul Rathke, Jonathan Rittle

Department of Chemistry, University of California, Berkeley, California 94720, United States

Synthesis of 4-Fluoroazobenzene mediated by ($L^{Ad}H$)Cr (1)

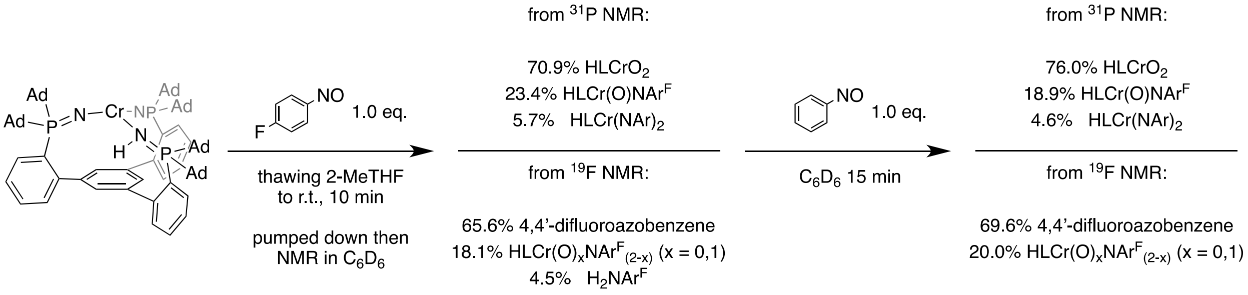

Procedure: ($L^{Ad}H$)Cr (20 mg, 0.0154 mmol) was dissolved in 2-MeTHF (5 mL) in a scintillation vial equipped with a stir bar and the resulting vial was placed within a liquid nitrogen cooled cold well until the solution froze. Subsequently, the vial was removed from the cold well and as the solution began to thaw, 0.15 ml of a 0.1 M 4-fluoronitrosobenzene (0.015 mmol) solution in 2-MeTHF was added dropwise which caused an immediate color change to red. The resulting solution was stirred for 5 minutes after which the solvent was removed *in vacuo* leaving a red film that was subsequently taken up in a 0.5 ml of 0.03 M solution of 4-fluorotoluene (serving as an internal ^{19}F NMR standard) in C_6D_6. ^{31}P and ^{19}F NMR analysis revealed the intermediate composition shown above. Subsequently, 0.15 ml of 0.1 M solution of nitrosobenzene in C_6D_6 was added to the sample at room temperature. NMR analysis performed after 15 min revealed marginal changes to the composition of the sample.

Reaction of ($L^{Ad}H$)Cr and (2-phenyl)nitrosobenzene

Procedure: ($L^{Ad}H$)Cr (20 mg, 0.0154 mmol) was dissolved in 2-MeTHF (5 mL) in a scintillation vial equipped with a stir bar and the resulting vial was placed within a liquid nitrogen cooled cold well until the solution froze. Subsequently, the vial was removed from the cold well and as the solution began to thaw, 0.15 ml of a 0.1 M 2-phenylnitrosobenzene (0.015 mmol) solution in 2-MeTHF was added dropwise which caused an immediate color change to red. The resulting solution was stirred cold for 5 minutes and then stirred for an additional 20 minutes at room temperature. The solution was filtered through a plug of basic alumina to remove organometallic species. An aliquot of the filtrate was analyzed by GC-MS (Figure S21) for the formation of nitrosoarenes and carbazole. An Agilent 7890 GC-MS system equipped with an HP-5MS Agilent column (30m × 0.250 mm × 0.25 μm) was used for GC-MS analysis.

[(L^{Ad}H)Cr] **1**

Distances				Angles	
Cr1-N1	2.0681(14)			N1-Cr1-N2	121.60(5)
Cr1-N2	1.9359(12)			N2-Cr1-N3	135.97(6)
Cr1-N3	1.9355(15)			N3-Cr1-N1	102.26(6)
N1-P1	1.5944(13)			Cr1-N1-P1	137.69(8)
N2-P2	1.5366(13)			Cr1-N2-P2	143.94(8)
N3-P3	1.5334(15)			Cr1-N3-P3	165.57(9)
P1-C8	1.8506(14)				
P1-CAd_Avg	**1.8764(10)**	1.8738(14)	1.8790(15)		
P2-C14	1.8644(14)				
P2-CAd_Avg	**1.9052(10)**	1.8943(15)	1.9160(14)		
P3-C20	1.8739(14)				
P3-CAd_Avg	**1.9055(11)**	1.8990(15)	1.9120(15)		

[(L^{Ad}H)Cr(NAr)$_2$] **3**

Distances				Angles	
Cr1-N1	1.775(2)			N1-Cr1-N2	108.1(1)
Cr1-N2	1.812(2)			N3-Cr1-N4	106.32(13)
Cr1-N4	1.655(3)				
Cr1-N5	1.714(3)			Cr1-N1-P1	156.17(13)
N1-P1	1.554(2)			Cr1-N2-P2	153.32(15)
N2-P2	1.563(2)				
N3-P3	1.5748(19)			Cr1-N4-C85	162.2(2)
P1-C8	1.838(2)			Cr1-N5-C	142.5(2)
P1-CAd_Avg	**1.887(1)**	1.877(2)	1.877(2)		
P2-C14	1.839(3)				
P2-CAd_Avg	**1.885(2)**	1.877(3)	1.892(3)		
P3-C20	1.860(2)				
P3-CAd_Avg	**1.888(1)**	1.893(2)	1.883(2)		

[(L^{Ad}H)Cr(O)$_2$] **2**

Distances				Angles	
Cr1-N1	1.761(4)			N1-Cr1-N2	105.2(2)
Cr1-N2	1.761(4)			O1-Cr1-O2	109.2(2)
Cr1-O1	1.591(4)				
Cr1-O2	1.599(4)			Cr1-N1-P1	150.1(3)
N1-P1	1.569(4)			Cr1-N2-P2	151.3(3)
N2-P2	1.571(4)				
N3-P3	1.565(4)				
P1-C8	1.816(5)				
P1-CAd_Avg	**1.869(4)**	1.863(6)	1.874(5)		
P2-C14	1.824(5)				
P2-CAd_Avg	**1.866(4)**	1.866(5)	1.865(6)		
P3-C20	1.851(5)				
P3-CAd_Avg	**1.865(5)**	1.859(7)	1.871(7)		

[(L^{Ad}H)Cr(O)NPh)] **4**

Distances				Angles	
Cr1-N1	1.776(4)			N1-Cr1-N2	109.88(14)
Cr1-N2	1.774(4)			O1-Cr1-N4	108.56(16)
Cr1-O1	1.605(3)				
Cr1-N4	1.672(3)			Cr1-N1-P1	150.29(17)
N1-P1	1.575(5)			Cr1-N2-P2	153.89(17)
N2-P2	1.568(4)				
N3-P3	1.554(3)			Cr1-N4-C	139.2(3)
P1-C8	1.831(4)				
P1-CAd_Avg	**1.866(3)**	1.865(5)	1.867(4)		
P2-C14	1.838(3)				
P2-CAd_Avg	**1.871(3)**	1.877(4)	1.865(5)		
P3-C20	1.834(3)				
P3-CAd_Avg	**1.870(4)**	1.866(5)	1.873(6)		

Table S1. Salient bond distances and angles determined from the solid-state structures of the reported compounds

X-Ray Crystallography

Identification code	**1** ($L^{Ad}H$)Cr	**2** (κ^2-$L^{Ad}H$)CrO_2	**3** (κ^2-$L^{Ad}H$)Cr$(NAr)_2$	**4** (κ^2-$L^{Ad}H$)Cr(NPh)(O)
Empirical formula	$C_{90}H_{121}CrN_3O_{1.5}P_3$	$C_{86.5}H_{112}CrN_3O_2P_3$	$C_{100}H_{125}CrN_5O_{2.5}P_3$	$C_{97.5}H_{129}CrN_4OP_3$
Formula weight	1413.80	1370.69	1581.95	1517.95
Temperature/K	293(2)	173	173	100
Crystal system	monoclinic	triclinic	monoclinic	triclinic
Space group	$P2_1/c$	P-1	$P2_1/c$	P-1
a/Å	10.21640(10)	15.6398(6)	25.63654(18)	15.6394(8)
b/Å	27.68330(10)	16.4003(5)	14.36215(8)	16.3015(8)
c/Å	26.95690(10)	16.6888(5)	22.45210(11)	19.3066(10)
α/°	90	85.821(3)	90	66.015(2)
β/°	96.0400(10)	68.973(3)	96.5168(6)	82.732(2)
γ/°	90	63.917(4)	90	63.617(2)
Volume/$Å^3$	7581.72(9)	3570.2(2)	8213.36(8)	4019.9(4)
Z	4	2	4	2
$\rho_{calc}g/cm^3$	1.239	1.275	1.279	1.254
μ/mm^{-1}	2.220	2.348	2.125	0.270
F(000)	3052.0	1474.0	3396.0	1638.0
Crystal size/mm^3	0.4 × 0.15 × 0.1	0.25 × 0.2 × 0.2	0.4 × 0.15 × 0.1	0.25 × 0.2 × 0.1
Radiation	Cu Kα (λ = 1.54184)	Cu Kα (λ = 1.54184)	Cu Kα (λ = 1.54184)	synchrotron (λ = 0.7288)
2Θ range for data collection/°	6.386 to 158.16	5.702 to 137.132	6.94 to 158.164	3.936 to 57.522
Index ranges	$-12 \le h \le 10$, $-34 \le k \le 35$, $-34 \le l \le 34$	$-17 \le h \le 18$, $-19 \le k \le 19$, $-19 \le l \le 19$	$-32 \le h \le 32$, $-18 \le k \le 18$, $-26 \le l \le 28$	$-20 \le h \le 20$, $-21 \le k \le 21$, $-25 \le l \le 25$
Reflections collected	135320	98217	241550	134704
Independent reflections	16061 [R_{int} = 0.0523, R_{sigma} = 0.0253]	12850 [R_{int} = 0.0842, R_{sigma} = 0.0459]	17631 [R_{int} = 0.0434, R_{sigma} = 0.0176]	19118 [R_{int} = 0.1064, R_{sigma} = 0.0703]
Data/restraints/parameters	16061/54/918	12850/186/969	17631/54/1114	19118/1269/1059
Goodness-of-fit on F^2	1.064	1.059	1.029	1.024
Final R indexes [I≥2σ (I)]	R_1 = 0.0388, wR_2 = 0.1065	R_1 = 0.0921, wR_2 = 0.2418	R_1 = 0.0626, wR_2 = 0.1845	R_1 = 0.0898, wR_2 = 0.2277
Final R indexes [all data]	R_1 = 0.0404, wR_2 = 0.1078	R_1 = 0.1293, wR_2 = 0.2713	R_1 = 0.0669, wR_2 = 0.1890	R_1 = 0.1220, wR_2 = 0.2482
Largest diff. peak/hole / e $Å^{-3}$	0.55/-0.47	0.76/-0.75	2.11/-1.21	1.51/-1.20

Table S2. X-ray diffraction table.

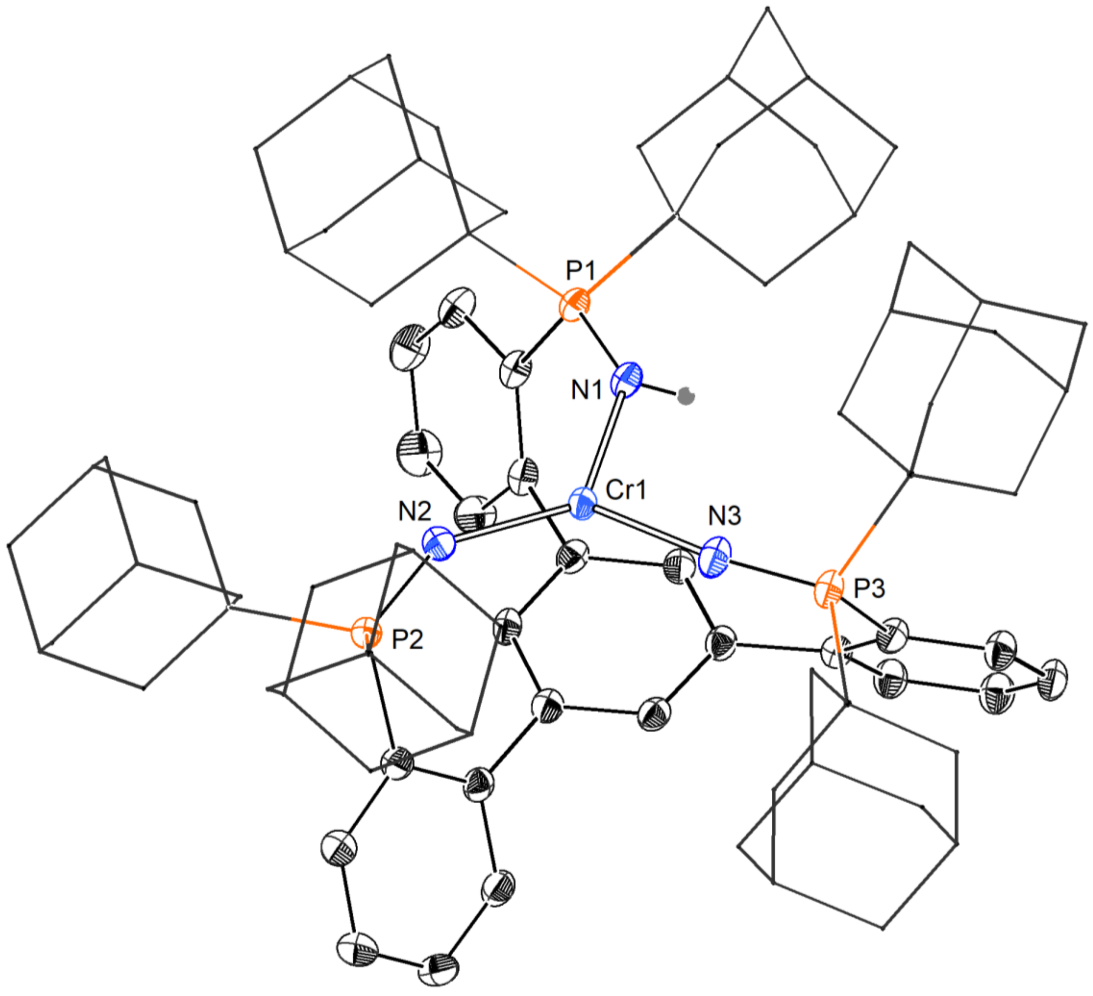

Figure S1. Complete structure of $(L^{Ad}H)Cr$ determined by X-ray diffraction.

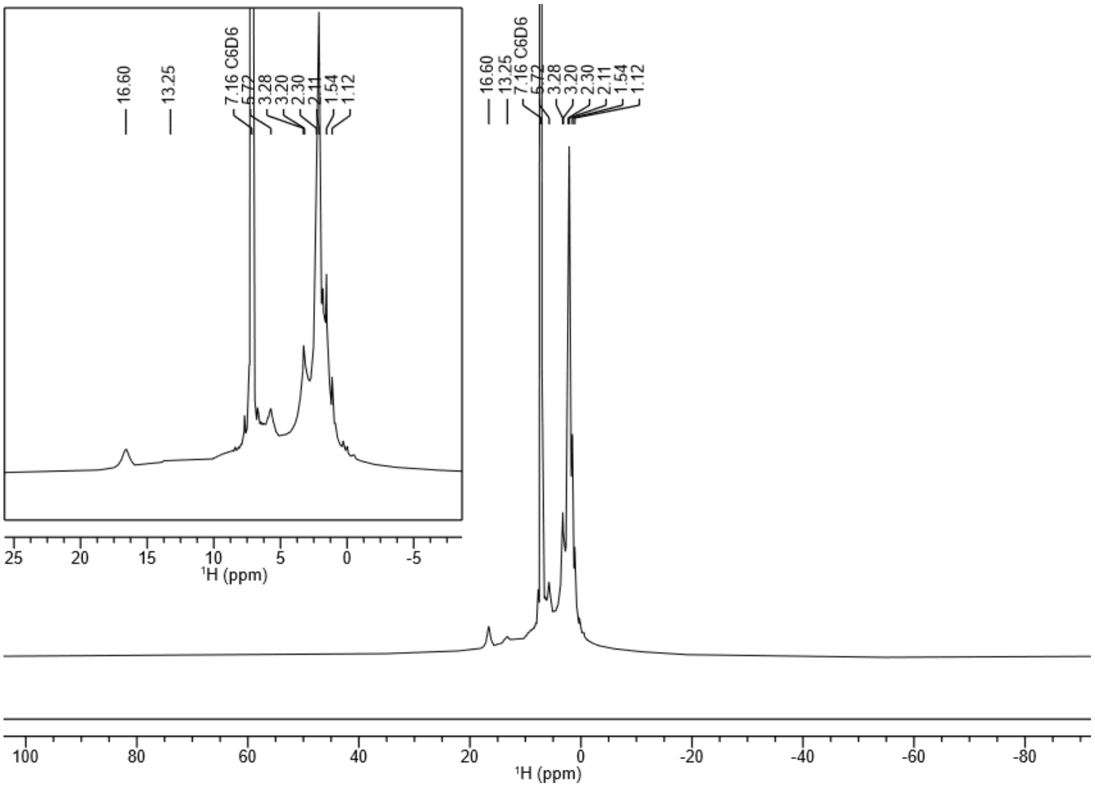

Figure S2. ^{1}H NMR spectrum (400 MHz, C_6D_6, 298K) of **1**.

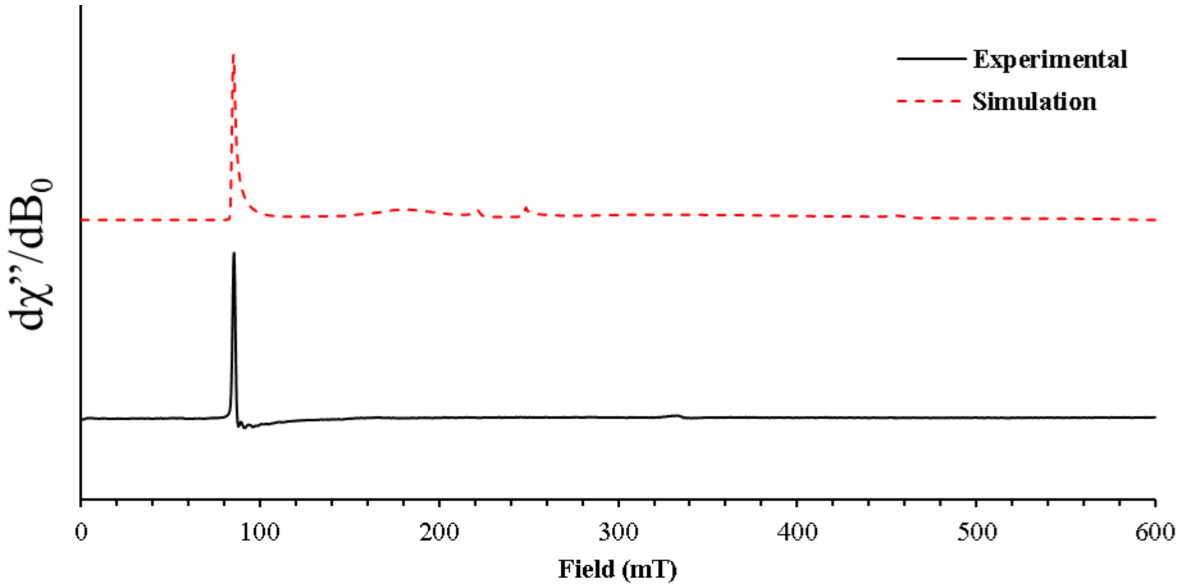

Figure S3. Parallel-mode X-band EPR spectrum of a toluene solution of **1** at 5 K and the $S = 2$ (D = 0.17 cm^{-1}, E = 2.9 x 10^{-4} cm^{-1}, $g = 2.00$) simulation.

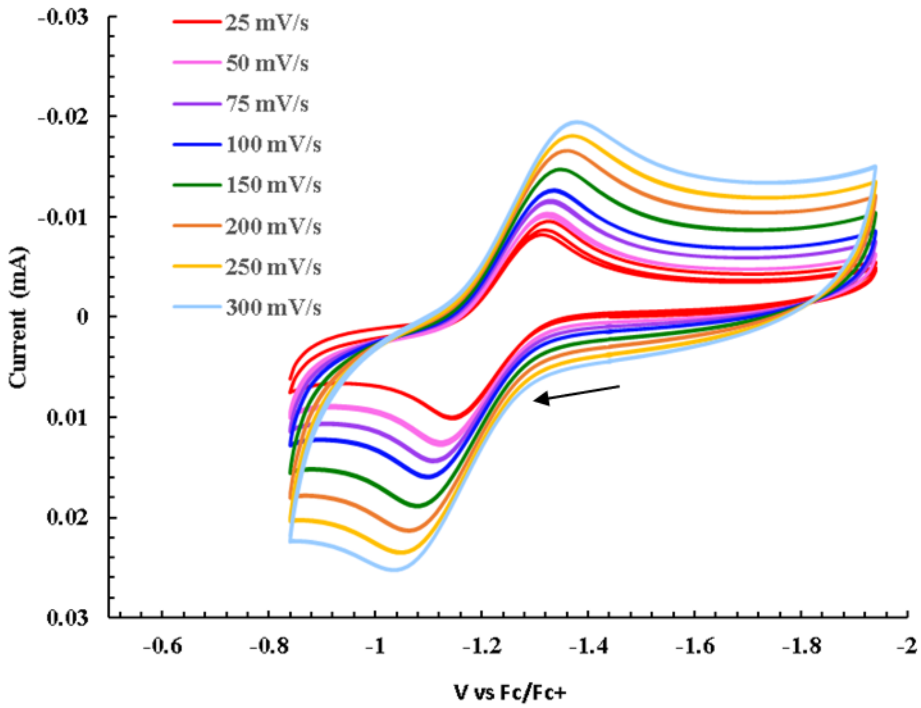

Figure S4. Cyclic voltammogram of **1**, measured in 0.2 M [$^{n}Bu_4N$)[PF_6) electrolyte in THF at 298 K at the listed scan rates. The open circuit potential of **1** was found to be -1.44 V.

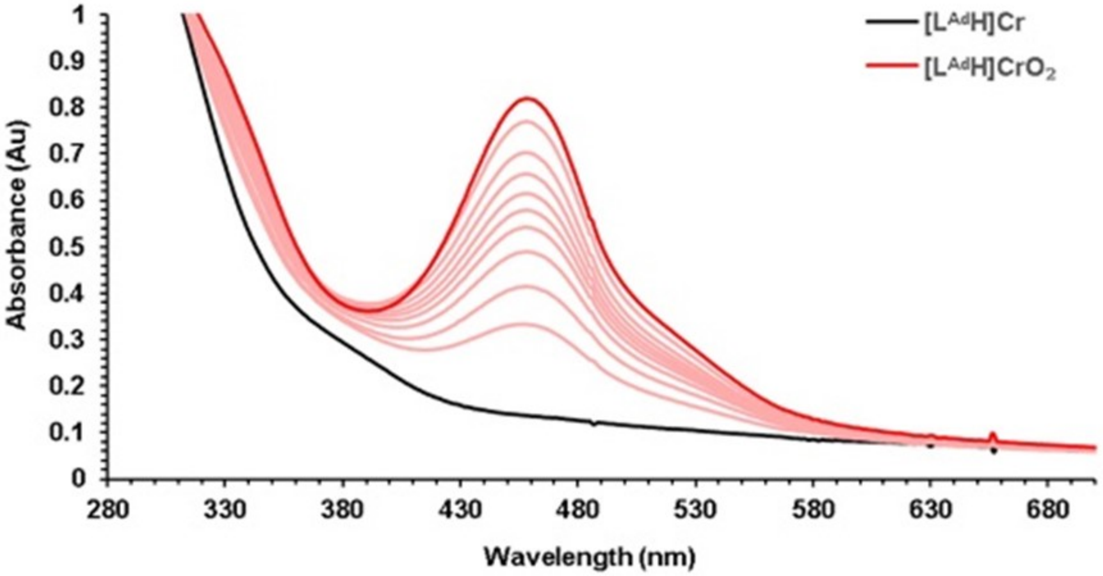

Figure S5. UV-Visible spectra of the reaction of **1** (black) with dry O_2 to produce **2** (red) at 213K in toluene. Each trace represents a 1-minute time point.

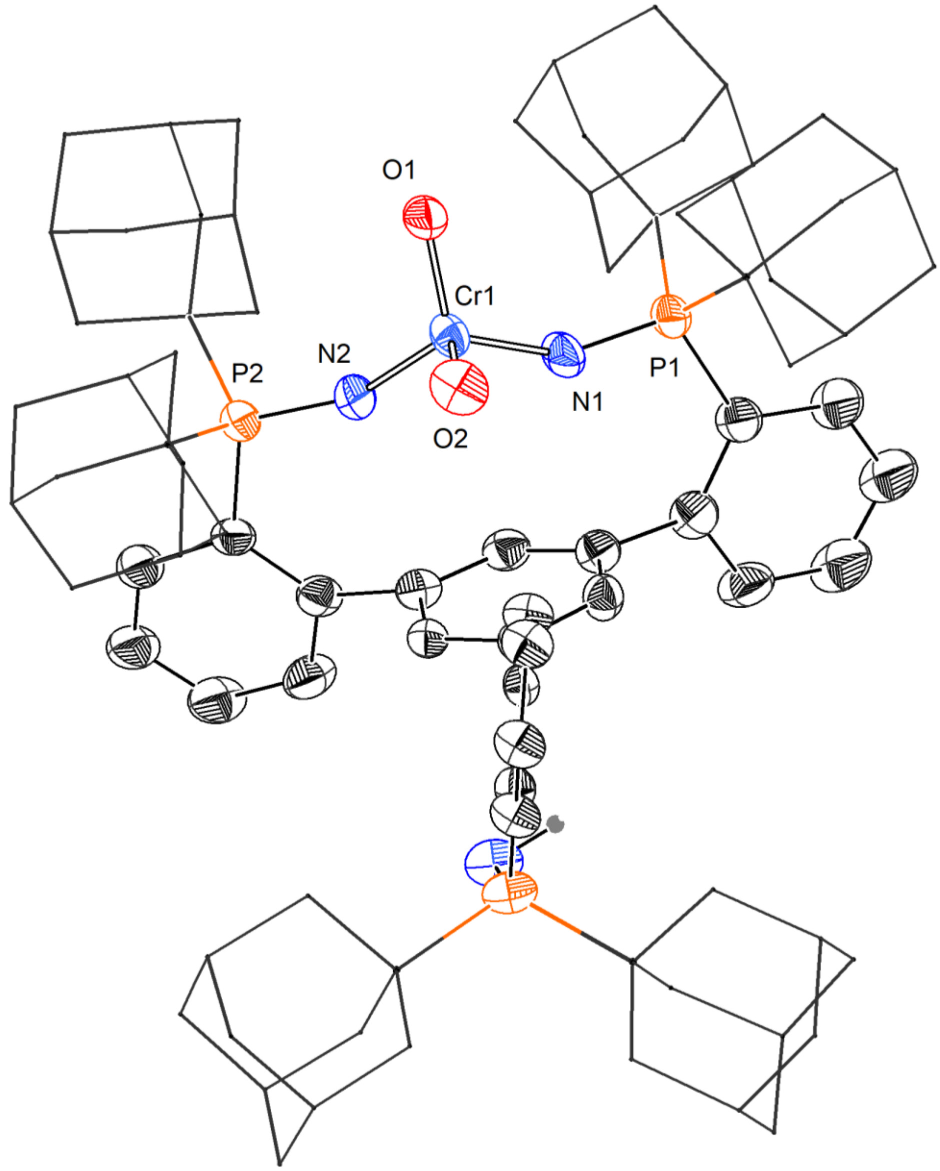

Figure S6. Complete structure of (κ^2-L^{Ad}H)Cr(O)$_2$ **2** determined by X-ray diffraction.

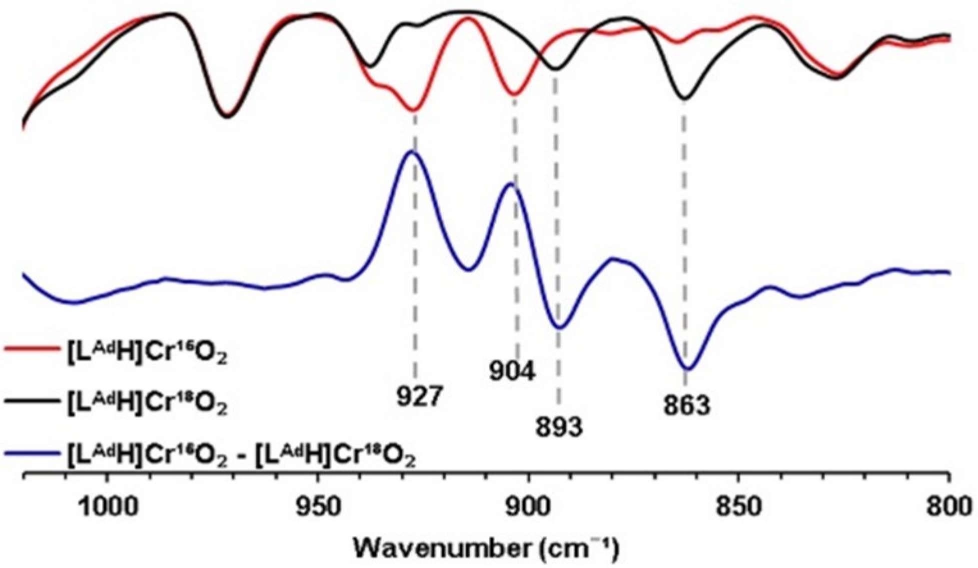

Figure S7. KBr-FTIR of $^{16}O_2$-**2** (red), $^{18}O_2$-**2** (black), and the difference spectrum (blue) at 298K.

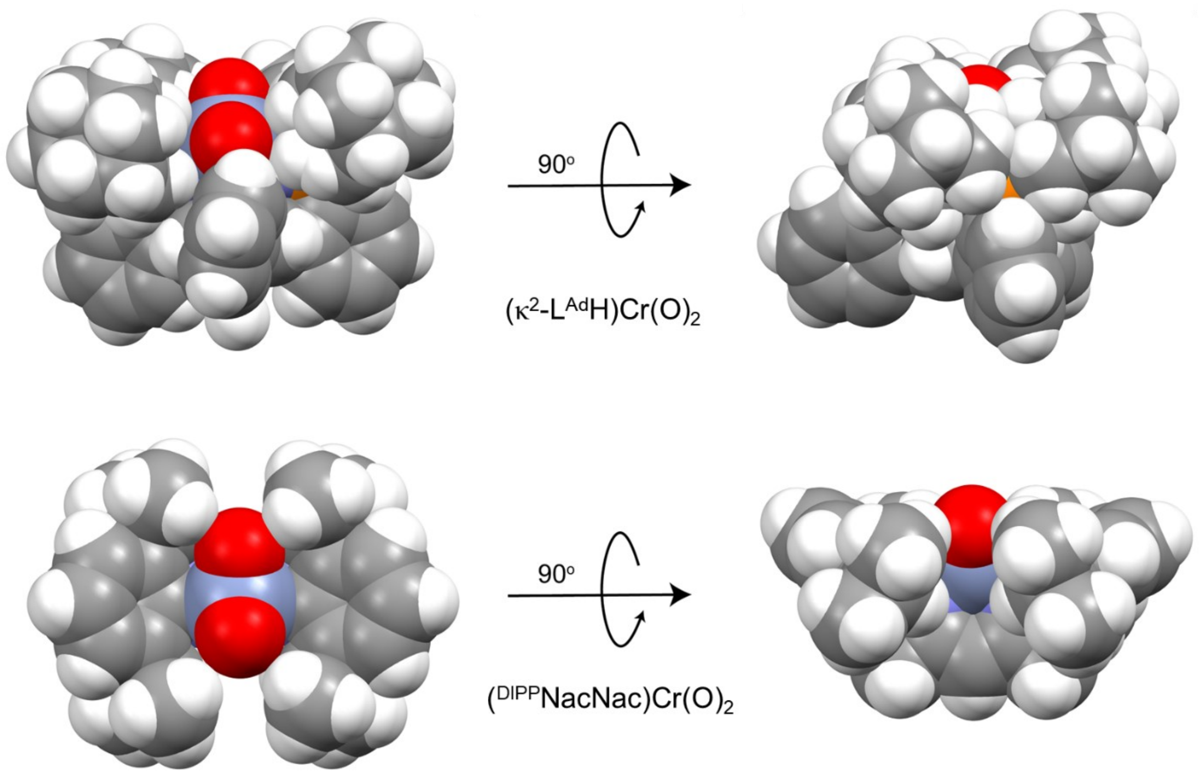

Figure S8. Space-filling representations of the (κ^2-L^{Ad}H)Cr fragment of compound **2** (top) in comparison to that of Theopold's (NacNac)Cr^{V}(O)$_2$ complex (bottom).

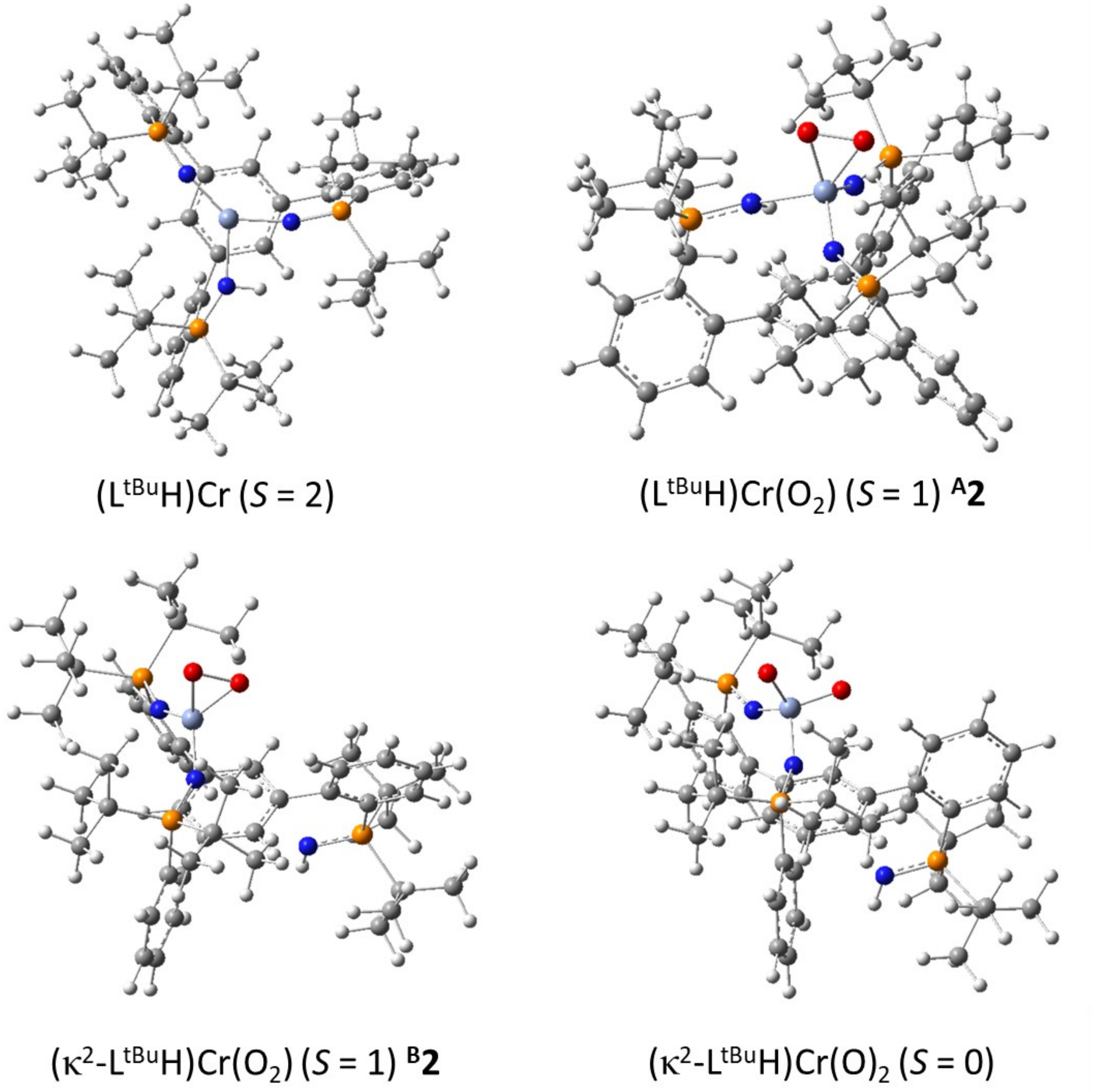

Figure S9. Gas-phase optimized structures of $(L^{tBu}H)Cr$, ^{A}**2**, ^{B}**2**, and $(L^{tBu}H)Cr(O)_2$ in the listed spin states.

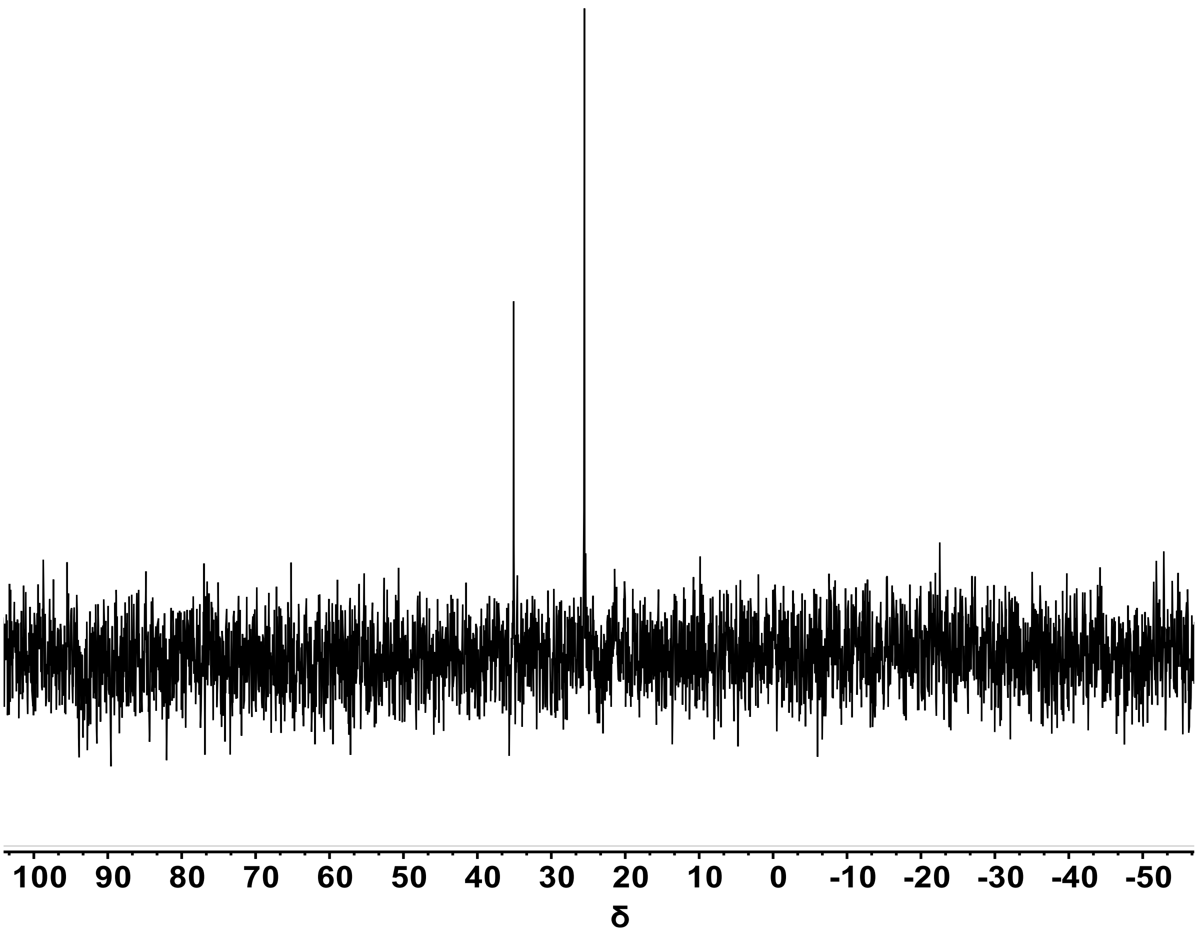

Figure S10. $^{31}P\{^{1}H\}$ NMR spectrum (162 MHz, 298K, C_6D_6) of **3**.

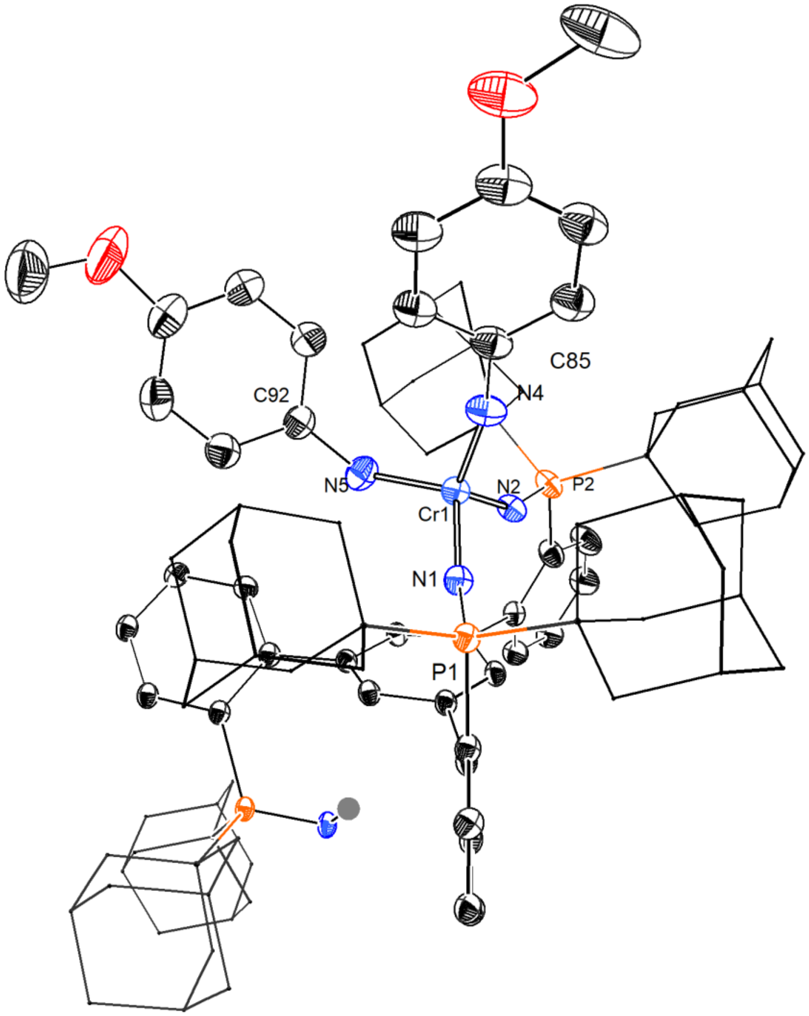

Figure S11. Complete structure of (κ^2-L^{Ad}H)Cr(NAr)$_2$ **3** determined by X-ray diffraction (Ar = *p*-OMe-C$_6$H$_4$)

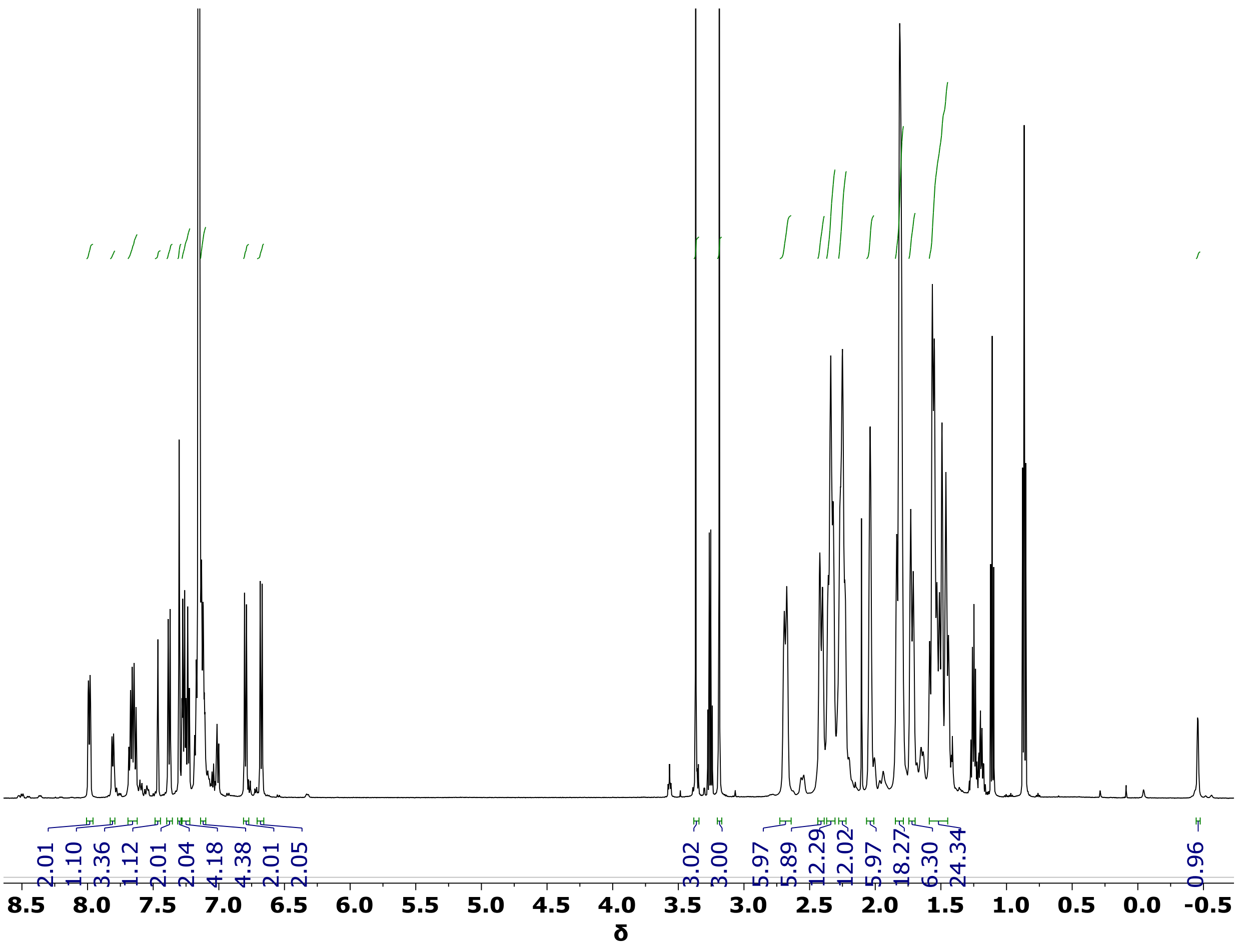

Figure S12.^{1}H NMR spectrum (600 MHz, 298K, C_6D_6) of **3**.

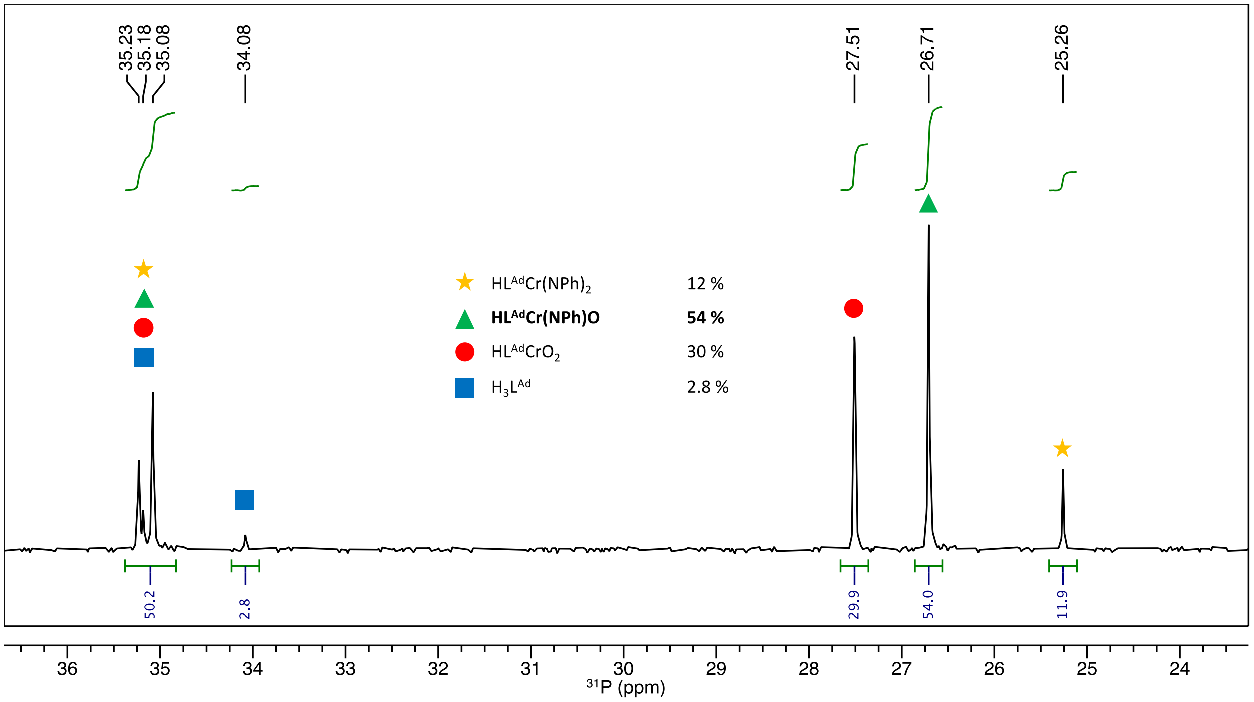

Figure S13. $^{31}P\{^{1}H\}$ NMR (162 MHz, 298 K, C_6D_6) obtained by the method described above illustrating a mixture of **2** (30%), **4** (54%) and a species assigned as $[L^{Ad}H]Cr(NPh)_2$ on the basis of its chemical shift similarity to compound **3** (12%).

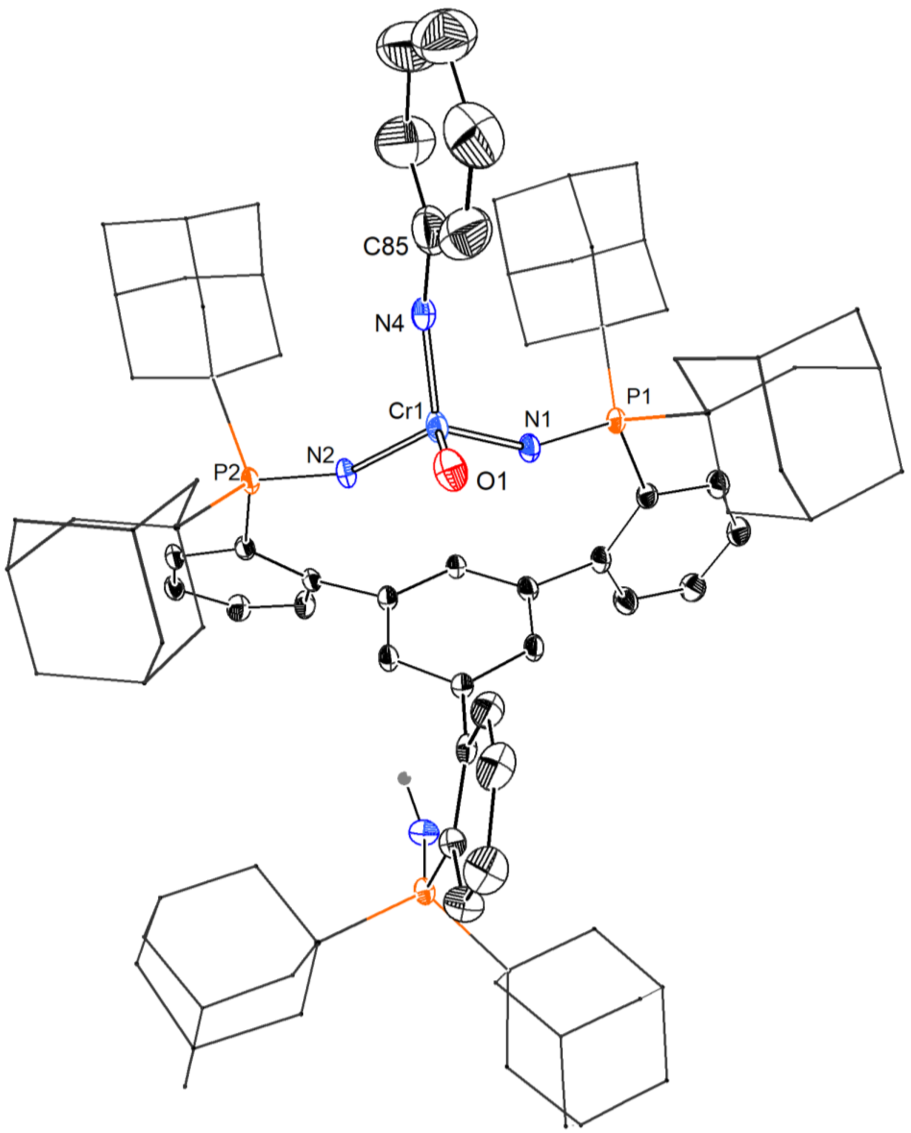

Figure S14. Complete structure of (κ^{2}-L^{Ad}H)Cr(NPh)(O) **4** determined by X-ray diffraction.

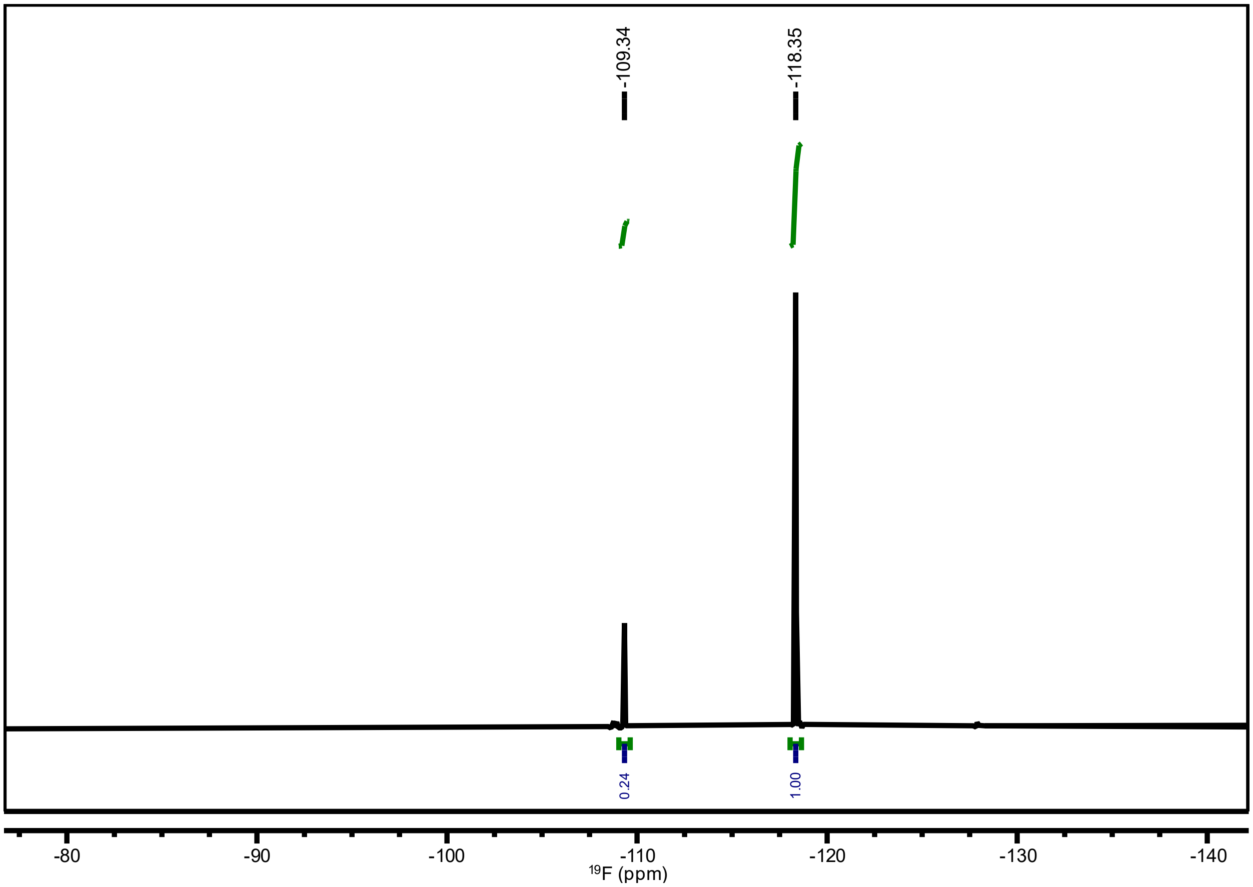

Figure S15. ^{19}F NMR spectrum (376 MHz, *proteo*-toluene, 298K) of the reaction of **1** and 2 equivalents of 4-fluoronitrosobenzene. The 4,4'-F_2-azobenzene resonance is at -110 ppm and the 4-fluorotoluene standard is at -118 ppm.

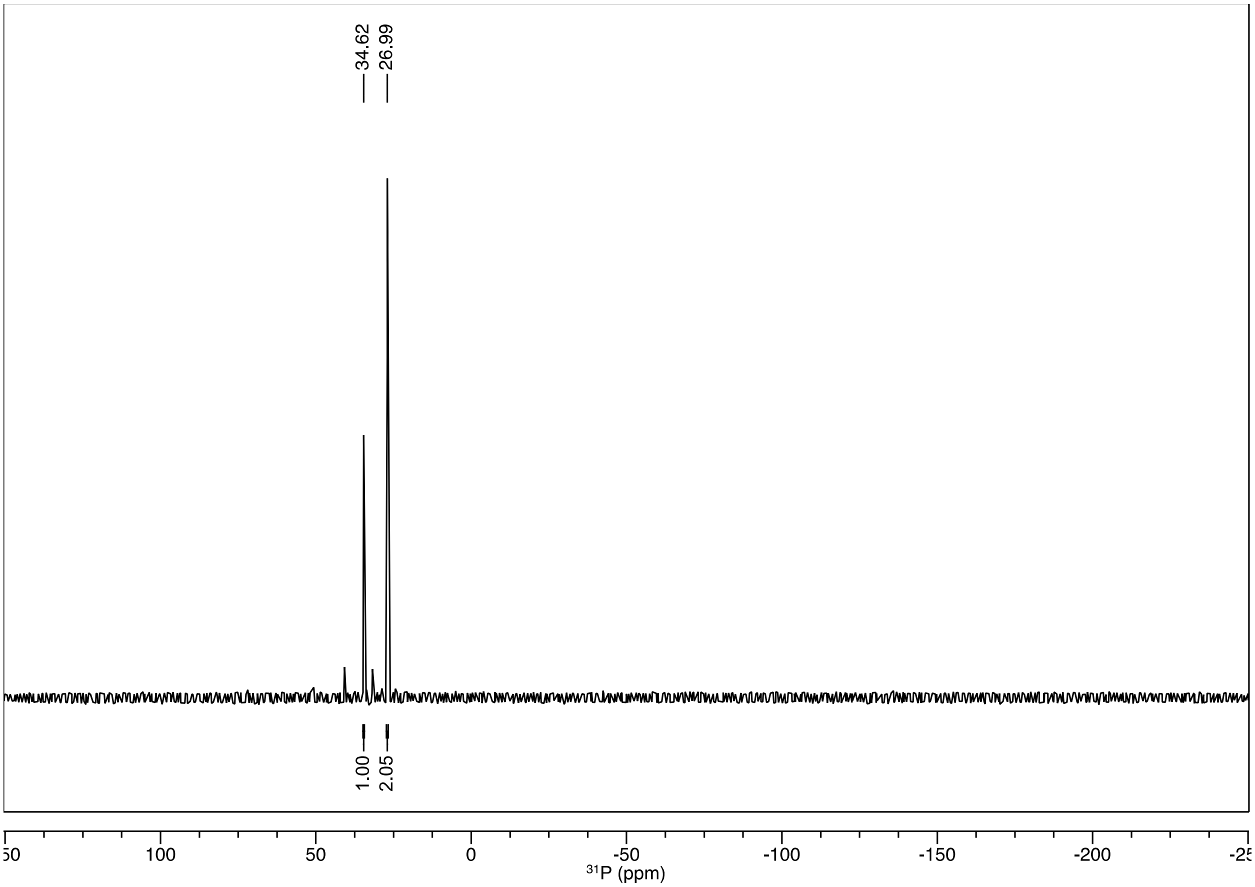

Figure S16. ^{31}P NMR spectrum (162 MHz, *proteo*-toluene, 298K) of the reaction of **1** and 2 equivalents of 4-fluoronitrosobenzene.

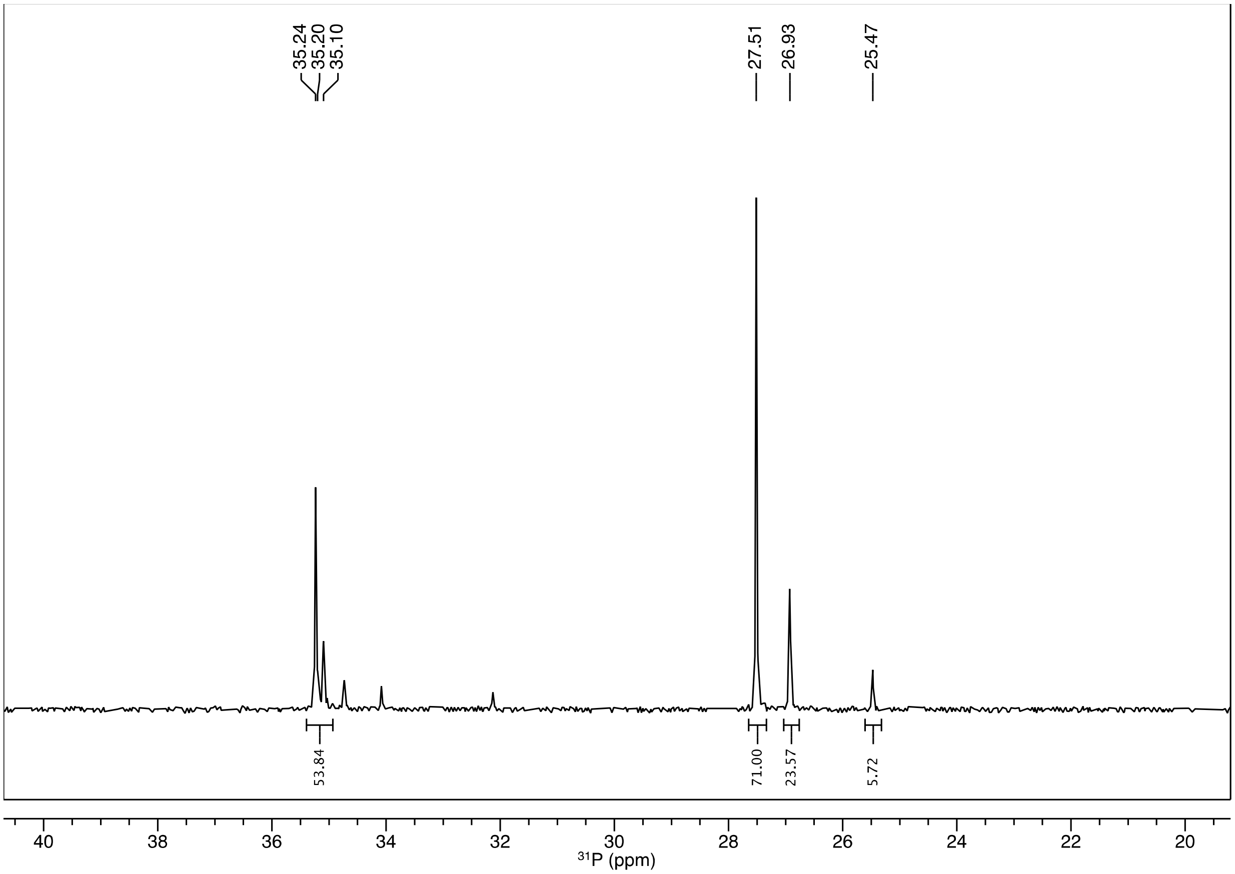

Figure S17. ^{31}P NMR spectrum (162 MHz, C_6D_6, 298K) of the reaction of **1** and 1 equivalent of 4-fluoronitrosobenzene at -135°C.

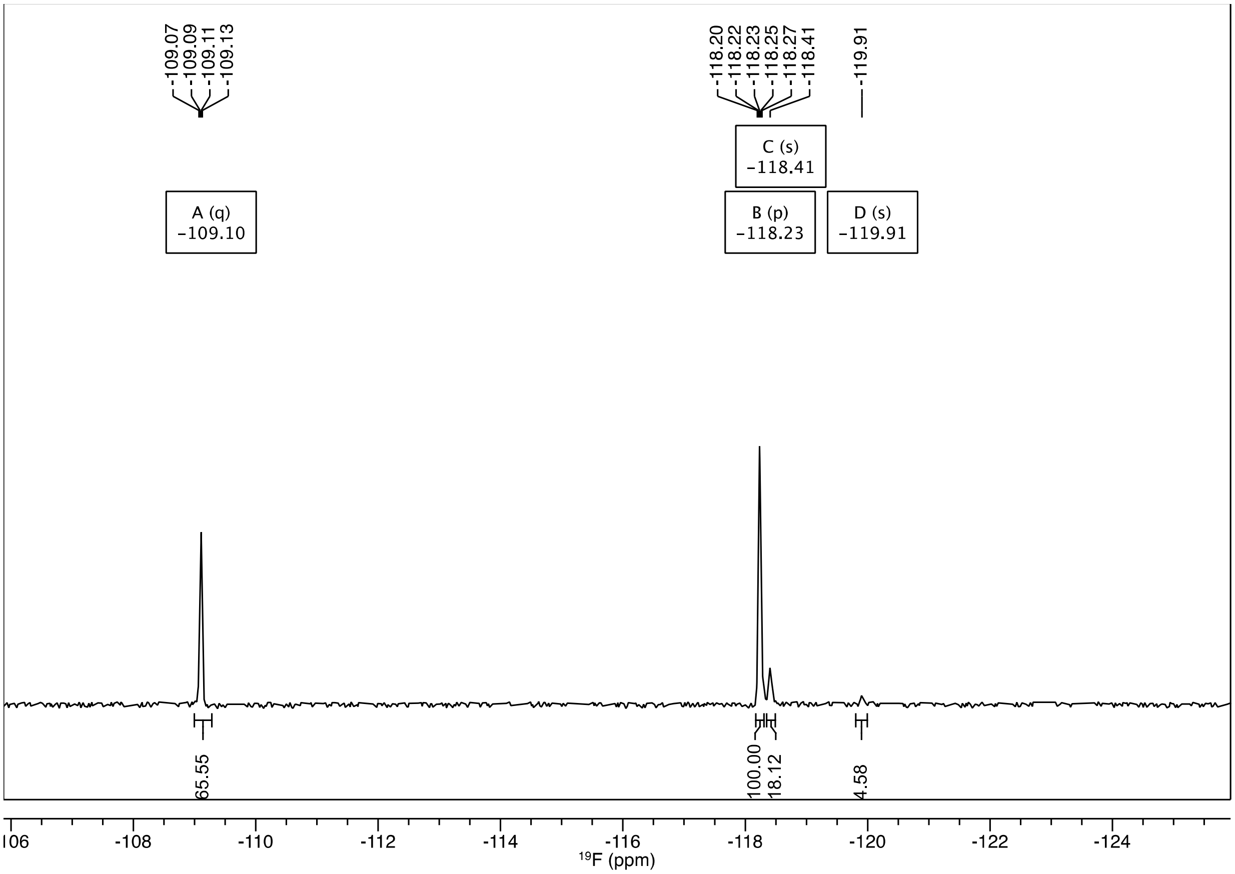

Figure S18. ^{19}F NMR spectrum (373 MHz, C_6D_6, 298K) of the reaction of **1** and 1 equivalent of 4-fluoronitrosobenzene at -135°C.

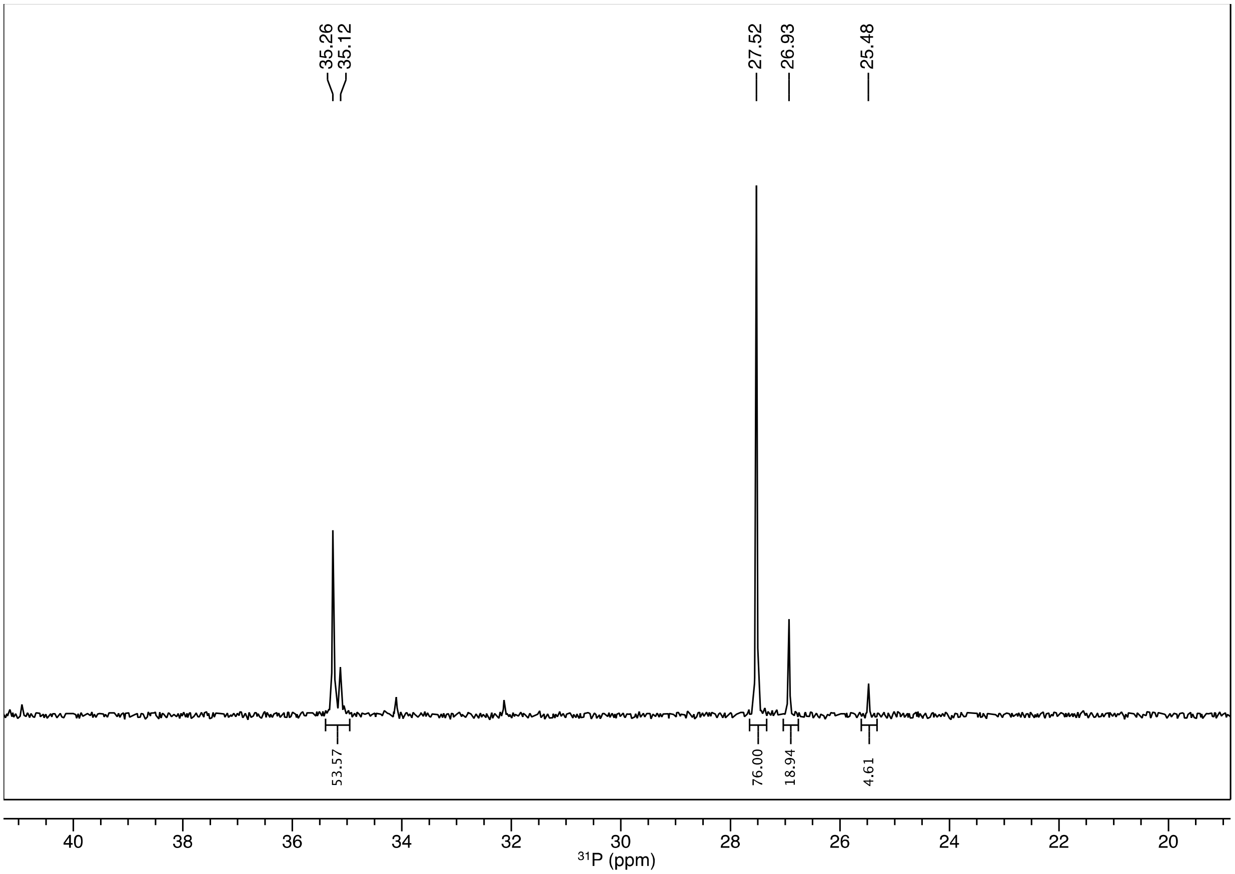

Figure S19. ^{31}P NMR spectrum (162 MHz, C_6D_6, 298K) demonstrating that additional PhNO added to the reaction mixture between **1** and 4-fluoronitrosobenzene does not appreciably consume compound **4**.

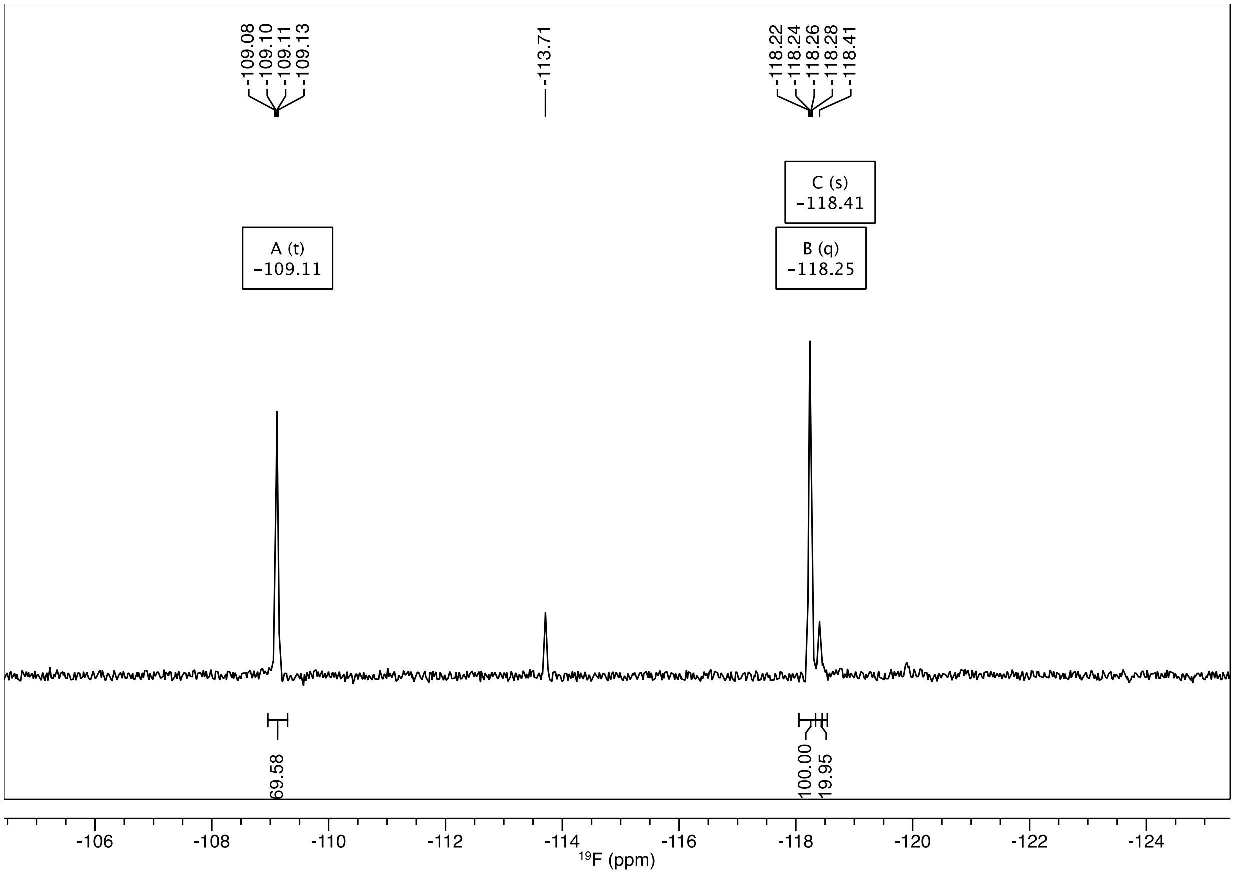

Figure S20. ^{19}F NMR spectrum (373 MHz, C_6D_6, 298K) demonstrating that additional PhNO added to the reaction mixture between **1** and 4-fluoronitrosobenzene does not appreciably consume compound **4** nor does it elicit the formation of additional 4-fluoroazobenzene.

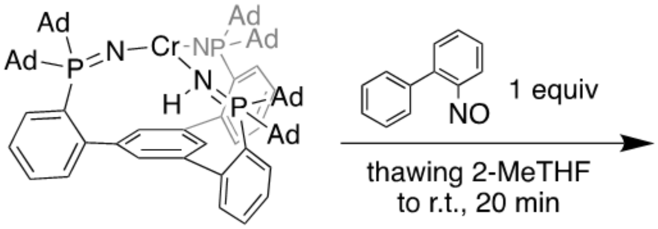

A

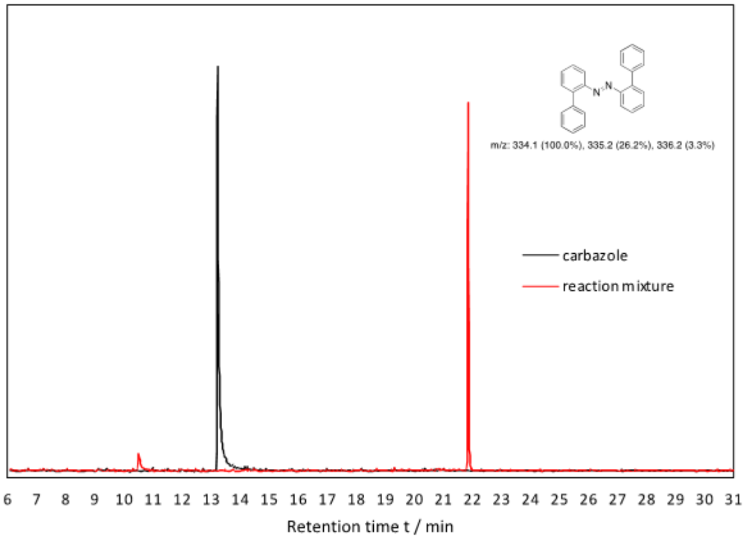

B

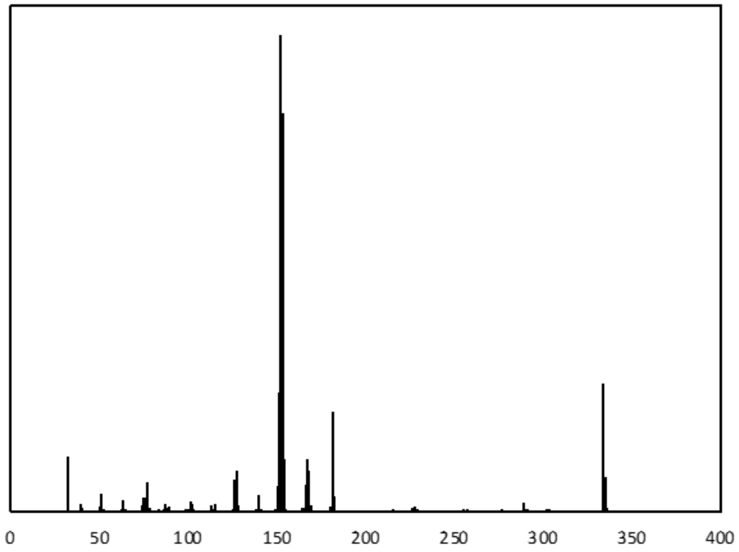

Figure S21. The reaction of **1** with 2-phenylnitrosobenzene. (A) GC-MS chromatogram of the reaction mixture following passage through an alumina plug to remove inorganic materials. The reaction mixture (red trace) indicates substantial formation of (Biphenyl)$_2$N$_2$ (retention time: 22 min, MS spectrum in (B)). The black trace represents an authentic carbazole standard.

Additional Supplementary Figures

Infrared Spectra

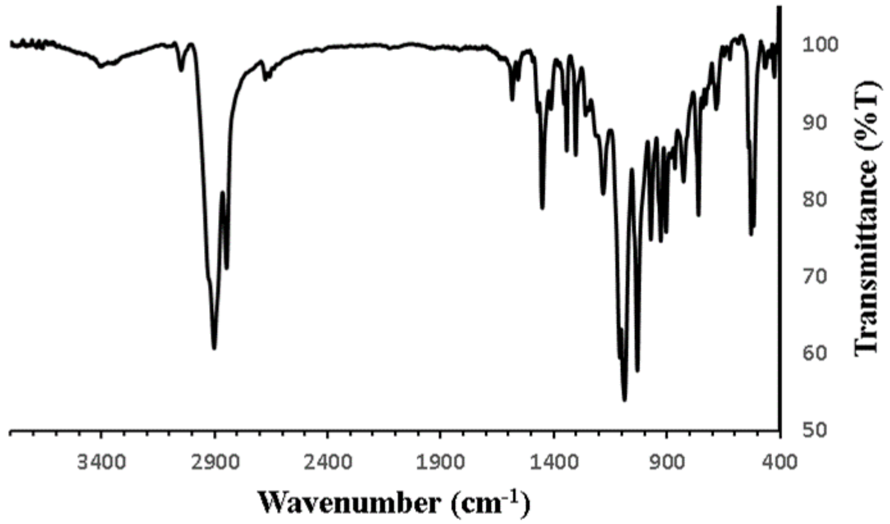

Figure S22. KBr-IR spectrum of **1**.

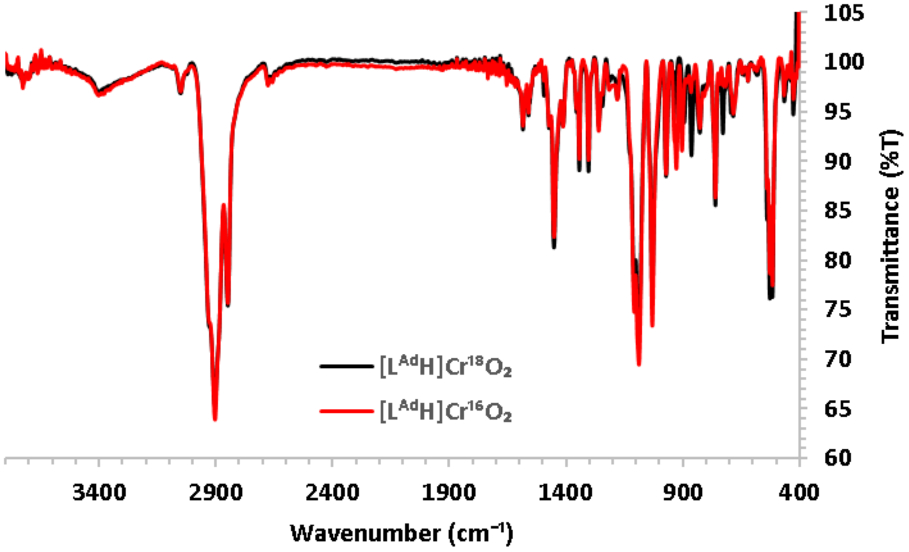

Figure S23. Full overlaid KBr-IR spectra of **2** and ^{18}**O-2.**

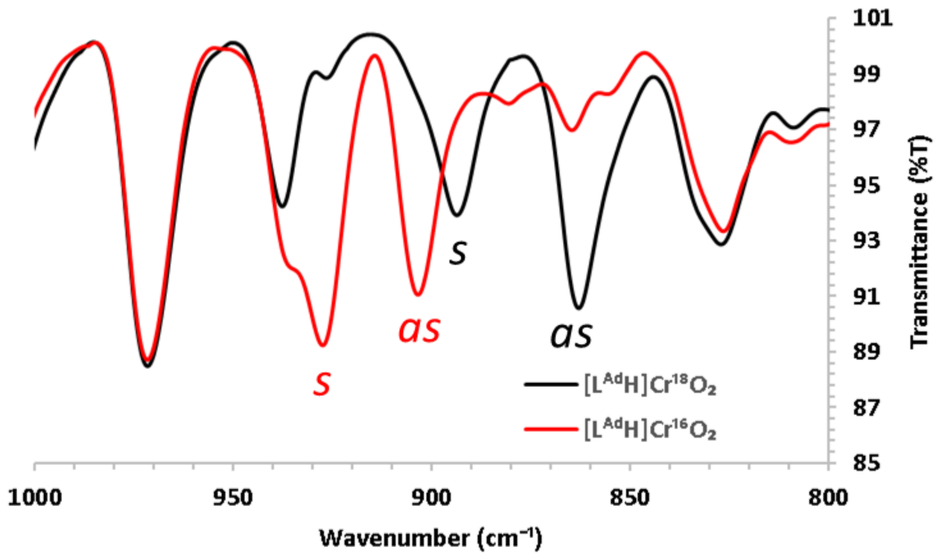

Figure S24. Zoomed in overlaid KBr-IR spectra of **2** and **^{18}O-2.** Symmetric (s) and antisymmetric (as) [O=Cr=O] stretching frequencies are noted.

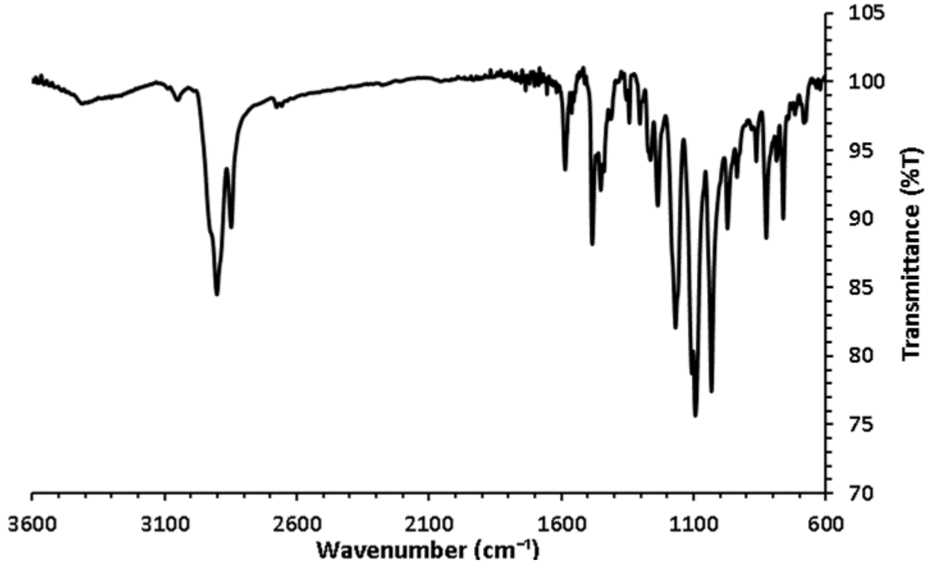

Figure S25. KBr-IR spectrum of **3.**

UV-Visible Spectra

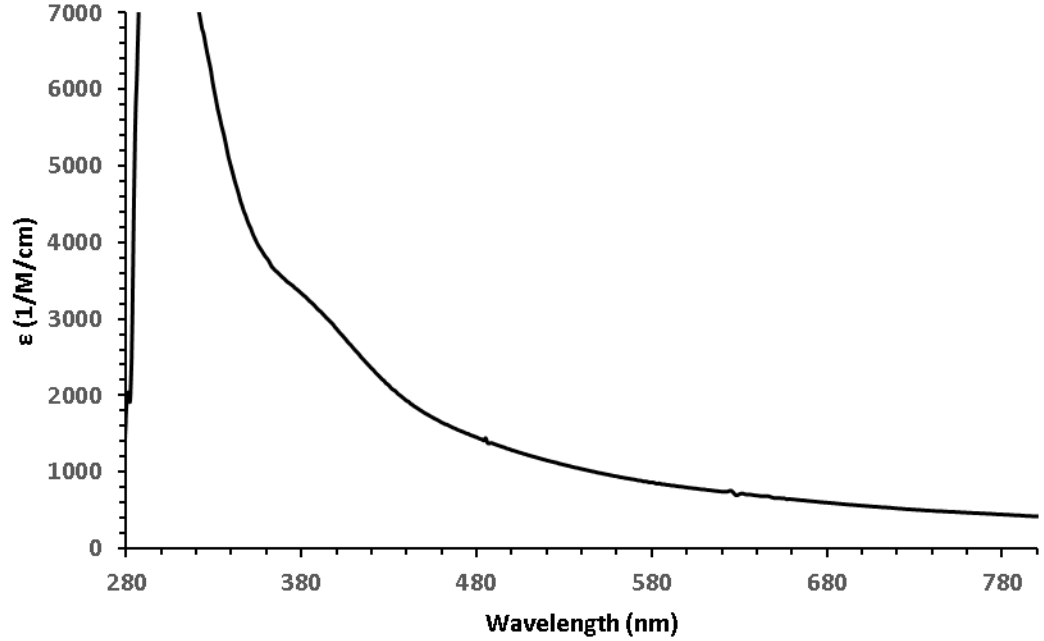

Figure S26. UV/Visible absorption spectrum of **1** in Toluene at 298 K.

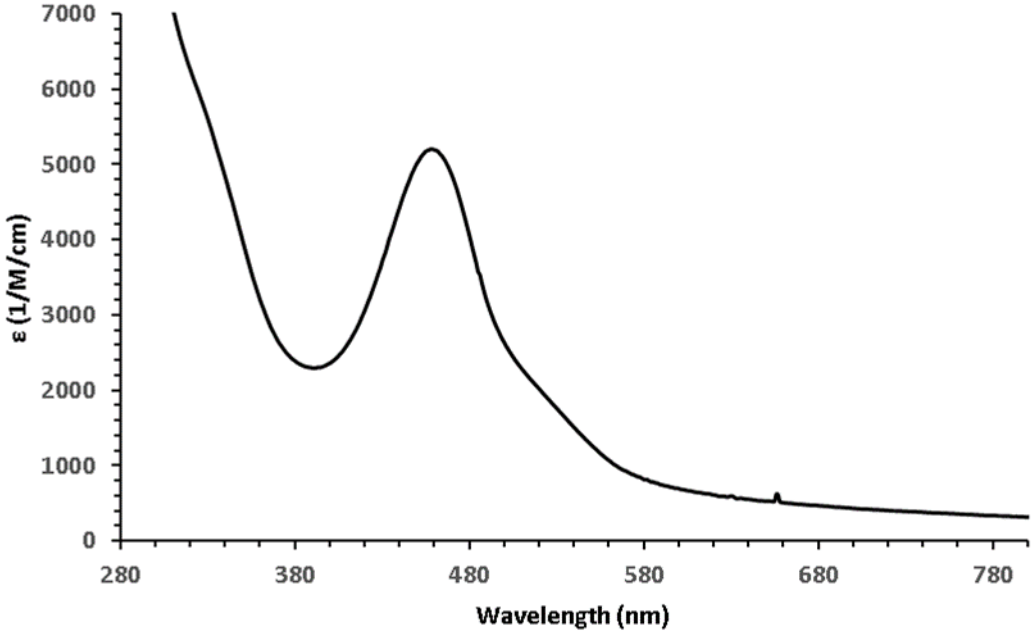

Figure S27. UV/Visible absorption spectrum of **2** in Toluene at 213 K.

NMR Spectra

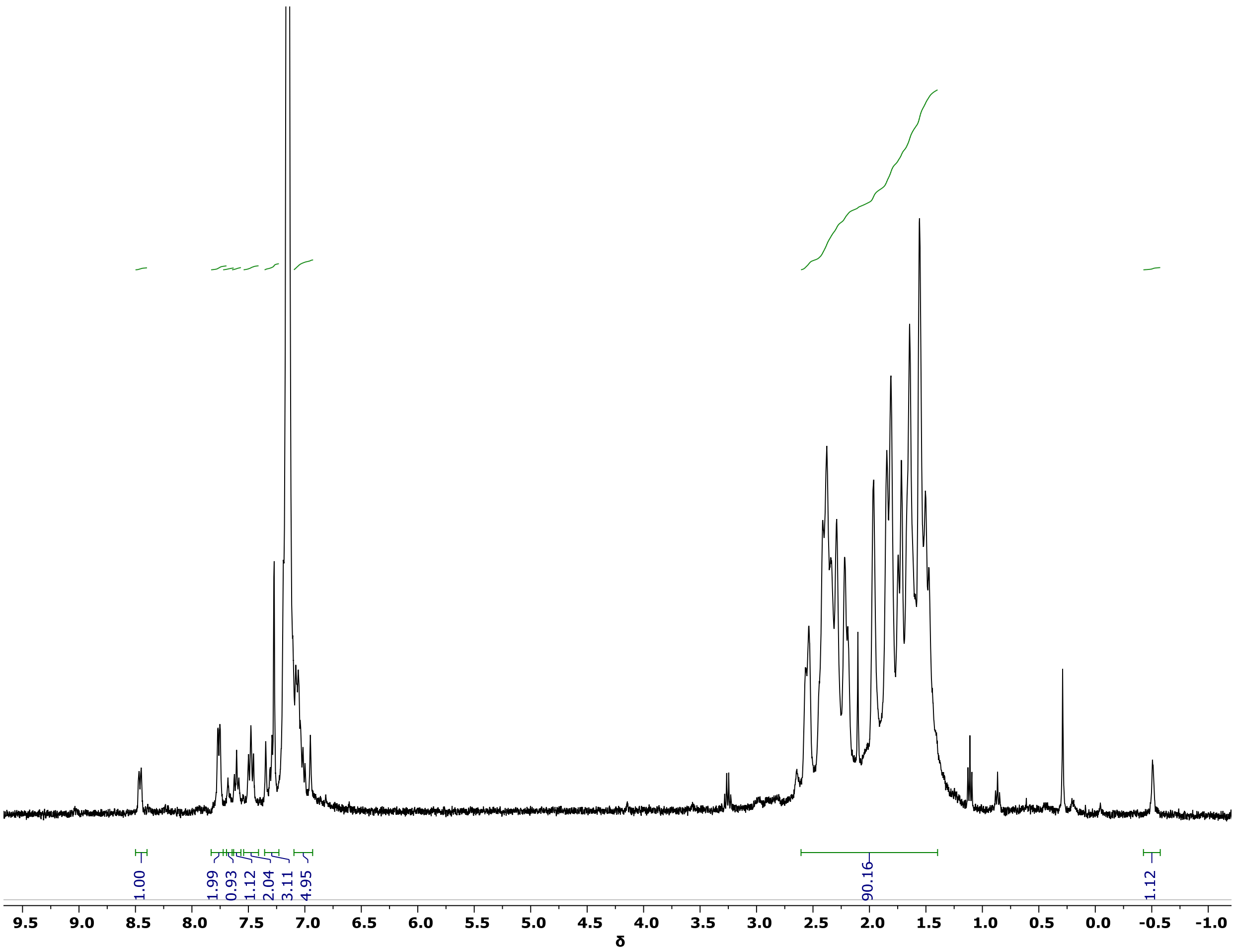

Figure S28. ^{1}H NMR spectrum (400 MHz, 298K, C_6D_6) of **2**.

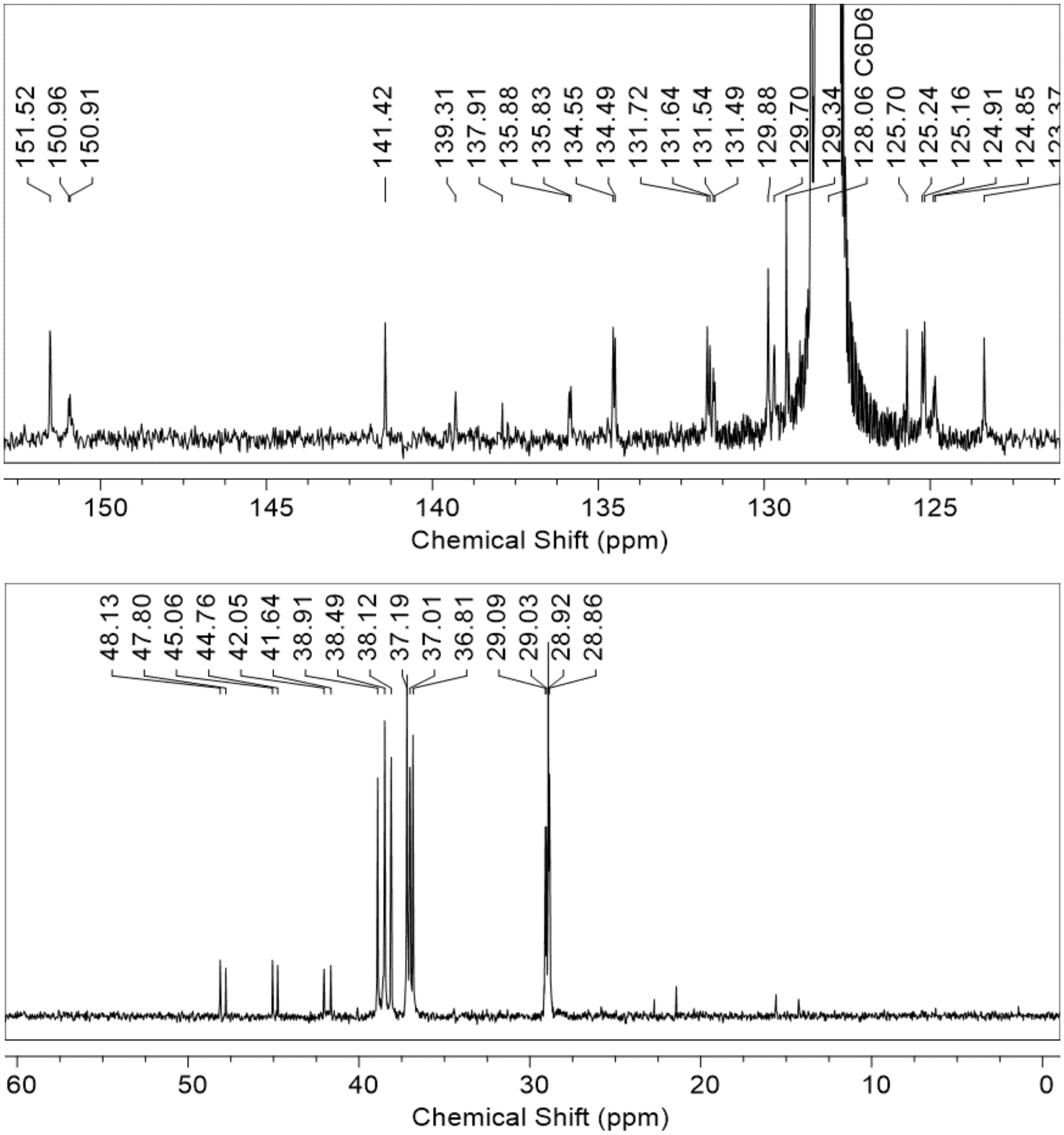

Figure S29. (Top) Aromatic and (bottom) aliphatic regions of the $^{13}C\{^1H\}$ NMR spectrum (151 MHz, 298K, C_6D_6) of **2**.

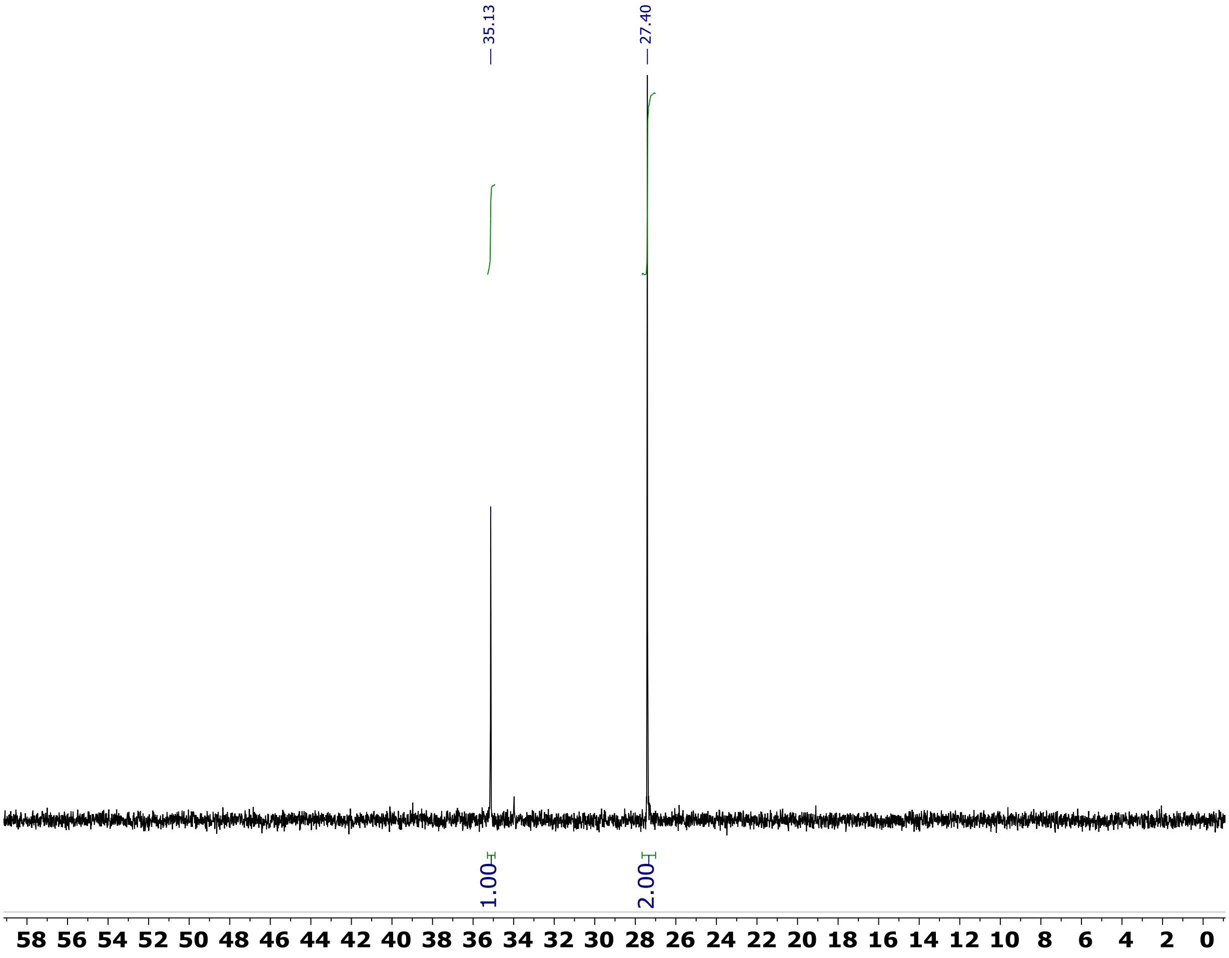

Figure S30. $^{31}P\{^1H\}$ NMR spectrum (243 MHz, 298K, C_6D_6) of **2**.

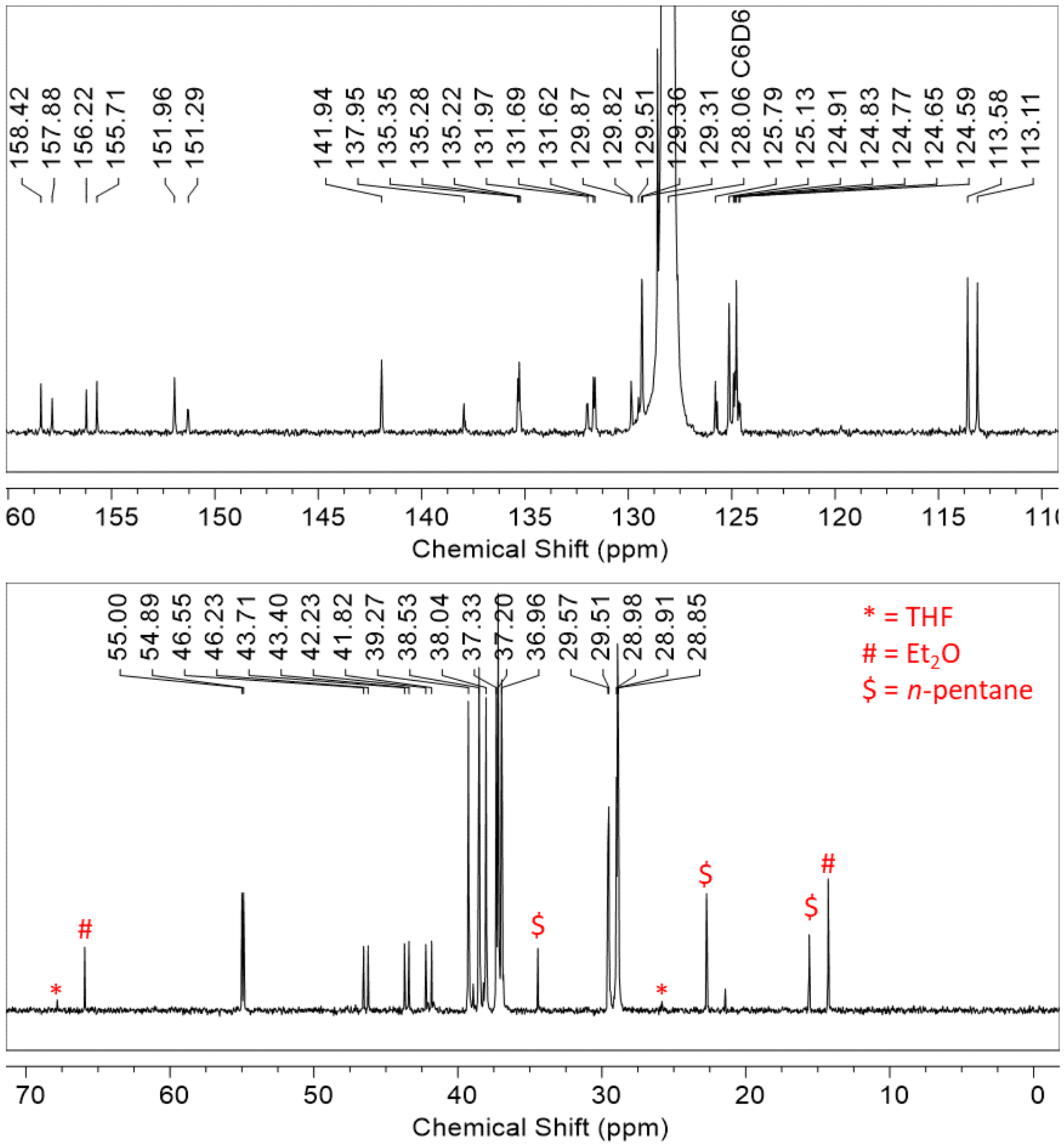

Figure S31. (Top) Aromatic and (bottom) aliphatic regions of the $^{13}C\{^{1}H\}$ NMR spectrum (151 MHz, 298K, C_6D_6) of **3**.

DFT-optimized Molecular Coordinates

($H^{tBu}L$)Cr (S = 2, gas-phase)

P	-1.83506900	2.79927900	-0.64569000
N	-1.13189800	1.42110800	-0.87660400
C	-1.86723200	3.37614800	1.14575100
C	-1.34813900	2.56687000	2.19379900
C	-1.42899600	3.03368100	3.52615600
H	-1.02647200	2.39781400	4.32338100
C	-1.99373800	4.27655900	3.84244800
H	-2.04062900	4.60932000	4.88569700
C	-2.48164500	5.08996700	2.81061100
H	-2.91123400	6.07409900	3.03175300
C	-2.41439300	4.63640900	1.48554300
H	-2.79122100	5.28887600	0.69249000
C	-0.65002200	1.24908500	2.00611500
C	-1.35530400	0.03000900	2.05739000
H	-2.44787500	0.04112300	2.13344000
P	-1.93018000	-2.83901600	-0.62909600
C	-2.01502400	-3.28345800	1.17682900
C	-1.41064300	-2.49376300	2.20169300
C	-1.49163400	-2.94801300	3.53757200
H	-1.02718400	-2.33177500	4.31543800
C	-2.14097600	-4.14095700	3.88638200
H	-2.18340400	-4.45734700	4.93442600
C	-2.73237300	-4.91927400	2.88007200
H	-3.24496600	-5.85607100	3.12643400
C	-2.66389800	-4.49109300	1.54979300
H	-3.12923500	-5.11451100	0.78098400
C	-0.67135800	-1.19949300	2.00582300
C	0.73803200	-1.20238200	1.94636100
H	1.27973400	-2.15360500	1.95228400
P	3.53219600	-0.04001700	-0.75237000
N	2.00020700	-0.11907000	-1.13194000
C	3.95611500	-0.07139700	1.09145700
C	2.96186400	-0.01769100	2.10839100
C	3.37456800	-0.00451600	3.46325100
H	2.59909500	0.04064900	4.23652000
C	4.72335900	-0.05311400	3.83327000
H	5.00341400	-0.04114200	4.89284200
C	5.70453000	-0.12336800	2.83516300
H	6.76772300	-0.17146800	3.09782000
C	5.31386500	-0.12983700	1.48967600
H	6.09522500	-0.18373600	0.72659800
C	1.46718300	0.00677700	1.91901200
C	0.76034900	1.22360000	1.97165100
H	1.31209000	2.16975500	1.97934000
C	4.25454100	1.65380300	-1.34742400
C	4.07563100	1.71057800	-2.88074500
C	3.33549800	2.71164500	-0.69925900
C	5.71358900	1.97492300	-0.96788900

H	4.77492900	1.03634300	-3.40681400
H	3.04555700	1.43551300	-3.16415100
H	4.27559300	2.73973500	-3.23763400
H	3.44762600	2.72730700	0.40036000
H	3.59914300	3.71699200	-1.08115500
H	2.27979800	2.50491200	-0.93826400
H	5.96944900	2.97527600	-1.36962300
H	5.85387000	2.01695100	0.12533100
H	6.44070700	1.26047600	-1.38786200
C	4.49286700	-1.55901300	-1.50620700
C	4.37066200	-2.73613400	-0.51129600
C	3.74467300	-1.91968200	-2.81040900
C	5.98312900	-1.34167300	-1.84846700
H	4.97401500	-2.57653600	0.39808800
H	3.32441600	-2.89651800	-0.19998600
H	4.72705800	-3.66568600	-0.99748300
H	3.85744400	-1.13607700	-3.57918200
H	4.16295600	-2.86084000	-3.21914000
H	2.66652700	-2.04749300	-2.62767700
H	6.38498900	-2.27608000	-2.28889500
H	6.13189800	-0.54295700	-2.59485000
H	6.60575200	-1.11276500	-0.96694800
C	-0.63789500	-4.01169300	-1.38623600
C	-0.52612500	-3.70537400	-2.89688300
C	0.69963400	-3.66884400	-0.69169600
C	-0.92374900	-5.51119000	-1.15551200
H	-1.43582600	-4.00055400	-3.44928200
H	-0.33962300	-2.63186600	-3.07036700
H	0.32000700	-4.28016400	-3.31908800
H	0.67289200	-3.93591900	0.37934900
H	1.50321200	-4.26165700	-1.16847200
H	0.95881600	-2.59853700	-0.78372000
H	-0.07575800	-6.08910000	-1.56961700
H	-0.99386100	-5.75644700	-0.08224200
H	-1.83741200	-5.86422300	-1.65916200
C	-3.72261300	-3.06163700	-1.34972000
C	-4.73269400	-2.55839200	-0.29115900
C	-3.81893500	-2.15582500	-2.60172400
C	-4.10054200	-4.49775300	-1.77862500
H	-4.77996300	-3.21422700	0.59348100
H	-4.49298900	-1.53772700	0.05546500
H	-5.74047400	-2.52445700	-0.74733500
H	-3.09999500	-2.44522100	-3.38565200
H	-4.83855200	-2.24912100	-3.02268400
H	-3.64243800	-1.09499700	-2.36759400
H	-3.47669800	-4.86138400	-2.61288800
H	-4.04942600	-5.23355300	-0.95959100
H	-5.14795000	-4.48867000	-2.13748500
C	-3.73407900	2.69703600	-1.10137600
C	-3.80845900	2.28925800	-2.58825300
C	-4.29881400	1.55587900	-0.22788600
C	-4.61223000	3.94149700	-0.84977200

H	-3.52405100	3.12115000	-3.25762200
H	-3.13490900	1.43911400	-2.79579000
H	-4.84400200	1.99351600	-2.84962800
H	-4.27716700	1.81957600	0.84516200
H	-5.35088900	1.35138500	-0.50857300
H	-3.71380000	0.62965900	-0.35718700
H	-5.64630200	3.72010300	-1.18368200
H	-4.66686400	4.19392200	0.22257800
H	-4.27830500	4.83324500	-1.40280800
C	-0.96646700	4.26088000	-1.62728200
C	0.18097900	4.78666600	-0.73627700
C	-0.36508800	3.59547200	-2.88591100
C	-1.83671400	5.45489100	-2.07057400
H	-0.19392800	5.37782300	0.11662600
H	0.79315100	3.96205200	-0.33388700
H	0.84591700	5.43868300	-1.33657900
H	-1.14871500	3.28881000	-3.60167000
H	0.30211700	4.31507900	-3.39984600
H	0.20975800	2.69265500	-2.61968000
H	-1.19837100	6.18231500	-2.61205000
H	-2.64168700	5.15471600	-2.76313400
H	-2.28886900	5.99948200	-1.22326600
N	-1.42451300	-1.33867000	-0.97261700
H	-2.14122100	-0.61695700	-0.78700200
Cr	0.14161900	0.00488900	-1.06103900

(H^{tBu}L)Cr (*S*=2, THF solvent)

Cr	0.14918000	0.08616500	-1.01838500
P	3.53992800	-0.06100300	-0.67128800
P	-1.88202800	-2.69853700	-0.64645100
P	-1.81731300	2.86295400	-0.56534700
N	2.03586000	0.25711200	-1.04506400
N	-1.55093500	1.27046500	-0.81549200
N	-0.84062800	-1.54825600	-0.88836200
C	-1.20965000	3.57767200	1.05861100
C	3.83558500	-0.77337500	1.06171400
C	1.38837900	-0.46154300	1.95487800
C	-0.84138000	3.74797900	-1.96183000
C	-3.48927100	-2.51177800	-1.78566700
C	0.34647400	-1.40457700	2.04786400
C	2.81250100	-0.92762900	2.04736800
C	-0.28685900	1.33916200	2.02763500
C	-2.73610400	-2.11968000	3.45568700
C	-2.56749800	-2.83393900	1.11618700
C	-1.00533400	-1.00255800	2.08530300
C	5.13016600	-1.25737600	1.37247700
C	-0.56163200	2.81261000	2.07679600
C	4.63553400	1.53950600	-0.71218700
C	-3.62794800	-3.73598400	1.38248200
C	-1.34856100	4.97538300	1.25325400
C	1.05208500	0.91176500	1.93632500
C	-2.11133700	-2.01211700	2.19059600
C	-4.23343900	-3.83667900	2.64446300
C	4.24118000	-1.43760100	-1.88265600
C	-1.31081900	0.37186700	2.13284700
C	-3.71652200	3.16117100	-0.57488400
C	-1.06182500	-4.43992200	-0.94540300
C	-3.78600900	-3.01905000	3.69183500
C	5.43614100	-1.88326400	2.58918600
C	3.13676700	-1.58097900	3.26446200
C	-0.08478300	3.48832900	3.22702400
C	4.42378200	-2.05428000	3.54382900
C	-0.24121700	4.86814700	3.40303100
C	-0.88024800	5.61963700	2.40614900
H	-2.18823900	0.69036100	-0.25140100
C	3.54283500	-1.19292600	-3.24051100
H	2.44574100	-1.18782700	-3.13099700
H	3.84310200	-0.23330900	-3.69700000
H	3.82609000	-2.00288100	-3.94158500
C	5.76429200	-1.46984500	-2.12915900
H	6.13297300	-0.54323400	-2.60208700
H	6.35575300	-1.65528800	-1.21679300
H	5.99023500	-2.30005400	-2.82887800
C	3.77912900	-2.80761200	-1.34051000
H	2.69674300	-2.80836500	-1.12216900
H	3.96763000	-3.58797200	-2.10443300
H	4.31525000	-3.10130200	-0.42173900

C	-0.60615000	-4.49542900	-2.41997100
H	-1.45807500	-4.57693300	-3.11844900
H	-0.01940300	-3.60132600	-2.69392200
H	0.03647300	-5.38474900	-2.57738500
C	0.18632400	-4.46719200	-0.03806300
H	-0.08937500	-4.47931000	1.03262900
H	0.77484900	-5.38389800	-0.24148500
H	0.83036300	-3.59143300	-0.21945200
C	-1.91022900	-5.68581800	-0.61420400
H	-2.83895300	-5.75590900	-1.20286800
H	-1.31190700	-6.59161200	-0.84159200
H	-2.17096500	-5.73436900	0.45688600
C	-4.25351800	-3.78935100	-2.18992400
H	-3.63116500	-4.48893200	-2.77367800
H	-4.67940200	-4.33678200	-1.33138800
H	-5.10870200	-3.50627600	-2.83838600
C	-2.99164400	-1.81840200	-3.07395800
H	-2.44592800	-0.88782700	-2.84373600
H	-2.31631200	-2.46473300	-3.66240500
H	-3.86071600	-1.56908800	-3.71554700
C	-4.46157800	-1.55557200	-1.06533200
H	-3.94348400	-0.64366400	-0.71853700
H	-5.26111900	-1.23873800	-1.76450200
H	-4.94646900	-2.02371400	-0.19139900
C	-4.26583700	2.65608300	-1.92728600
H	-3.95865800	3.30413000	-2.76660800
H	-3.93811300	1.62589200	-2.14361500
H	-5.37198400	2.66381500	-1.88876300
C	-4.17125800	4.61895900	-0.34359100
H	-5.27529300	4.64619400	-0.42310700
H	-3.91281500	4.98047700	0.66544500
H	-3.77343900	5.32757200	-1.08774200
C	-4.30267200	2.30611900	0.57504700
H	-4.08995700	1.22964200	0.46253500
H	-3.93466100	2.63741500	1.56235200
H	-5.40331800	2.42198600	0.57283900
C	3.97163100	2.54772400	0.25180000
H	2.92227900	2.75161500	-0.01623500
H	4.00216300	2.18708300	1.29618500
H	4.52639800	3.50639600	0.21652800
C	4.58584000	2.10637900	-2.14900200
H	3.54909900	2.19743900	-2.51656400
H	5.04258800	3.11591100	-2.16168700
H	5.15114800	1.48049000	-2.86187100
C	6.10756400	1.38588500	-0.27269800
H	6.19568400	1.10498100	0.79048200
H	6.67331800	0.65757200	-0.87500100
H	6.61100200	2.36675800	-0.38964600
C	-0.85586800	2.82819700	-3.20441000
H	-0.41637000	1.84097700	-2.97296600
H	-1.87144100	2.66718400	-3.60367400
H	-0.24830300	3.30657000	-3.99624900

C	0.62040900	3.90388000	-1.48612600
H	1.05544400	2.92563600	-1.21212100
H	1.21979700	4.31817500	-2.31924400
H	0.71516600	4.58826900	-0.62615200
C	-1.40989100	5.12758400	-2.35456800
H	-0.76450300	5.54971200	-3.14848000
H	-2.43147000	5.05978800	-2.76471000
H	-1.40804800	5.85084300	-1.52163100
H	-2.35545600	0.69116700	2.21990800
H	0.58822500	-2.47176900	2.05372600
H	1.84807400	1.65873700	1.86851900
H	-1.81800600	5.58762800	0.47932000
H	-1.00845200	6.70233400	2.51552100
H	-2.37350900	-1.48111000	4.27018200
H	-4.24585800	-3.07911100	4.68501200
H	-5.05376100	-4.54698100	2.80201400
H	-4.00508900	-4.37301100	0.57796900
H	5.92818900	-1.16319300	0.63230500
H	6.45418300	-2.24223500	2.77991000
H	4.63167600	-2.54744800	4.50043700
H	2.34509700	-1.69665100	4.01412500
H	0.41372700	2.89521900	4.00215700
H	0.13585000	5.35128800	4.31142900

($H^{tBu}L$)Cr(O_2) (^{A}2, *S* = 0, gas-phase)

P	3.34601500	0.20952200	-0.41797300
P	-1.53457000	-2.85221300	-0.47687000
P	-1.85048100	2.76888300	-0.58232800
N	1.78139200	0.41208600	-0.71115000
N	-1.28153000	1.26393200	-0.78991900
N	-0.82145100	-1.42871300	-0.56051400
C	-1.57897900	3.53404900	1.12029600
C	3.66363900	-0.49045000	1.30207300
C	1.21780100	-0.26440200	2.21612500
C	-0.91036800	3.85182200	-1.84830400
C	-3.01879800	-2.91450500	-1.73777500
C	0.25749300	-1.28686600	2.26871200
C	2.66631600	-0.64128400	2.31351400
C	-0.59256600	1.38383500	2.20455400
C	-2.87368200	-2.25590000	3.46892900
C	-2.30798300	-3.05506200	1.22497500
C	-1.12032000	-1.00126200	2.25006800
C	4.97852400	-0.91919600	1.60643100
C	-1.00755500	2.82522300	2.21947600
C	4.23530000	1.93369500	-0.35573700
C	-3.22775900	-4.10976800	1.43425900
C	-1.91639900	4.89670200	1.31028600
C	0.77737600	1.07265800	2.20386700
C	-2.11501900	-2.11812500	2.28449100
C	-3.95808700	-4.24361500	2.62365400
C	4.25497300	-0.96139400	-1.70598600
C	-1.53945700	0.34043600	2.26794200
C	-3.77286500	2.79271000	-0.79235100
C	-0.38424800	-4.40145500	-0.69654700
C	-3.79027900	-3.29995700	3.64593300
C	5.32581500	-1.49224900	2.83679600
C	3.03569700	-1.23122000	3.54754000
C	-0.78484300	3.51815800	3.43299200
C	4.34119000	-1.65484200	3.81884000
C	-1.11924400	4.86734100	3.59453000
C	-1.69590400	5.56242100	2.52350400
H	-1.75258300	0.59059100	-0.16686300
C	3.73697500	-0.56033000	-3.10859400
H	2.63470300	-0.60105300	-3.16548200
H	4.08808100	0.43808300	-3.41661400
H	4.14101400	-1.28896500	-3.83978000
C	5.79909200	-0.96297500	-1.76918200
H	6.21008000	0.01610500	-2.06844100
H	6.29666500	-1.28673800	-0.84018900
H	6.10089200	-1.68575300	-2.55326400
C	3.74988900	-2.38626900	-1.39146600
H	2.64878900	-2.42231600	-1.42234300
H	4.12859900	-3.08258200	-2.16464300
H	4.09149200	-2.75195200	-0.40716300
C	0.13161100	-4.39298700	-2.15345000

H	-0.63194900	-4.76244000	-2.86075400
H	0.43643500	-3.38169600	-2.47994600
H	1.00528600	-5.06896300	-2.23563800
C	0.79821800	-4.16401100	0.26761700
H	0.47933900	-4.26115500	1.32147700
H	1.57789400	-4.92830900	0.08415900
H	1.24766700	-3.16781800	0.12931900
C	-0.99731800	-5.77577800	-0.35163900
H	-1.86779500	-6.03726700	-0.97457100
H	-0.22830700	-6.55483500	-0.52593700
H	-1.29168300	-5.84176600	0.70990000
C	-3.57056900	-4.31232000	-2.09349300
H	-2.81607600	-4.94950300	-2.58454600
H	-3.98555000	-4.85894300	-1.22991800
H	-4.40101300	-4.18171400	-2.81512200
C	-2.52089800	-2.24181700	-3.03845900
H	-2.16247800	-1.21645600	-2.86336900
H	-1.69339600	-2.79208300	-3.51314100
H	-3.36404000	-2.20533400	-3.75656700
C	-4.15583200	-2.05003200	-1.15069700
H	-3.77921400	-1.04740900	-0.88658400
H	-4.94622000	-1.92370700	-1.91598700
H	-4.61746400	-2.49845500	-0.25431500
C	-4.09967800	2.24431500	-2.19996100
H	-3.88924800	2.98838000	-2.98804500
H	-3.52602900	1.32905200	-2.42341200
H	-5.17845800	2.00290000	-2.25414600
C	-4.46779800	4.15723500	-0.59184700
H	-5.54943800	4.02824500	-0.79088000
H	-4.37009800	4.52473800	0.44307900
H	-4.10213500	4.93708300	-1.27954100
C	-4.33493200	1.82542000	0.27598800
H	-3.93012300	0.80519500	0.17345100
H	-4.13059300	2.18491700	1.30026200
H	-5.43296800	1.75765200	0.15665500
C	3.45639400	2.79956100	0.65688100
H	2.38382200	2.83584800	0.41700300
H	3.57700400	2.41615400	1.68602400
H	3.85779800	3.83153500	0.63692000
C	4.14853400	2.58526200	-1.75406100
H	3.13071600	2.51875300	-2.17337000
H	4.42016300	3.65593100	-1.67476900
H	4.84935900	2.12113700	-2.46846300
C	5.70746800	1.90177200	0.11120000
H	5.80611900	1.52148900	1.14171700
H	6.36329700	1.30872900	-0.54437200
H	6.09398500	2.94002400	0.10734800
C	-0.85149700	3.09836100	-3.19802100
H	-0.38297100	2.10831900	-3.10365300
H	-1.84606300	2.96910500	-3.65455300
H	-0.24085500	3.70300500	-3.89631000
C	0.52657900	3.98280000	-1.29582300

H	0.96538900	2.98531800	-1.11993600
H	1.14954900	4.50855100	-2.04434600
H	0.56205700	4.56277300	-0.35710400
C	-1.50093000	5.25437700	-2.10909300
H	-0.84297100	5.76355000	-2.83877400
H	-2.50685300	5.21125500	-2.56000500
H	-1.53621700	5.89606400	-1.21399100
H	-2.60965400	0.57315900	2.29338100
H	0.58714200	-2.32847500	2.28989000
H	1.50568200	1.88525000	2.18020000
H	-2.36165200	5.46244100	0.48951800
H	-1.97193200	6.61808100	2.62431800
H	-2.71152500	-1.52718800	4.27175100
H	-4.35910400	-3.38038400	4.57918600
H	-4.65959600	-5.07726600	2.74224400
H	-3.39015800	-4.84405400	0.64154600
H	5.76303200	-0.80701500	0.85685100
H	6.35880100	-1.80987500	3.01855600
H	4.58392400	-2.10257500	4.78918300
H	2.25852800	-1.34176300	4.31229700
H	-0.34258400	2.96054300	4.26627000
H	-0.93433700	5.36871200	4.55123100
Cr	0.21169800	-0.20005900	-1.43198700
O	0.73861800	-1.19503100	-2.94740600
O	-0.08543600	-0.00196000	-3.18565700

(H^{tBu}L)Cr(O$_2$) (^{A}2, S = 1, gas-phase)

P	3.34633600	0.19649700	-0.41352100
P	-1.54442700	-2.85502700	-0.47850500
P	-1.83270500	2.78576600	-0.58797400
N	1.78271000	0.41979300	-0.69670000
N	-1.27851000	1.27587600	-0.79637100
N	-0.82389900	-1.43580200	-0.55436400
C	-1.56095900	3.54680900	1.11595300
C	3.65112000	-0.51954300	1.30429200
C	1.20311200	-0.26885300	2.21273800
C	-0.88064100	3.85890400	-1.85334100
C	-3.02745100	-2.91147600	-1.74245000
C	0.23678200	-1.28565500	2.26123700
C	2.64906000	-0.65740600	2.31305400
C	-0.59815000	1.38904400	2.20567500
C	-2.90897600	-2.23246300	3.45460900
C	-2.32811700	-3.05020500	1.22177000
C	-1.13987000	-0.99313400	2.24330900
C	4.95842600	-0.96802600	1.61151600
C	-1.00390000	2.83313100	2.21934600
C	4.25400400	1.91149400	-0.32873700
C	-3.25005200	-4.10207600	1.43270600
C	-1.88728900	4.91264200	1.30274700
C	0.76994500	1.07071100	2.20610600
C	-2.13978400	-2.10549200	2.27533900
C	-3.98980300	-4.22554400	2.61744000
C	4.25613400	-0.96820200	-1.70591500
C	-1.55147200	0.35086900	2.26554900
C	-3.75385900	2.82637100	-0.80291900
C	-0.39994400	-4.40477700	-0.69279600
C	-3.82896300	-3.27294600	3.63251600
C	5.29534900	-1.54573500	2.84268400
C	3.00817900	-1.25044900	3.54881700
C	-0.78346200	3.52467300	3.43402200
C	4.30694600	-1.69197800	3.82331000
C	-1.10615500	4.87707100	3.59242800
C	-1.66847600	5.57706400	2.51691800
H	-1.74123000	0.60950700	-0.16008400
C	3.75085700	-0.55035800	-3.10842700
H	2.64899400	-0.58826700	-3.17311900
H	4.10764100	0.45025300	-3.40211800
H	4.15839800	-1.27247400	-3.84425500
C	5.80070800	-0.97880900	-1.76057100
H	6.21900600	-0.00033300	-2.05177700
H	6.29228500	-1.31049900	-0.83101200
H	6.10285900	-1.69873700	-2.54714100
C	3.74076600	-2.39316400	-1.40761000
H	2.63946800	-2.42219800	-1.44341300
H	4.11826200	-3.08472400	-2.18569300
H	4.07606300	-2.77019700	-0.42535800
C	0.11335900	-4.40357900	-2.15093100

H	-0.65192300	-4.77640300	-2.85463300
H	0.41934800	-3.39407400	-2.48233700
H	0.98591700	-5.08115000	-2.23102400
C	0.78606600	-4.17052600	0.26786400
H	0.46875100	-4.25908100	1.32293700
H	1.55905300	-4.94225600	0.08717200
H	1.24372700	-3.17908300	0.12271300
C	-1.01961900	-5.77458000	-0.34216900
H	-1.89461600	-6.03190400	-0.96051600
H	-0.25603700	-6.55823500	-0.51881200
H	-1.30936100	-5.83668100	0.72088000
C	-3.59633600	-4.30471500	-2.08803400
H	-2.84909700	-4.95439700	-2.57388600
H	-4.01874600	-4.83984000	-1.22095100
H	-4.42457900	-4.16948100	-2.81145100
C	-2.52223700	-2.25457000	-3.04844400
H	-2.14271400	-1.23543400	-2.88164900
H	-1.70923500	-2.82496800	-3.52419700
H	-3.36803600	-2.20569800	-3.76259400
C	-4.15178000	-2.02777900	-1.15926500
H	-3.75999600	-1.02895800	-0.90278800
H	-4.94188600	-1.89543300	-1.92379900
H	-4.61756200	-2.46461600	-0.25930700
C	-4.08152400	2.28286000	-2.21236900
H	-3.86181600	3.02569500	-2.99907400
H	-3.51636300	1.36209300	-2.43493900
H	-5.16244200	2.05201100	-2.27004700
C	-4.43700600	4.19658200	-0.60201000
H	-5.51904600	4.07839400	-0.80542800
H	-4.33949000	4.56127700	0.43391000
H	-4.06087500	4.97406700	-1.28678600
C	-4.32675400	1.86131900	0.26177200
H	-3.92983600	0.83811800	0.15793000
H	-4.12330800	2.21699300	1.28757400
H	-5.42489700	1.80266600	0.13877700
C	3.47695700	2.77670200	0.68571100
H	2.40631900	2.82371100	0.43925700
H	3.58785300	2.38425400	1.71260300
H	3.88778100	3.80513800	0.67643500
C	4.18335500	2.57734700	-1.72107100
H	3.16740400	2.52625700	-2.14732000
H	4.46608000	3.64427500	-1.62975300
H	4.88338200	2.11258900	-2.43570800
C	5.72271600	1.86015300	0.14767200
H	5.81075300	1.46509900	1.17363200
H	6.37784800	1.26965700	-0.51091900
H	6.11864200	2.89483500	0.15980300
C	-0.81575700	3.09844900	-3.19902300
H	-0.35114900	2.10714300	-3.09901300
H	-1.80832100	2.97117400	-3.66052700
H	-0.19887800	3.69776800	-3.89630400
C	0.55303200	3.98967100	-1.29158500

H	0.98966300	2.99268200	-1.10668400
H	1.18181200	4.51069500	-2.03853900
H	0.58305600	4.57478100	-0.35584400
C	-1.46650200	5.26139200	-2.12512600
H	-0.80268200	5.76576700	-2.85286500
H	-2.46963500	5.21875300	-2.58225100
H	-1.50596300	5.90758300	-1.23334300
H	-2.62040000	0.58977300	2.28942100
H	0.56068200	-2.32906000	2.27929900
H	1.50216700	1.87965700	2.18517600
H	-2.32192800	5.48180500	0.47861600
H	-1.93462600	6.63548500	2.61509000
H	-2.75143200	-1.49811700	4.25318800
H	-4.40547100	-3.34446900	4.56179800
H	-4.69294500	-5.05753900	2.73766000
H	-3.40699100	-4.84255700	0.64469200
H	5.74547600	-0.86827900	0.86286800
H	6.32300000	-1.87930000	3.02616500
H	4.54122000	-2.14184000	4.79477800
H	2.22799900	-1.35030400	4.31198600
H	-0.35165100	2.96356900	4.27042600
H	-0.92314800	5.37726000	4.55011000
Cr	0.20800500	-0.20249600	-1.43342900
O	0.76338600	-1.20955400	-2.94170000
O	-0.07302200	-0.03159900	-3.19444100

(H^{tBu}L)Cr(O$_2$) (^{A}2, *S* = 2, gas-phase)

P	3.16991600	-1.28907100	-0.43205900
P	-2.85550700	-1.73770800	-0.49436400
P	-0.29388700	3.27337500	-0.55223300
N	1.88660600	-0.39504000	-0.73850600
N	-0.55824000	1.68620900	-0.76819200
N	-1.49399000	-0.95433500	-0.63689300
C	0.37674300	3.79348700	1.12655500
C	3.07986900	-2.15027100	1.24054000
C	0.97269000	-0.89191600	2.14274100
C	1.00845900	3.73620300	-1.87296900
C	-4.29874000	-1.02750300	-1.63348500
C	-0.36087300	-1.32577300	2.19271900
C	2.06810300	-1.91053600	2.21869400
C	0.18624900	1.42631200	2.18924100
C	-3.49319800	-0.69875000	3.54321900
C	-3.53689300	-1.58298400	1.25955700
C	-1.43044100	-0.40989000	2.21474800
C	4.04583200	-3.14616800	1.52317200
C	0.53260600	2.88594800	2.21757400
C	4.78911400	-0.21827400	-0.29957100
C	-4.84451000	-2.04908900	1.53425900
C	0.78246700	5.13837100	1.30918400
C	1.23407400	0.49318300	2.15601500
C	-2.84154000	-0.90508200	2.30498900
C	-5.47071300	-1.85301900	2.77337900
C	3.45467100	-2.71182000	-1.76592300
C	-1.14697300	0.96713200	2.26310600
C	-1.96010900	4.23534000	-0.70065000
C	-2.66388700	-3.65515600	-0.78412400
C	-4.79261500	-1.16112600	3.78565600
C	4.03228100	-3.90571100	2.70061500
C	2.06456400	-2.69644800	3.39777500
C	1.09672700	3.36842200	3.42229100
C	3.02359200	-3.68448700	3.64601800
C	1.49565200	4.70067800	3.57931800
C	1.33528800	5.59538200	2.51291800
H	-1.30217100	1.33259500	-0.14746800
C	3.16129800	-2.08059400	-3.14708300
H	2.18960000	-1.56664500	-3.16309800
H	3.94322700	-1.36620600	-3.45430100
H	3.13161100	-2.88744200	-3.90547300
C	4.83831500	-3.39180700	-1.86643600
H	5.63636900	-2.68154000	-2.14208700
H	5.14533100	-3.93135600	-0.95537500
H	4.79120300	-4.14730700	-2.67645400
C	2.37634600	-3.77975100	-1.47389600
H	1.37081600	-3.32706100	-1.43993600
H	2.37871200	-4.53347300	-2.28517000
H	2.55491300	-4.30460300	-0.51954600
C	-2.32073800	-3.87779700	-2.27302700

H	-3.19630100	-3.73342500	-2.93095700
H	-1.51689700	-3.20071800	-2.61162000
H	-1.97286500	-4.91988400	-2.41632100
C	-1.45513500	-4.07934100	0.07752100
H	-1.68559400	-3.99968700	1.15563200
H	-1.20752400	-5.13797100	-0.13350500
H	-0.56897200	-3.46004600	-0.13471700
C	-3.86544400	-4.53498300	-0.37837500
H	-4.78527300	-4.30452200	-0.93980300
H	-3.61293900	-5.59341300	-0.58930900
H	-4.08418300	-4.46179300	0.70052700
C	-5.48578600	-1.95657400	-1.96303800
H	-5.17148400	-2.86697700	-2.50005500
H	-6.06955200	-2.25744600	-1.07643600
H	-6.18327100	-1.41320800	-2.63239400
C	-3.61534800	-0.62133600	-2.95609100
H	-2.74947300	0.03417300	-2.77438100
H	-3.25033000	-1.49140800	-3.52678300
H	-4.34615600	-0.08227000	-3.59147200
C	-4.83257600	0.25240700	-0.95464200
H	-4.00428900	0.93698600	-0.70194100
H	-5.50628400	0.78458900	-1.65482100
H	-5.39783900	0.04192000	-0.03112900
C	-2.55804000	3.94447800	-2.09505600
H	-2.02291100	4.48786400	-2.89316900
H	-2.53356400	2.86693500	-2.32802800
H	-3.61166600	4.28234100	-2.11548300
C	-1.87327300	5.76027500	-0.47288200
H	-2.88166300	6.19103400	-0.62558900
H	-1.56560800	6.00649400	0.55675100
H	-1.19333100	6.26903400	-1.17559600
C	-2.89538800	3.65679200	0.38710600
H	-3.06382500	2.57417500	0.26455100
H	-2.50375800	3.84129700	1.40302900
H	-3.88141700	4.15321300	0.31223900
C	4.49055000	0.90774600	0.71384500
H	3.58249800	1.46880800	0.44654200
H	4.36522400	0.50088600	1.73365400
H	5.34546900	1.61152000	0.73983700
C	5.06841300	0.40729500	-1.68442800
H	4.15716800	0.85055200	-2.12016700
H	5.82635700	1.20863900	-1.58174100
H	5.46957700	-0.33248500	-2.39868800
C	6.05305600	-0.95171000	0.20236100
H	5.92413900	-1.33597800	1.22817400
H	6.36808200	-1.78542900	-0.44485400
H	6.89043300	-0.22606000	0.22784700
C	0.62992800	3.03220100	-3.19864500
H	0.49180800	1.94886000	-3.06615000
H	-0.28978500	3.44150400	-3.64798500
H	1.45519600	3.19243300	-3.91853300
C	2.34162400	3.14663700	-1.36166800

H	2.24341800	2.06254500	-1.16717900
H	3.11607500	3.29286900	-2.13881800
H	2.68667900	3.64853800	-0.44117300
C	1.18031300	5.24371200	-2.15923400
H	1.96459500	5.35109800	-2.93248500
H	0.26484900	5.70903200	-2.56318600
H	1.51990800	5.82014900	-1.28315500
H	-1.97226700	1.68492800	2.32680800
H	-0.57532200	-2.39773200	2.18221500
H	2.26503800	0.84977200	2.12595500
H	0.67009800	5.85388400	0.49146700
H	1.63924000	6.64361200	2.61047600
H	-2.94212500	-0.17643800	4.33419300
H	-5.26523800	-0.98925300	4.75946000
H	-6.48508800	-2.23390000	2.93943900
H	-5.40016700	-2.57403900	0.75350500
H	4.83601800	-3.34503300	0.79732600
H	4.80314700	-4.66636500	2.86894300
H	2.98511100	-4.26761900	4.57313400
H	1.28281000	-2.49900000	4.14010500
H	1.21330800	2.66081400	4.25076600
H	1.92707800	5.03646200	4.52903900
Cr	0.11569200	-0.22402000	-1.38277100
O	0.06036300	-1.43123900	-2.93751900
O	-0.48794400	-0.24710500	-3.29585200

$(H^{tBu}L)Cr(O_2)$ ($^{B}2$, S = 0, gas-phase)

P	-2.30359400	2.94338600	0.11834100
N	-2.07470700	1.40136400	0.47776600
C	-1.24880800	3.47604200	-1.32615300
C	-0.30737800	2.61396300	-1.96107100
C	0.35690200	3.07756700	-3.12033500
H	1.08174500	2.40696500	-3.59487000
C	0.12874900	4.35347700	-3.64950100
H	0.66430700	4.67867700	-4.54866100
C	-0.77810900	5.21082100	-3.01165700
H	-0.96411000	6.21841800	-3.40000100
C	-1.45444400	4.76870000	-1.86711200
H	-2.15978800	5.45162800	-1.38714700
C	0.09297600	1.25146200	-1.47497600
C	1.09269600	1.14224300	-0.49305400
H	1.51926300	2.04897200	-0.05273500
P	4.92568200	-0.02523100	-0.26795300
C	3.83863700	-0.20940200	1.21442800
C	2.41714900	-0.23992900	1.15613500
C	1.68596500	-0.40984700	2.35715700
H	0.58903600	-0.42815900	2.28522900
C	2.32239200	-0.54178000	3.59725000
H	1.72191300	-0.66540700	4.50519800
C	3.72248300	-0.51161500	3.65923200
H	4.24363200	-0.61627500	4.61773100
C	4.46046100	-0.34923400	2.47923200
H	5.55186800	-0.33602100	2.54743300
C	1.54237300	-0.11595700	-0.05906000
C	0.96251700	-1.26911800	-0.60791800
H	1.29247100	-2.25385100	-0.26221700
P	-2.32194700	-2.94292100	0.08574900
N	-1.90260000	-1.50716800	0.64950300
C	-1.44902200	-3.35744100	-1.51124100
C	-0.58064600	-2.44682000	-2.18375300
C	-0.11006800	-2.79175500	-3.47168100
H	0.55512700	-2.08408100	-3.97878900
C	-0.43347600	-4.01080200	-4.08036500
H	-0.04246500	-4.24744400	-5.07638400
C	-1.23535700	-4.93259600	-3.39302800
H	-1.47747800	-5.90455000	-3.83722900
C	-1.73829400	-4.59851500	-2.12852200
H	-2.37718900	-5.32348300	-1.61698700
C	-0.04106900	-1.18085800	-1.59050100
C	-0.44560200	0.08485800	-2.05097300
H	-1.22871300	0.16501700	-2.81304200
C	-4.19890800	-2.90743400	-0.35504700
C	-4.96961600	-2.61791600	0.95307000
C	-4.35344100	-1.70110600	-1.31115900
C	-4.77204500	-4.16158800	-1.04716000
H	-4.98913900	-3.49168100	1.62890300
H	-4.52847800	-1.75880500	1.48993400

H	-6.01898800	-2.36863700	0.70418500
H	-3.80632400	-1.85822000	-2.25807800
H	-5.42578200	-1.57189300	-1.55305900
H	-3.98064600	-0.77602000	-0.83939300
H	-5.85752300	-4.00363500	-1.19809000
H	-4.32632700	-4.32538800	-2.04221200
H	-4.65874900	-5.08277200	-0.45146200
C	-1.83102000	-4.27811000	1.38621300
C	-0.30785700	-4.49972600	1.23777600
C	-2.10614300	-3.67484900	2.78463700
C	-2.56930200	-5.62734000	1.26012500
H	-0.04152600	-4.96020500	0.27075700
H	0.24308900	-3.54886000	1.33848200
H	0.03535900	-5.17520900	2.04446200
H	-3.18148700	-3.52578900	2.97640000
H	-1.72314800	-4.37849000	3.54882400
H	-1.60476500	-2.70287900	2.91775700
H	-2.18279900	-6.31357900	2.03852000
H	-3.65513500	-5.52628300	1.42771000
H	-2.40652800	-6.12220800	0.28722900
C	5.82945800	1.67593300	-0.04088200
C	6.81787000	1.84252300	-1.21648000
C	4.70277000	2.72693500	-0.18376600
C	6.55933500	1.92168300	1.29599800
H	7.68994200	1.17156800	-1.12662600
H	6.32980400	1.65182400	-2.18969300
H	7.19973100	2.88162100	-1.23595100
H	3.99782700	2.67662500	0.66553600
H	5.14575700	3.74181700	-0.19709800
H	4.12725200	2.57229700	-1.11158100
H	7.00161900	2.93785700	1.27968800
H	5.86420300	1.88379500	2.15131800
H	7.38367100	1.21314500	1.48226200
C	6.18826900	-1.50978600	-0.22931600
C	5.37639100	-2.75832300	0.18667400
C	6.64973000	-1.70702600	-1.69355800
C	7.43259900	-1.36962600	0.66989100
H	5.08285100	-2.73020000	1.24932200
H	4.45989500	-2.85323600	-0.42189400
H	5.99172100	-3.66484700	0.02488300
H	7.24947500	-0.85868800	-2.06796100
H	7.28004800	-2.61477000	-1.75767200
H	5.78345300	-1.84630600	-2.36255000
H	8.08189700	-0.53098600	0.36408400
H	7.18110700	-1.24350200	1.73666400
H	8.03964000	-2.29377800	0.59231900
C	-1.83332200	4.03697000	1.62106500
C	-2.86956700	3.78272500	2.73989500
C	-0.45425300	3.51352800	2.08891400
C	-1.72411400	5.54591200	1.32034400
H	-3.84018200	4.26155700	2.52114900
H	-3.03260500	2.70536000	2.91713900

H	-2.48915200	4.22444200	3.68067800
H	0.31997900	3.65113700	1.31312300
H	-0.14409600	4.08938300	2.98170300
H	-0.50137200	2.44566400	2.35891700
H	-1.46735200	6.07070300	2.26073700
H	-0.92429200	5.76556000	0.59296300
H	-2.66738100	5.98375300	0.95056400
C	-4.14215500	3.15015400	-0.45322600
C	-4.22133800	2.52346500	-1.86565300
C	-5.01913600	2.31539400	0.51139000
C	-4.67548000	4.59733600	-0.49355800
H	-3.66048700	3.10815200	-2.61445200
H	-3.83128900	1.49107800	-1.86469200
H	-5.28128700	2.48895400	-2.18259500
H	-5.14647400	2.80672700	1.48961300
H	-6.02318500	2.19629800	0.06110600
H	-4.58296000	1.31635600	0.68941000
H	-5.74113600	4.56882100	-0.79347400
H	-4.63035200	5.09030900	0.49268200
H	-4.15350000	5.22997100	-1.23110400
N	3.96550600	-0.07239500	-1.55028700
H	4.48326300	0.01428600	-2.43612800
Cr	-2.13904300	-0.00504200	1.49452000
O	-3.11781800	0.08005000	3.00674800
O	-1.67940000	0.10971800	3.26543800

($H^{tBu}L$)Cr(O_2) (^{B}2, S = 1, gas-phase)

P	-2.34701100	2.96198400	0.04728400
N	-2.33333200	1.40774700	0.38731600
C	-1.03312400	3.44595300	-1.19395400
C	0.05248400	2.59840800	-1.55519600
C	1.04552500	3.10204300	-2.43023300
H	1.88329400	2.43976300	-2.67701800
C	0.97758900	4.39216600	-2.96820400
H	1.76162500	4.74649300	-3.64719800
C	-0.10082000	5.22149400	-2.62995200
H	-0.17845100	6.23501900	-3.03953900
C	-1.08108100	4.74820700	-1.74848900
H	-1.89920200	5.42020700	-1.47781100
C	0.28299200	1.18755800	-1.09735500
C	1.29476700	0.93706400	-0.15666900
H	1.81857600	1.77626800	0.31093400
P	5.00743000	-0.06199500	-0.27954800
C	4.04757200	-0.52885800	1.23367600
C	2.62919300	-0.65871300	1.28744900
C	2.03403900	-1.09417800	2.49686500
H	0.94139100	-1.17790200	2.52856900
C	2.79278000	-1.39521100	3.63424500
H	2.29343300	-1.72148700	4.55332300
C	4.18740200	-1.27068400	3.58287800
H	4.80408800	-1.50400100	4.45810600
C	4.79478200	-0.84581900	2.39417500
H	5.88507200	-0.76946200	2.36909000
C	1.65756900	-0.38052700	0.17952900
C	0.98103400	-1.44762300	-0.42862300
H	1.24876200	-2.47718500	-0.16843000
P	-2.59008000	-2.81061900	0.04327500
N	-1.94537700	-1.51178500	0.72266100
C	-1.69141800	-3.22131900	-1.53685800
C	-0.66390900	-2.39004700	-2.07048800
C	-0.11856400	-2.72098400	-3.33247600
H	0.67041400	-2.07524100	-3.73374300
C	-0.53511600	-3.84890600	-4.05066000
H	-0.08555600	-4.07604600	-5.02400200
C	-1.51227800	-4.69200800	-3.50400100
H	-1.83662600	-5.59260800	-4.03736000
C	-2.08070700	-4.37170000	-2.26368800
H	-2.84824800	-5.03474300	-1.85573100
C	-0.04160000	-1.21592100	-1.37044800
C	-0.36608000	0.10414800	-1.72258900
H	-1.14887600	0.29508800	-2.46408300
C	-4.43216000	-2.54114700	-0.48283400
C	-5.23197900	-2.23686500	0.80527000
C	-4.39512100	-1.28507300	-1.38614800
C	-5.12352500	-3.67784100	-1.26575100
H	-5.41093800	-3.14850500	1.40251200
H	-4.71992400	-1.50052700	1.45033700

H	-6.22084000	-1.82180000	0.53108500
H	-3.87857100	-1.49226300	-2.34029000
H	-5.43327800	-0.98145700	-1.62166400
H	-3.88294400	-0.43796200	-0.89758300
H	-6.17327400	-3.37938700	-1.45624500
H	-4.65278200	-3.84955800	-2.24800700
H	-5.15239100	-4.63279600	-0.71552400
C	-2.33298800	-4.28778700	1.25680900
C	-0.83825700	-4.67530000	1.16677000
C	-2.61881400	-3.75496400	2.68106300
C	-3.20928200	-5.52819500	0.99008800
H	-0.57532100	-5.10989800	0.18690300
H	-0.19072000	-3.79977000	1.34610700
H	-0.61504400	-5.42919400	1.94599600
H	-3.67851500	-3.48723100	2.82678000
H	-2.36965900	-4.54989800	3.41049100
H	-2.01266000	-2.86363400	2.90858300
H	-2.94951200	-6.30877700	1.73136600
H	-4.28490600	-5.31364000	1.10955400
H	-3.04474600	-5.96798300	-0.00867200
C	5.94848000	1.57739900	0.17107100
C	6.86441400	1.92888800	-1.02333700
C	4.83291600	2.64434700	0.27464200
C	6.76568800	1.60315000	1.47932400
H	7.73732600	1.25637500	-1.09734100
H	6.31553200	1.89460300	-1.98205700
H	7.25151900	2.95908400	-0.90151600
H	4.17187800	2.44883100	1.13822400
H	5.29063400	3.64228000	0.41856100
H	4.20873700	2.66375800	-0.63388400
H	7.24493300	2.59720600	1.58041100
H	6.12326500	1.46116900	2.36432300
H	7.57068900	0.85048100	1.50544500
C	6.25485100	-1.52202600	-0.60888400
C	5.47144000	-2.83126900	-0.35632300
C	6.60082700	-1.45449400	-2.11640400
C	7.56776200	-1.53627000	0.20039800
H	5.27417800	-2.99652500	0.71609400
H	4.50378900	-2.82413400	-0.88819300
H	6.06297200	-3.68908500	-0.73161500
H	7.15307400	-0.53684900	-2.38437700
H	7.24122200	-2.31798300	-2.38001200
H	5.68857700	-1.50829900	-2.73428400
H	8.19590100	-0.65094600	0.00095300
H	7.40094600	-1.60729000	1.28858100
H	8.16020000	-2.42669500	-0.08998000
C	-1.97528400	4.01440500	1.61544600
C	-3.12462900	3.81239800	2.62999900
C	-0.67791300	3.41452000	2.20820900
C	-1.74933700	5.51787100	1.35036200
H	-4.05716500	4.31127000	2.31106000
H	-3.33149100	2.74269700	2.81125300

H	-2.82605600	4.26321000	3.59616100
H	0.16724300	3.48390700	1.50037400
H	-0.40683300	3.98598000	3.11668400
H	-0.81749100	2.35982000	2.49717400
H	-1.54396300	6.01322800	2.31907300
H	-0.87811400	5.69687700	0.69787700
H	-2.62638900	6.01944900	0.90816000
C	-4.05904400	3.39465900	-0.76917700
C	-3.98920200	2.88761000	-2.22777800
C	-5.12474100	2.57144300	-0.00809200
C	-4.48832700	4.87717200	-0.74600000
H	-3.29041500	3.47851800	-2.84379400
H	-3.67306000	1.83031600	-2.26493600
H	-4.99417300	2.95824300	-2.68711300
H	-5.25731700	2.91646100	1.03063200
H	-6.09590900	2.68001200	-0.52856400
H	-4.85687000	1.50424800	0.02433300
H	-5.49051100	4.96028600	-1.21066600
H	-4.57497600	5.27127900	0.28077900
H	-3.81884400	5.53940000	-1.31990100
N	3.95916200	0.12141200	-1.47999900
H	4.42966500	0.30198100	-2.37808800
Cr	-2.27585700	0.03872200	1.52743700
O	-3.28780400	0.16946100	3.04042500
O	-1.81806000	0.11334200	3.27631000

($H^{tBu}L$)Cr(O_2) ($^{B}2$, *S* = 2, gas-phase)

P	-2.20748300	3.06962400	0.01144600
N	-2.23983300	1.55187400	0.46297700
C	-0.85809500	3.39653900	-1.23667300
C	0.20152800	2.48137800	-1.49286400
C	1.24244400	2.87491700	-2.36961900
H	2.05826400	2.16144500	-2.53467200
C	1.24420800	4.11898700	-3.01069000
H	2.06369400	4.38747200	-3.68733900
C	0.18728700	5.01084400	-2.78202200
H	0.16112600	5.98829900	-3.27710000
C	-0.83858900	4.64768000	-1.89986900
H	-1.63980800	5.36720200	-1.71392400
C	0.36008300	1.09409000	-0.94063200
C	1.37690700	0.84950200	-0.00210900
H	1.93542300	1.69052100	0.41994400
P	5.04428700	-0.16854100	-0.25041800
C	4.14578700	-0.59793000	1.31324600
C	2.73123200	-0.73297500	1.43776600
C	2.20304600	-1.16709100	2.67783700
H	1.11447000	-1.26568600	2.75871500
C	3.01682400	-1.45431800	3.77979600
H	2.56653100	-1.78220000	4.72351000
C	4.40592300	-1.31538600	3.66097900
H	5.06658300	-1.53532400	4.50701900
C	4.95003300	-0.89778100	2.43970600
H	6.03707200	-0.81514100	2.36002200
C	1.71109100	-0.46409800	0.37292900
C	1.00205600	-1.53676400	-0.19129600
H	1.26100300	-2.56399700	0.08777800
P	-2.79264700	-2.76455200	0.01049200
N	-2.16489300	-1.49883100	0.75002300
C	-1.75347200	-3.24167000	-1.46162300
C	-0.61073500	-2.49120000	-1.85845300
C	0.09642000	-2.90082400	-3.01361100
H	0.97613600	-2.31743800	-3.30729700
C	-0.28471300	-4.02103000	-3.76233300
H	0.28919500	-4.30843700	-4.65074700
C	-1.39472000	-4.77467100	-3.35692000
H	-1.70290000	-5.66431600	-3.91780900
C	-2.11364600	-4.38083600	-2.22033100
H	-2.97618500	-4.97893300	-1.91556700
C	-0.02073500	-1.31189000	-1.13489100
C	-0.32276200	0.00518600	-1.51854400
H	-1.09608200	0.19111600	-2.27077800
C	-4.55026800	-2.39421500	-0.72482900
C	-5.47539800	-2.05094700	0.46291300
C	-4.33070500	-1.12429600	-1.58119200
C	-5.20898400	-3.47580100	-1.60592100
H	-5.75772100	-2.94568000	1.04597800
H	-4.99541500	-1.32800800	1.14795000

H	-6.40880800	-1.59272800	0.08351800
H	-3.70393000	-1.33654100	-2.46630500
H	-5.30914200	-0.74951500	-1.93913900
H	-3.83599200	-0.32794400	-0.99692500
H	-6.20887200	-3.11424900	-1.91809200
H	-4.63201200	-3.66473400	-2.52636600
H	-5.36018600	-4.43366900	-1.08054100
C	-2.75487200	-4.27605300	1.21468200
C	-1.28073500	-4.73934400	1.27919500
C	-3.16718600	-3.75077800	2.60899400
C	-3.65813900	-5.46788900	0.83740900
H	-0.94643400	-5.19368800	0.33100900
H	-0.61075200	-3.89456400	1.51577200
H	-1.17388600	-5.49740100	2.07902600
H	-4.22333000	-3.43421200	2.64400300
H	-3.03444800	-4.56239100	3.35032500
H	-2.54668700	-2.89202100	2.90998000
H	-3.52251800	-6.26825000	1.59120800
H	-4.72878900	-5.20035700	0.83954800
H	-3.40616800	-5.90633500	-0.14316600
C	6.01830400	1.47015600	0.13115000
C	6.88131200	1.79122600	-1.11047900
C	4.91756500	2.54852400	0.26681600
C	6.89747200	1.51321900	1.39830500
H	7.74571200	1.11081800	-1.20825000
H	6.29015700	1.74113000	-2.04300400
H	7.27984200	2.82086400	-1.02743100
H	4.29642500	2.37524700	1.16412600
H	5.38986200	3.54471900	0.36999600
H	4.25125100	2.55746000	-0.61130100
H	7.39012300	2.50423300	1.45606100
H	6.29783100	1.39573300	2.31626000
H	7.69609600	0.75346700	1.40027600
C	6.26241900	-1.64957600	-0.60113900
C	5.47782700	-2.94448800	-0.28625500
C	6.54353400	-1.61826200	-2.12325300
C	7.60955900	-1.66024500	0.14985200
H	5.33042200	-3.08742800	0.79725400
H	4.48592700	-2.93568900	-0.77132300
H	6.04126900	-3.81609600	-0.67265500
H	7.08828600	-0.71045400	-2.43655000
H	7.16756200	-2.49147300	-2.39366500
H	5.60541100	-1.68149300	-2.69997100
H	8.23661000	-0.78577900	-0.09589900
H	7.49100400	-1.70750700	1.24564400
H	8.18035900	-2.56238100	-0.14771600
C	-1.80376500	4.25124900	1.48961500
C	-2.99842300	4.26653500	2.46943700
C	-0.59000000	3.59550100	2.18827100
C	-1.43090100	5.69731500	1.10072300
H	-3.86039600	4.83026900	2.07124100
H	-3.33191500	3.24827400	2.72875300

H	-2.68788600	4.76583700	3.40798500
H	0.29077400	3.55501800	1.52268400
H	-0.31670700	4.19316700	3.07941100
H	-0.82325700	2.56740100	2.50938500
H	-1.21405300	6.26550300	2.02679100
H	-0.52758100	5.73707000	0.46961900
H	-2.24435300	6.22839800	0.57795500
C	-3.89093700	3.54779600	-0.85414200
C	-3.83498900	2.94764000	-2.27739000
C	-4.99947600	2.82254100	-0.05552700
C	-4.25045900	5.04693700	-0.93245200
H	-3.10491500	3.46605600	-2.92196500
H	-3.56612700	1.87721800	-2.24595200
H	-4.83210400	3.03483100	-2.75140800
H	-5.14840000	3.26222800	0.94504600
H	-5.95556400	2.90639700	-0.60759700
H	-4.75913400	1.75474300	0.07646200
H	-5.24932000	5.14823700	-1.40160400
H	-4.31241000	5.51833900	0.06305200
H	-3.55103800	5.63351300	-1.55162300
N	3.95695200	0.00314700	-1.41788000
H	4.40357200	0.14656700	-2.33480000
Cr	-2.51514000	0.10092000	1.53217300
O	-3.33816800	-0.37122000	3.27283300
O	-3.00036700	0.93815300	3.28804300

(H^{tBu}L)Cr(O$_2$) (^{B}2, S = 0, THF solvent)

P	2.20839100	3.01893400	-0.10912600
N	2.03984300	1.47528200	-0.53429900
C	1.12825100	3.46600000	1.34583700
C	0.20925400	2.56632600	1.96168400
C	-0.47629500	2.99047800	3.12444400
H	-1.18236000	2.29366000	3.58959600
C	-0.29202500	4.26539400	3.67391100
H	-0.84369100	4.55872600	4.57415800
C	0.59185100	5.15982700	3.05454900
H	0.74390800	6.16705900	3.45796200
C	1.29015200	4.75643300	1.90834900
H	1.97697900	5.46987000	1.44754000
C	-0.14950600	1.19855400	1.45546100
C	-1.14461400	1.07027400	0.46900400
H	-1.59233800	1.96971900	0.03426400
P	-4.97483800	-0.12733600	0.26565200
C	-3.87789200	-0.30696600	-1.21657200
C	-2.45422200	-0.34009400	-1.17593100
C	-1.73951100	-0.53072300	-2.38456400
H	-0.64205700	-0.54928000	-2.32552900
C	-2.38943000	-0.67821900	-3.61610800
H	-1.80324500	-0.82174400	-4.53092700
C	-3.79008000	-0.63940900	-3.66161800
H	-4.32373500	-0.75423000	-4.61186100
C	-4.51287100	-0.45857000	-2.47458200
H	-5.60415600	-0.44387200	-2.53381900
C	-1.56063800	-0.19813000	0.02474100
C	-0.94654200	-1.34231900	0.56241700
H	-1.24456800	-2.33369400	0.20506100
P	2.49352900	-2.87143900	-0.07659700
N	2.04386200	-1.44093300	-0.66144000
C	1.59142800	-3.32340300	1.49514500
C	0.63630800	-2.48191500	2.13834800
C	0.12727200	-2.87484500	3.39834400
H	-0.60323400	-2.22000800	3.88673300
C	0.50325300	-4.07795400	4.00919800
H	0.08385500	-4.35254100	4.98361900
C	1.39818400	-4.93319300	3.35103900
H	1.68647200	-5.89207600	3.79581900
C	1.93426900	-4.55115800	2.11339900
H	2.63612800	-5.23114800	1.62449900
C	0.05422100	-1.23241100	1.54752800
C	0.41872000	0.04064300	2.02279400
H	1.18880800	0.13350400	2.79646600
C	4.35671100	-2.75594800	0.40441100
C	5.13805000	-2.43808900	-0.89008300
C	4.45166300	-1.54851800	1.36489500
C	4.96662500	-3.98919800	1.10150100
H	5.18456200	-3.30463700	-1.57341700
H	4.68659500	-1.58271800	-1.42470500

H	6.17779800	-2.16714000	-0.62338600
H	3.90649800	-1.72846300	2.30871300
H	5.51576100	-1.37469100	1.61492800
H	4.04808200	-0.63485600	0.89672900
H	6.04392000	-3.78921100	1.26201400
H	4.52090900	-4.16980100	2.09370500
H	4.89102300	-4.91327900	0.50542000
C	2.08425800	-4.22836600	-1.38336000
C	0.58136900	-4.55907000	-1.24044700
C	2.31957100	-3.60595600	-2.77847500
C	2.91990200	-5.51931200	-1.25862000
H	0.34470200	-5.04956400	-0.28059600
H	-0.04323600	-3.65349800	-1.33486100
H	0.29222000	-5.25074300	-2.05480100
H	3.38295200	-3.37881100	-2.96419100
H	1.99383700	-4.33577600	-3.54456900
H	1.74341300	-2.67604600	-2.91228100
H	2.58451200	-6.22836500	-2.04042100
H	3.99547500	-5.33580000	-1.42292500
H	2.79182900	-6.02791500	-0.28784100
C	-5.90642000	1.55233000	0.03598400
C	-6.91089000	1.69131700	1.20225800
C	-4.81382100	2.63654800	0.18598400
C	-6.63380300	1.77625500	-1.30571200
H	-7.76533400	1.00042700	1.09901400
H	-6.43312600	1.51296900	2.18326900
H	-7.31606300	2.72181900	1.21574900
H	-4.07841900	2.59100300	-0.63776200
H	-5.28718900	3.63726000	0.15928900
H	-4.26828800	2.53201900	1.13884300
H	-7.12374600	2.77009400	-1.27918700
H	-5.93225100	1.78481000	-2.15698600
H	-7.42203900	1.03241700	-1.50780500
C	-6.20365300	-1.63752200	0.24247700
C	-5.37875000	-2.87568900	-0.17727400
C	-6.65500500	-1.83859000	1.70942200
C	-7.45509800	-1.51889500	-0.64916600
H	-5.10485800	-2.85537800	-1.24584200
H	-4.44995700	-2.96352500	0.41488600
H	-5.97968400	-3.78876200	0.00050900
H	-7.25480100	-0.99344700	2.09002800
H	-7.28393000	-2.74765100	1.77059600
H	-5.79014900	-1.98270800	2.37984300
H	-8.11251400	-0.68635400	-0.34521700
H	-7.21358100	-1.40162900	-1.71933200
H	-8.04667800	-2.45158300	-0.55603200
C	1.68908300	4.13053700	-1.58178200
C	2.74158300	3.95485900	-2.69942300
C	0.33580600	3.56457400	-2.07083500
C	1.51220100	5.62410000	-1.24235200
H	3.69057400	4.46408500	-2.45875800
H	2.95063200	2.89012500	-2.90436500

H	2.34884300	4.40712700	-3.63037800
H	-0.44796500	3.64577800	-1.29655700
H	0.00503100	4.15344300	-2.94777800
H	0.42496700	2.50751100	-2.37282700
H	1.24547900	6.16070400	-2.17361100
H	0.69263500	5.79292500	-0.52333600
H	2.42967100	6.09172400	-0.84744300
C	4.03839600	3.28185800	0.46916000
C	4.14843400	2.64080500	1.87193100
C	4.95283800	2.50429100	-0.50652700
C	4.50846700	4.74984200	0.52779800
H	3.56652300	3.18963300	2.63195000
H	3.81203400	1.58905400	1.86736900
H	5.21004700	2.65419500	2.18467900
H	5.06638200	3.02157300	-1.47373400
H	5.95849900	2.41905300	-0.05260000
H	4.56569500	1.48855200	-0.70331700
H	5.57876200	4.75767900	0.81239200
H	4.42783900	5.25724700	-0.44856500
H	3.97339400	5.34699100	1.28476800
N	-4.02707400	-0.14669000	1.56846200
H	-4.59928300	-0.06559800	2.42374700
Cr	2.17737800	0.05931900	-1.52677100
O	3.14821100	0.17581900	-3.05235000
O	1.70718700	0.11961100	-3.31059700

($H^{tBu}L$)Cr(O_2) (^{B}2, *S* = 1, THF solvent)

P	-2.41805300	2.94707500	0.00160100
N	-2.36681900	1.40145100	0.40320600
C	-1.07596600	3.39350100	-1.22101100
C	0.04271300	2.56024900	-1.51237000
C	1.04363800	3.05060700	-2.38736100
H	1.90372900	2.40298700	-2.59296300
C	0.95822000	4.31340200	-2.98569100
H	1.75055200	4.65521700	-3.66128400
C	-0.14839300	5.12885400	-2.71074100
H	-0.24124000	6.12109700	-3.16634300
C	-1.14068200	4.66880700	-1.83497400
H	-1.98185700	5.33160700	-1.62000600
C	0.30670300	1.17727400	-0.98617300
C	1.33022700	0.99389900	-0.03755500
H	1.82575900	1.86779000	0.39756000
P	5.11544200	-0.03861500	-0.26935000
C	4.18682000	-0.41699900	1.29254700
C	2.76852200	-0.51263600	1.41815800
C	2.22439400	-0.86519300	2.67763500
H	1.13435200	-0.93779100	2.76404600
C	3.02735900	-1.11747400	3.79696400
H	2.56439200	-1.38393100	4.75379300
C	4.42041000	-1.02910200	3.67439500
H	5.07324700	-1.22939200	4.53126000
C	4.97933500	-0.68773600	2.43560200
H	6.06844800	-0.64099200	2.35786000
C	1.74864100	-0.29887900	0.33450200
C	1.10749800	-1.41210900	-0.23628800
H	1.42196500	-2.42269700	0.04647200
P	-2.66105200	-2.83200500	0.01757900
N	-2.09751800	-1.53173900	0.78739700
C	-1.59238000	-3.24824400	-1.45359000
C	-0.48157400	-2.46103300	-1.87394200
C	0.22927600	-2.86009300	-3.03115800
H	1.08365900	-2.25089800	-3.34642300
C	-0.11653400	-4.00406700	-3.76133700
H	0.45948900	-4.28044300	-4.65172800
C	-1.19279500	-4.79335200	-3.33285800
H	-1.47249900	-5.70226500	-3.87715000
C	-1.91478000	-4.41258700	-2.19331100
H	-2.74585200	-5.04557400	-1.87300100
C	0.06828500	-1.24751300	-1.17607800
C	-0.30766500	0.04948400	-1.56663900
H	-1.08868500	0.18581100	-2.32173200
C	-4.43203400	-2.54375300	-0.69662000
C	-5.35700200	-2.24130900	0.50271900
C	-4.29979700	-1.28486600	-1.58572000
C	-5.04542200	-3.67270000	-1.55030700
H	-5.58464900	-3.14936400	1.08845800
H	-4.91703800	-1.49156100	1.18497700

H	-6.31711500	-1.83803800	0.12738500
H	-3.68876700	-1.48461100	-2.48448000
H	-5.30780800	-0.97903800	-1.92630000
H	-3.84414700	-0.43965800	-1.04162000
H	-6.07011000	-3.36486300	-1.83831300
H	-4.48522300	-3.83770300	-2.48555900
H	-5.13305000	-4.63117400	-1.01398500
C	-2.53190100	-4.32242300	1.24103400
C	-1.05015000	-4.76195400	1.26280600
C	-2.91104400	-3.79194700	2.64245800
C	-3.42794600	-5.53179700	0.90537800
H	-0.73937800	-5.23333700	0.31479300
H	-0.37593300	-3.91150900	1.46766800
H	-0.90914000	-5.50389700	2.07228400
H	-3.95037300	-3.42445800	2.68835700
H	-2.81588600	-4.62112700	3.36980400
H	-2.24503800	-2.97356900	2.95952500
H	-3.23231900	-6.32796100	1.65009600
H	-4.50233000	-5.28835400	0.96611900
H	-3.21949900	-5.96427800	-0.08774200
C	6.09611400	1.59711400	0.07977700
C	6.95949800	1.88923300	-1.16907100
C	5.01359600	2.69385500	0.20655700
C	6.97959700	1.64500800	1.34317700
H	7.81913800	1.20178600	-1.25630400
H	6.36993100	1.83031700	-2.10258500
H	7.36410000	2.91788800	-1.10132300
H	4.36902100	2.52755300	1.08857000
H	5.50650400	3.67751800	0.33174500
H	4.37003800	2.73812300	-0.68751900
H	7.49560400	2.62532000	1.37512200
H	6.38359200	1.56500000	2.26792300
H	7.75910400	0.86679700	1.35929800
C	6.31190700	-1.54132700	-0.58000300
C	5.52070400	-2.82190200	-0.22823700
C	6.59579400	-1.55434100	-2.10178100
C	7.65779700	-1.54089100	0.17258800
H	5.37036100	-2.93602600	0.85884500
H	4.52993800	-2.83498300	-0.71680800
H	6.08377200	-3.70578300	-0.58614900
H	7.15196800	-0.66204100	-2.43860500
H	7.21268900	-2.44106500	-2.34359600
H	5.66238200	-1.62503500	-2.68616800
H	8.29391400	-0.68228400	-0.10250600
H	7.54055900	-1.55150100	1.26940200
H	8.21725400	-2.45885600	-0.09740500
C	-2.08369000	4.04083100	1.54973400
C	-3.26746600	3.87561800	2.52920900
C	-0.81033300	3.45225300	2.19818200
C	-1.84292500	5.53346600	1.24585500
H	-4.18086500	4.38114300	2.17033800
H	-3.50106600	2.81233700	2.71715800

H	-2.99461300	4.33651000	3.49864200
H	0.06538800	3.52510600	1.52868100
H	-0.57901500	4.02659900	3.11605800
H	-0.95427600	2.39547600	2.47879800
H	-1.66159200	6.05798400	2.20439400
H	-0.95199600	5.69003200	0.61392900
H	-2.70449900	6.02479700	0.76411000
C	-4.11846900	3.35212700	-0.85177400
C	-4.02686500	2.84928100	-2.30987100
C	-5.19165300	2.52279400	-0.10911300
C	-4.55924500	4.83095300	-0.83226600
H	-3.33641000	3.45392800	-2.92218100
H	-3.69748100	1.79581300	-2.35792000
H	-5.02996400	2.90869500	-2.77490700
H	-5.32714200	2.84910300	0.93583900
H	-6.15974300	2.65271300	-0.63030600
H	-4.94516400	1.44890500	-0.10224000
H	-5.55177200	4.90448400	-1.31901300
H	-4.67112600	5.22073600	0.19341400
H	-3.88451200	5.50122200	-1.39006200
N	4.04282100	0.12154700	-1.46287700
H	4.53687300	0.27922200	-2.35503800
Cr	-2.50205100	0.03231700	1.53667100
O	-3.70464800	0.20129600	2.92271400
O	-2.27482800	0.14381400	3.33532300

(H^{tBu}L)Cr(O)$_2$ (2, *S* = 0, gas-phase)

P	2.62326700	-2.79607100	0.04353600
N	2.39578500	-1.25148300	0.37884700
C	1.43988200	-3.32801200	-1.29439200
C	0.33020800	-2.53608200	-1.70619900
C	-0.49283400	-3.02361800	-2.74955000
H	-1.34719900	-2.40787200	-3.05176500
C	-0.25056900	-4.24956600	-3.38018700
H	-0.90977200	-4.59205800	-4.18597500
C	0.83402400	-5.03440500	-2.96521800
H	1.03878300	-6.00211500	-3.43673400
C	1.66120100	-4.57128500	-1.93412400
H	2.49688000	-5.20071300	-1.61731900
C	-0.11081900	-1.22962700	-1.10790500
C	-1.12337800	-1.24494700	-0.13149300
H	-1.49332800	-2.20200200	0.25061800
P	-5.00541100	-0.07382200	-0.29222300
C	-4.10482000	-0.02847500	1.32374500
C	-2.68705700	-0.06005600	1.45997500
C	-2.12403300	-0.07372700	2.75926000
H	-1.02981600	-0.09864700	2.84596400
C	-2.92400100	-0.04521200	3.90854100
H	-2.45469500	-0.05485600	4.89884000
C	-4.31858600	0.00520100	3.78008800
H	-4.96297000	0.04010100	4.66594100
C	-4.89067300	0.01045700	2.50138100
H	-5.98032000	0.05039200	2.42066400
C	-1.68822000	-0.04794500	0.33983200
C	-1.21174600	1.17384300	-0.15782800
H	-1.64444500	2.11109500	0.20694000
P	2.37955700	2.95943400	0.05725200
N	1.95967600	1.54722900	0.67474800
C	1.19459100	3.40897200	-1.30512000
C	0.15592100	2.53755500	-1.73819800
C	-0.68616100	2.96513800	-2.79191800
H	-1.49295800	2.29184900	-3.10196200
C	-0.52849800	4.21118400	-3.41042100
H	-1.20073600	4.50907300	-4.22314500
C	0.48258100	5.07679700	-2.97026100
H	0.61397600	6.06339400	-3.42861900
C	1.32777900	4.67300400	-1.92863200
H	2.10466500	5.36455800	-1.59224900
C	-0.19750600	1.20932800	-1.13207800
C	0.32974500	0.00278800	-1.62651300
H	1.10196300	0.02048600	-2.40274000
C	4.11913100	2.90395600	-0.76560500
C	5.16835400	2.67223400	0.34677400
C	4.06740200	1.66543200	-1.69212300
C	4.51210500	4.13321600	-1.61195100
H	5.30557900	3.56874000	0.97692200
H	4.90614900	1.81819200	0.99614100

H	6.14496700	2.45448600	-0.12794300
H	3.34753500	1.80807000	-2.51854500
H	5.06857700	1.50963100	-2.13779600
H	3.78143400	0.75534400	-1.13886400
H	5.54192900	3.97714100	-1.98817000
H	3.85825900	4.25888000	-2.49088000
H	4.51575300	5.07318300	-1.03541100
C	2.19962900	4.30746900	1.42822600
C	0.68424600	4.57583700	1.58091000
C	2.72163500	3.68476500	2.74488500
C	2.94522800	5.63017600	1.15628700
H	0.25999200	5.09579200	0.70531600
H	0.12808200	3.63452500	1.73334600
H	0.52298600	5.21475100	2.47023200
H	3.79378700	3.43246600	2.70270000
H	2.57643600	4.41961000	3.56021500
H	2.17012400	2.76673900	3.00102800
H	2.74909000	6.32288500	1.99768300
H	4.03813900	5.49145800	1.09786300
H	2.60305800	6.13806200	0.23848100
C	-5.93202100	-1.77916000	-0.30363700
C	-6.75132200	-1.85609300	-1.61182300
C	-4.79912100	-2.83151600	-0.36163900
C	-6.84018200	-2.10550400	0.90033300
H	-7.63105600	-1.18839800	-1.59836500
H	-6.13502000	-1.60289900	-2.49365700
H	-7.12362700	-2.88894500	-1.75488900
H	-4.22092000	-2.85110400	0.57935800
H	-5.23870800	-3.83696800	-0.51008300
H	-4.09825800	-2.62024100	-1.18609700
H	-7.27891200	-3.11258100	0.75328300
H	-6.27114400	-2.13316300	1.84466600
H	-7.67929900	-1.39991400	1.01776700
C	-6.25921700	1.42056500	-0.28116100
C	-5.50151000	2.61743400	0.33900900
C	-6.53147400	1.74560200	-1.76951500
C	-7.61079900	1.22750400	0.43446000
H	-5.34457700	2.49483400	1.42373800
H	-4.51456300	2.75204800	-0.13797500
H	-6.08788100	3.54348700	0.18012000
H	-7.08626900	0.94103500	-2.28392400
H	-7.14336800	2.66579900	-1.83475500
H	-5.58761800	1.92315900	-2.31212000
H	-8.21645800	0.42698200	-0.02385600
H	-7.50659900	1.01072500	1.51113000
H	-8.19759400	2.16421000	0.35302800
C	2.26792300	-3.92816200	1.55403600
C	3.33320500	-3.65179400	2.63901500
C	0.88214700	-3.47467300	2.06988600
C	2.21226600	-5.43557600	1.22825400
H	4.31711000	-4.07919100	2.37597100
H	3.45735800	-2.57272600	2.83248000

H	3.00940100	-4.12762300	3.58441200
H	0.09241800	-3.66400500	1.32168800
H	0.63377700	-4.06165100	2.97544300
H	0.87074300	-2.40364900	2.33327000
H	1.98045700	-5.98403500	2.16183500
H	1.41456800	-5.67022700	0.50327800
H	3.16559900	-5.83560700	0.84368500
C	4.41367200	-3.01518600	-0.66681400
C	4.38946900	-2.44538800	-2.10385400
C	5.33798900	-2.13388000	0.20892300
C	4.98563100	-4.44895500	-0.68496800
H	3.79819100	-3.07213700	-2.79256200
H	3.97227800	-1.42404900	-2.12150600
H	5.42619300	-2.39708400	-2.48899700
H	5.48600400	-2.55986800	1.21476900
H	6.33047900	-2.07382800	-0.27840500
H	4.94350400	-1.11447500	0.34006800
H	6.02193600	-4.40328700	-1.07337600
H	5.03840100	-4.89257000	0.32349700
H	4.43070500	-5.13743600	-1.34423600
N	-3.91410300	0.05890900	-1.46132000
H	-4.34702300	0.01606500	-2.39539300
Cr	2.40805900	0.07881500	1.54922300
O	3.88011700	0.21013800	2.18851900
O	1.33678900	-0.19242200	2.72223500

(H^{tBu}L)Cr(O)$_2$ (2, *S* = 0, THF solvent)

P	2.57628000	-2.85496000	0.03375400
N	2.39936100	-1.30560000	0.41582900
C	1.34265400	-3.32855800	-1.28297000
C	0.22140400	-2.52665800	-1.64218900
C	-0.65413600	-3.00360500	-2.64808200
H	-1.51688600	-2.38198500	-2.91265300
C	-0.44920600	-4.22730200	-3.29693800
H	-1.14775600	-4.56024700	-4.07287100
C	0.65191200	-5.01810600	-2.94007800
H	0.83229700	-5.98175400	-3.42931500
C	1.52782500	-4.56755800	-1.94371000
H	2.37464700	-5.20406400	-1.67643900
C	-0.17655600	-1.20983700	-1.03600400
C	-1.21251800	-1.19132500	-0.08205800
H	-1.62655900	-2.13605200	0.28548000
P	-5.08410900	0.00268400	-0.30104500
C	-4.18480700	0.06744400	1.32073200
C	-2.76828300	0.04483300	1.48098800
C	-2.22775000	0.06257400	2.79009900
H	-1.13569300	0.03701800	2.89469200
C	-3.04184400	0.11244200	3.92869700
H	-2.58778400	0.12553300	4.92608300
C	-4.43447300	0.15262700	3.77593900
H	-5.09361200	0.20259300	4.64990800
C	-4.98636800	0.12789000	2.48797900
H	-6.07438200	0.16325600	2.39452500
C	-1.74274500	0.02567400	0.38356100
C	-1.20696500	1.23302000	-0.09567800
H	-1.61167300	2.18498800	0.26379700
P	2.54104700	2.91535700	0.04075000
N	2.11254400	1.51413300	0.69703500
C	1.33526100	3.36187600	-1.30875900
C	0.22928100	2.54280600	-1.67512100
C	-0.63368300	2.99419900	-2.70309000
H	-1.48927900	2.36263700	-2.96753000
C	-0.43117600	4.21123100	-3.36523600
H	-1.12044900	4.52595000	-4.15690500
C	0.65157200	5.02197100	-2.99713400
H	0.82508500	5.98391800	-3.49219300
C	1.51597300	4.59690900	-1.97947800
H	2.34431500	5.25315100	-1.70261300
C	-0.16959700	1.23446200	-1.05039600
C	0.32297200	0.00863200	-1.53683000
H	1.10217700	0.00029200	-2.30614300
C	4.26999700	2.80247500	-0.79163500
C	5.31374800	2.57017800	0.32462200
C	4.19810300	1.55285000	-1.69940100
C	4.68888800	4.00933000	-1.65602500
H	5.46944400	3.47444900	0.93852600
H	5.03346800	1.73216000	0.98739500

H	6.28432200	2.32122500	-0.14722400
H	3.47322800	1.68651000	-2.52290300
H	5.19395000	1.38523100	-2.15279400
H	3.91830700	0.64870300	-1.13385900
H	5.72423300	3.83291400	-2.00772500
H	4.05667200	4.11819100	-2.55284900
H	4.69006300	4.96307000	-1.10377600
C	2.39811900	4.29225000	1.39046000
C	0.89180500	4.59975000	1.55010100
C	2.91875800	3.69394800	2.71708500
C	3.17065400	5.59121000	1.08236100
H	0.47248900	5.11541600	0.66946600
H	0.30536900	3.68102500	1.72919500
H	0.75601500	5.26142000	2.42704600
H	3.99076000	3.43866100	2.67799400
H	2.78206100	4.44812900	3.51599500
H	2.35861200	2.78846500	2.99969200
H	2.98484000	6.30842400	1.90551600
H	4.26053300	5.42824000	1.03136300
H	2.83977900	6.08058100	0.15081800
C	-6.03689000	-1.68299800	-0.29354700
C	-6.85898200	-1.75162400	-1.60028800
C	-4.93756300	-2.76963700	-0.34046800
C	-6.95250400	-1.97369100	0.91350200
H	-7.71432100	-1.05393500	-1.59554000
H	-6.23739100	-1.53678300	-2.48889900
H	-7.26613800	-2.77407900	-1.72273500
H	-4.34116400	-2.78863900	0.58960300
H	-5.41328400	-3.76326000	-0.45306200
H	-4.24841600	-2.61482600	-1.18724000
H	-7.43348000	-2.96073200	0.76218700
H	-6.38307700	-2.03296800	1.85675200
H	-7.76095600	-1.23510100	1.03932200
C	-6.30449800	1.52120400	-0.33147100
C	-5.55018300	2.71562300	0.29643200
C	-6.54337600	1.83802400	-1.82741900
C	-7.67295100	1.35020100	0.35749600
H	-5.42101400	2.60507300	1.38659900
H	-4.55121900	2.85077800	-0.15606100
H	-6.12797500	3.64320700	0.11707700
H	-7.09327800	1.03496600	-2.34908500
H	-7.15011500	2.76068200	-1.90551700
H	-5.59223700	2.01182100	-2.35936800
H	-8.27877500	0.55331100	-0.10642700
H	-7.59597400	1.14649000	1.43889200
H	-8.24276100	2.29493500	0.25068400
C	2.20811300	-3.99525600	1.53532400
C	3.30288500	-3.77385700	2.60195200
C	0.84947500	-3.50293000	2.08394500
C	2.09685400	-5.49418500	1.18973800
H	4.26813700	-4.22276900	2.30994900
H	3.46354800	-2.70267300	2.81398500

H	2.98326300	-4.25789500	3.54490900
H	0.03643700	-3.65202500	1.35145800
H	0.59727400	-4.09372300	2.98611500
H	0.88182800	-2.43581600	2.36386200
H	1.86602000	-6.04701900	2.12129300
H	1.27779900	-5.69672800	0.47877900
H	3.02916000	-5.91846600	0.78182700
C	4.34471100	-3.11914900	-0.71489200
C	4.31492700	-2.55260600	-2.15222100
C	5.31502300	-2.27220800	0.14261900
C	4.86575400	-4.57157400	-0.73626000
H	3.69321400	-3.16146000	-2.83023900
H	3.93853500	-1.51479200	-2.17635600
H	5.34640700	-2.54607400	-2.55382400
H	5.47332000	-2.71111800	1.14183200
H	6.29688200	-2.24259000	-0.36828400
H	4.95993500	-1.23935500	0.28309000
H	5.89666000	-4.55853900	-1.14145000
H	4.91960200	-5.01207100	0.27328100
H	4.27941400	-5.24410600	-1.38406200
N	-3.99065900	0.08738800	-1.48487100
H	-4.46984200	0.04664200	-2.39861700
Cr	2.48895600	0.02905400	1.57028000
O	3.96717500	0.09266700	2.21832300
O	1.40104400	-0.18656600	2.74836500
